# Radiopharmaceuticals and their applications in medicine

**DOI:** 10.1038/s41392-024-02041-6

**Published:** 2025-01-03

**Authors:** Siqi Zhang, Xingkai Wang, Xin Gao, Xueyao Chen, Linger Li, Guoqing Li, Can Liu, Yuan Miao, Rui Wang, Kuan Hu

**Affiliations:** 1 State Key Laboratory of Bioactive Substance and Function of Natural Medicines, Institute of Materia Medica, Chinese Academy of Medical Sciences and Peking Union Medical College, 100050 Beijing, China; 2 Key Laboratory of Preclinical Study for New Drugs of Gansu Province, School of Basic Medical Sciences & Research Unit of Peptide Science, Chinese Academy of Medical Sciences, Lanzhou University, 2019RU066, 730000 Lanzhou, China

**Keywords:** Diagnostics, Drug development

## Abstract

Radiopharmaceuticals involve the local delivery of radionuclides to targeted lesions for the diagnosis and treatment of multiple diseases. Radiopharmaceutical therapy, which directly causes systematic and irreparable damage to targeted cells, has attracted increasing attention in the treatment of refractory diseases that are not sensitive to current therapies. As the Food and Drug Administration (FDA) approvals of [^177^Lu]Lu-DOTA-TATE, [^177^Lu]Lu-PSMA-617 and their complementary diagnostic agents, namely, [^68^Ga]Ga-DOTA-TATE and [^68^Ga]Ga-PSMA-11, targeted radiopharmaceutical-based theranostics (radiotheranostics) are being increasingly implemented in clinical practice in oncology, which lead to a new era of radiopharmaceuticals. The new generation of radiopharmaceuticals utilizes a targeting vector to achieve the accurate delivery of radionuclides to lesions and avoid off-target deposition, making it possible to improve the efficiency and biosafety of tumour diagnosis and therapy. Numerous studies have focused on developing novel radiopharmaceuticals targeting a broader range of disease targets, demonstrating remarkable in vivo performance. These include high tumor uptake, prolonged retention time, and favorable pharmacokinetic properties that align with clinical standards. While radiotheranostics have been widely applied in tumor diagnosis and therapy, their applications are now expanding to neurodegenerative diseases, cardiovascular diseases, and inflammation. Furthermore, radiotheranostic-empowered precision medicine is revolutionizing the cancer treatment paradigm. Diagnostic radiopharmaceuticals play a pivotal role in patient stratification and treatment planning, leading to improved therapeutic outcomes in targeted radionuclide therapy. This review offers a comprehensive overview of the evolution of radiopharmaceuticals, including both FDA-approved and clinically investigated agents, and explores the mechanisms of cell death induced by radiopharmaceuticals. It emphasizes the significance and future prospects of theranostic-based radiopharmaceuticals in advancing precision medicine.

## Introduction

Radiopharmaceuticals involve the accurate delivery of radionuclides to targeted cells through vectors including small molecules, peptides and antibodies.^1,2^ In combination with positron emission tomography (PET) and single-photon emission computed tomography (SPECT) scans, radiopharmaceuticals enable rapid and precise monitoring of whole-body disease lesions, thus allowing accurate patient stratification in a noninvasive way.^1^ Moreover, the precise deposition of high energy emitted by radionuclides in target cells directly induces cell killing through single- or double-strand DNA breaks.^3^ In contrast to radiotherapy, which involves an external radiation source, radiopharmaceutical therapy (RPT) restricts radiation within targeted cells and exhibits few toxic effects on non-targeted cells, thereby reducing normal organ injury. Notably, compared with conventional modalities, a small dose of targeted vectors could achieve sufficient radiation to achieve cell killing, enabling a safe and economical therapeutic modality.^4–6^ Radiopharmaceuticals make it possible to visualize and identify drug accumulation in lesions, allowing clinicians to treat the disease when “seeing” them and achieving personalized treatment, which is one of the major advantages.

**Fig. 1:**
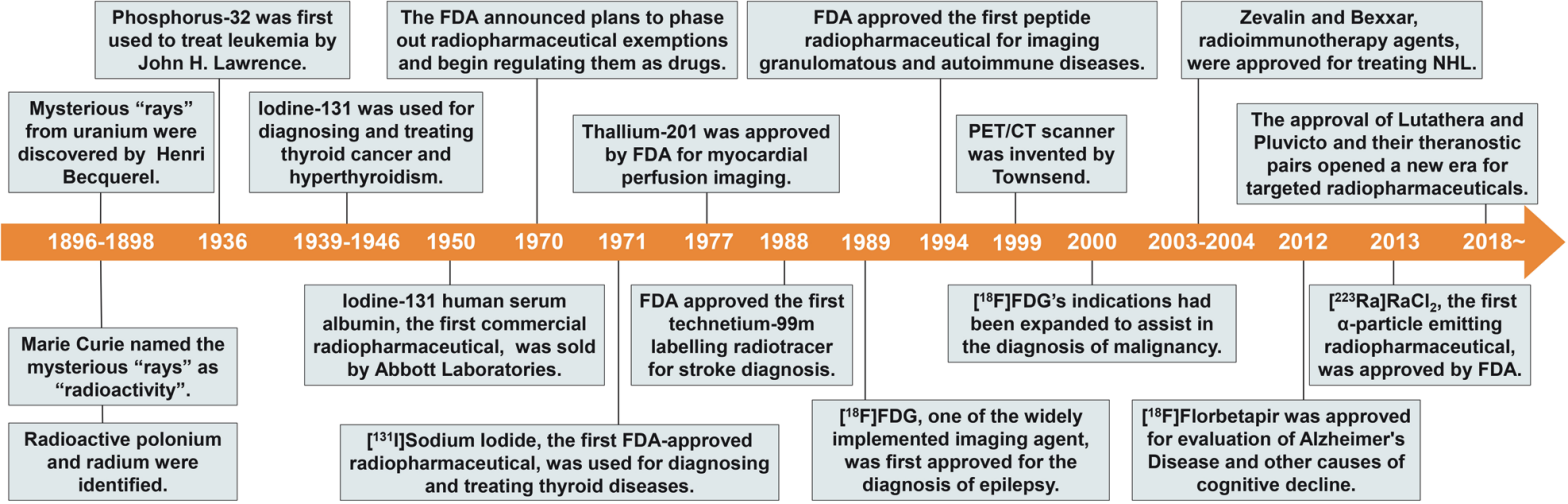
Overview of significant milestones and regulatory approvals for the discovery of radiopharmaceuticals. The journey began in 1896 with Henri Becquerel’s accidental discovery of “rays” emitted from uranium, a phenomenon later termed “radioactivity” by Marie Curie. Curie’s research further proceeded in the field by discovering the radioactive elements polonium and radium in 1898. In 1936, John H. Lawrence first used phosphorus-32 for the treatment of leukaemia which was the first example of the use of radionuclides in medicine. The commercial and regulatory landscape for radiopharmaceuticals began to take shape with the sale of the first commercial radiopharmaceutical, iodine-131 human serum albumin (RISA), by Abbott Laboratories. This regulatory evolution continued with the FDA’s decision to phase out the exemption for radiopharmaceuticals and regulate them as generic drugs. [^131^I]Sodium iodide was approved by FDA for treating thyroid disease. Technological and clinical advances further accelerated with the FDA’s approval of thallium-201 for myocardial perfusion imaging and the introduction of the first technetium-99m labelled radiopharmaceutical ([^99m^Tc]Tc-exametazime) for stroke diagnosis. [^18^F]FDG was first approved for identifying regions of abnormal glucose metabolism associated with foci of epileptic seizures in 1989. In 1999, the PET/CT scanner was invented by Dr. Townshend, who combined precise imaging with detailed anatomical information and improved diagnostic accuracy. On March 12, 2000, the FDA published a notice that expanded the approval of [^18^F]FDG for new indications. The therapeutic scope of radiopharmaceuticals was further expanded with the approval of the radioimmunotherapy drugs Zevalin and Bexxar for treating NHL. [^18^F]Florbetapir, the first Aβ-specific PET radiotracer approved by the FDA in 2012, was used for the evaluation of patients with cognitive impairment. Bayer’s [^223^Ra]RaCl_2_, the first α-particle radiopharmaceutical, markedly improved the treatment of metastatic cancers. Lutathera was the first FDA-approved radiopharmaceutical for targeted RPT in 2018. Another targeted RPT, Pluvicto, was approved for prostate cancer and achieved near-blockbuster status in 2023

The combination of precise diagnosis with efficient targeted RPT is defined as radiotheranostic. Since the development of radium-223 and iodine-131 for cancer treatment, research on radiopharmaceuticals has been conducted for at least 80 years (Fig. 1). Thus far, more than 60 radiopharmaceuticals have been approved for the diagnosis or treatment of various cancers, neurodegenerative disorders, and cardiovascular diseases.^7^ Since the approval of Lutathera ([^177^Lu]Lu-DOTA-TATE), Pluvicto ([^177^Lu]Lu-PSMA-617) and their complementary diagnostic imaging agents, Netspot ([^68^Ga]Ga-DOTA-TATE), and Locametz ([^68^Ga]Ga-PSMA-11), targeted radiopharmaceuticals have attracted increasing attention from clinicians and researchers because of their remarkable performance in cancer treatment, especially for patients with refractory and metastatic cancers who gain limited benefit from chemotherapy or other current therapeutic modalities.

Great progress has been achieved in recent years in terms of incorporation into targeting vectors with high target binding affinity, and success in the large-scale production of novel imaging and therapeutic radionuclides. Numerous studies have attempted to modify and optimize currently approved radiopharmaceuticals, as well as to develop novel targeting ligands with high binding potential to disease targets. Efficient chemical strategies have been introduced to improve their binding affinity, in vivo stability, and pharmacokinetic (PK) properties.^8–10^ Some radiopharmaceutical agents have been evaluated in healthy humans and patients and have shown superior outcomes in clinical trials. With respect to radionuclides, therapeutic isotopes with higher linear energy transfer (LET) and longer half-lives have been introduced to RPTs in recent years, as the major demand for radiopharmaceutical discovery has shifted from diagnostic imaging to targeted therapy. α-Emitters with short emission ranges and greater energy deposition are emerging as promising therapeutic radionuclides because of their high anti-tumour efficacy and minimal toxicity to normal cells. Radiopharmaceuticals labelled with α-emitters, including actinium-225, astatine-211 and lead-212, have been evaluated in patients with various cancers and have exhibited robust anti-tumour effects in clinical trials.^1^^,11^

Generally, RPT-induced cell killing is based on evidence from radiotherapy. However, the difference between RPT and radiotherapy remains unclear. In addition, there is no complete explanation of the mechanisms underlying the cell-killing effect caused by radiopharmaceuticals. For example, the major pathways that cause radiation-induced cell death, apoptosis, pyroptosis, senescence, or other biological processes are unknown.^12,13^ Recent studies have focused on the tumour immune microenvironment, which influences the anti-tumour effects of radiopharmaceuticals. These studies reported that radiopharmaceuticals increased the immunogenicity of tumour tissues, which was indicated by the increase in the number of active immune cells that infiltrated into tumour microenvironment after RPT.^14–16^ However, it is critical to determine how the tumour immune microenvironment is stimulated and whether this process is unique to RPTs compared with radiotherapy.

This review aims to highlight the importance of radiopharmaceutical-based theranostics through a comprehensive overview of the clinical and preclinical milestones achieved thus far. We also discuss the current challenges associated with the development of next-generation radiotheranostic modalities with high efficiency and few detrimental effects and provide new insights into the perspectives and future potential of radiopharmaceutical discovery.

## Evolution of nuclear medicine: radionuclides, devices and radiotheranostics

There are three elements that drive the development of nuclear medicine: radionuclides, devices, and new drugs. In the first half of the 20^th^ century, the discovery of new radionuclides, from natural to artificial radionuclides, laid the foundation for nuclear medicine. The clinical use of radionuclides has spurred the demand for related diagnostic tools. In the second half of the 20^th^ century, the era of inventing nuclear medicine devices and their applications began.^17^ The advancements, from gamma cameras to PET/CT, have enabled nuclear medicine to serve patients better. The development of new drugs, based on the availability of radionuclides and devices, is also valuable. The emergence of a range of novel biotechnologies is driving the rapid development of new drugs that can be used in radiopharmaceuticals. With the approval of [^177^Lu]Lu-DOTA-TATE and [^68^Ga]Ga-DOTA-TATE, the era of radiotheranostics, centred around radiopharmaceuticals, has fully arrived.

### Radionuclides for nuclear medicines

The radionuclide collection published by the International Commission on Radiological Protection (ICRP) lists approximately 1200 radionuclides, but only a few dozen of them are used in clinical and scientific research,^18^ presented in Table 1. Radionuclides have different physical and biochemical characteristics, such as half-lives, decay modes, energies of radiation, retention of radioactivity in the tumour, and applications.^19,20^ And we also show their common production methods. According to their decay modes, radionuclides can be classified into α-emitters, β-emitters, γ-emitters, and Auger electron emitters. In clinical applications, radionuclides can be divided into two major groups: imaging isotopes and therapeutic isotopes.

**Table 1:** Radionuclide properties^553^

Type	Radionuclide	Half-life	Radiation	Production	Application
Positron emitters	Carbon-11	20.36 m	β +	Accelerator	PET Imaging
	Nitrogen-13	9.97 m	β +	Accelerator	PET Imaging
	Oxygen-15	2.13 m	β +	Accelerator	PET Imaging
	Fluorine-18	109.77 m	β +	Accelerator	PET Imaging
	Copper-61	3.34 h	β +	Accelerator	PET Imaging
	Copper-64	12.70 h	β +	Accelerator	PET Imaging
	Gallium-66	9.49 h	β +	Accelerator	PET Imaging
	Gallium-68	67.71 m	β +	Generator	PET Imaging
	Rubidium-82	1.26 m	β +	Generator	PET Imaging
	Zirconium-89	78.41 h	β +	Accelerator	PET Imaging
	Iodine-124	4.17 d	β +	Accelerator	PET Imaging
γ-emitters	Gallium-67	3.26 d	γ	Accelerator	SPECT Imaging, Auger therapy
	Technetium-99m	6.00 h	γ	Generator	SPECT Imaging
	Indium-111	2.80 d	γ	Accelerator	SPECT Imaging, Auger therapy
	Iodine-123	13.22 h	γ	Accelerator	SPECT Imaging
β-emitters	Phosphorus-32	14.27 d	β-	Reactor	β-Therapy
	Scandium-47	3.35 d	β-	Accelerator	β-Therapy, SPECT Imaging
	Copper-67	61.83 h	β-	Accelerator	β-Therapy, SPECT Imaging
	Strontium-89	50.56 d	β-	Reactor	β-Therapy
	Yttrium-90	64.05 h	β-	Reactor	β-Therapy
	Iodine-131	8.03 d	β-	Reactor	β-Therapy, SPECT Imaging
	Holmium-166	26.82 h	β-	Reactor	β-Therapy, SPECT Imaging
	Lutetium-177	6.65 d	β-	Reactor	β-Therapy
	Rhenium-186	3.72 d	β-	Reactor	β-Therapy
	Rhenium-188	17.00 h	β-	Reactor	β-Therapy, SPECT Imaging
	Lead-212	10.62 h	β-	Reactor	α/β-Therapy
α-emitters	Astatine-211	7.21 h	α	Accelerator	α-Therapy
	Bismuth-213	45.59 m	α	Generator	α-Therapy
	Actinium-225	9.92 d	α	Reactor	α-Therapy
	Radium-223	11.43 d	α	Generator	α-Therapy
	Thorium-227	18.70 d	α	Generator	α-Therapy
Auger electron emitters	Bromine-77	57.04 h	Auger electron	Accelerator	Auger therapy
	Iodine-125	59.41 d	Auger electron	Reactor	Auger therapy
	Platinum-191	2.83 d	Auger electron	Reactor	Auger therapy

**Fig. 2:**
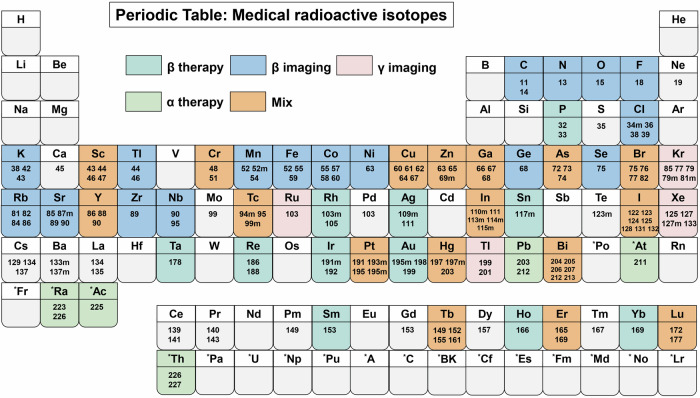
List of radioactive elements in the periodic table. Elements that emit radiation are called radionuclides. They are classified as positron emitters, α-emitters, β-emitters, γ-emitters, Auger electron emitters and hybrid nuclides according to their different forms of decay and have different clinical roles. In this figure, they are presented in different colours. Due to the difference in mass numbers, these radionuclides often exhibit significant variations in their physical properties and applications. There are 11 elements used for β-therapy, whereas α-therapy is less common, with five elements currently used in clinical settings. A small number of auger electron emitters, such as platinum-191, indium-111, and iodine-125, can also be used for therapy.β-imaging comprises the majority, with 17 elements used, followed by γ-imaging, which involves four elements. In addition, hybrid nuclides include several elements that can emit different types of radiation. These radionuclides have mixed applications that can simultaneously provide imaging and therapeutic functions. The rich variety of medical isotopes provides infinite possibilities for radiopharmaceuticals

Radionuclide imaging is typically categorized into two primary modalities: SPECT and PET imaging.^21^ The corresponding nuclides are single-photon emitters and positron emitters. These nuclides have shorter half-lives, which helps reduce the radiation burden of the patients. Common imaging radionuclides are shown in Fig. 2. Therapeutic radionuclide selection should be considered on the basis of several crucial aspects. First, the radionuclide should have an appropriate physical half-life for the desired therapeutic application; a suitable range of the half-life for therapeutic radionuclides is between 6 h and 10 days.^22^ Second, the radionuclides should emit high LET radiation to kill the tumour cells.^23^ On the basis of these characteristics, therapeutic radionuclides can be classified into three types: β-emitters, α-emitters, and Auger electron emitters.

#### Single-photon emitters

Single-photon radionuclides only emit a single photon during their decay process. These radionuclides are commonly used in SPECT.^24^ In clinical practice, these radionuclides have diverse imaging applications, including cancer, cardiovascular and neurological applications. Among them, technetium-99m is particularly notable and is utilized in approximately 80% of SPECT procedures conducted in nuclear medicine.^25^ This prevalence is due to its favourable γ-ray energy of 140.5 keV, which has a minimal risk of toxicity and a suitable half-life of 6 h. Both technetium-99m and iodine-123 have received approval from the FDA for use in medical imaging.

#### Positron emitters

Positron radionuclides exhibit distinctive properties in terms of the directionality and simultaneity of photons produced during annihilation. The two photons created through annihilation are generated simultaneously, and travel in opposite directions from each other at approximately 180°, which shows their directionality.^26^ The most commonly used positron emitters include carbon-11, nitrogen-13, oxygen-15, fluorine-18, iodine-124, copper-64, gallium-66, and gallium-68. Fluorine-18 is frequently used in the radiolabelling of molecules for PET because of its distinctive advantages over other positron nuclides.^27^ These advantages include (1) a low positron range that enables the creation of PET images with high spatial resolution and (2) a clear positron emission profile with 3% electron capture and 97% positron emission.^28^ Fluorine-18 is widely used to label metabolic markers, such as [^18^F]FDG, a glucose analogue with high uptake in cells exhibiting elevated metabolic activity, notably cancer cells. [^18^F]FDG has been termed “the molecule of the century” and has opened the door to PET/CT imaging and the development of positron nuclides.^29^

#### β-emitters

β-emitters are radioactive nuclei that decay by emitting a β particle, which is essentially an electron. During β decay, a neutron within the nucleus is converted into a proton, which releases an electron and an antineutrino.^30^ β-emitters, the most commonly used radioactive nuclides in clinical practice, are cytotoxic against relatively large cancer deposits due to their emission of high-energy electrons in the medium tissue range (0.05–12 mm). The β radionuclides approved by the FDA for use in radiotherapy include yttrium-90, lutetium-177, iodine-131, and strontium-89. Currently, lutetium-177 is gaining increasing attention with the FDA-approved [^177^Lu]Lu-DOTA-TATE (Lutathera®) in 2018 and [^177^Lu]Lu-PSMA-617 in 2022.^31,32^ The low-energy β particles emitted by lutetium-177 can effectively target tumour tissues while sparing surrounding normal tissues. These characteristics make lutetium-177 one of the most promising therapeutic radionuclides. However, the use of β-emitting radionuclides has several limitations. β-emitters have relatively low energy and poor tissue penetration, resulting in lower treatment efficacy for larger and malignant tumours. Consequently, many researchers have focused their attention on α-nuclides.^33^

#### α-emitters

α-emitters are radioactive nuclei that decay by emitting an α particle, which consists of two protons and two neutrons, essentially a helium-4 nucleus. Compared with the currently used β-emitting radionuclides for tumour radiotherapy, α-emitting radionuclides possess the following characteristics: (1) a shorter range of α particles; (2) higher energy of emitted α radiation; and (3) irreversible damage to DNA caused by α radiation.^34^ With important clinical breakthroughs in targeted radionuclide therapy, α-emitting radionuclides have also been successfully used in research and clinical applications. The clinical approval of [^223^Ra]RaCl_2_ (Xofigo) represents a significant milestone in the development and application of α-emitting radiopharmaceuticals.^35^ Presently, the only α emitter approved by the FDA is radium-223. Owing to the excellent therapeutic advantages of α-nuclides, other promising α-emitters, such as astatine-211, actinium-225, and thorium-227, are also emerging. However, on the basis of the current research, most of the α-nuclides intended for therapy are still in the preclinical stage. The following issues related to the source of α-nuclides need to be addressed: first, most of the high-mass, pure α-emission radionuclides that can be used for targeted therapies have high production costs and a limited production ability, which restricts many systematic experiments; second, the labelling methods need to be improved, which requires the development of more efficient chelators to strengthen the labelling stability further; third, a quantification method is needed to conduct extensive research on the dosimetric method and algorithm.

#### Auger electron emitters

Auger electron emitters are low-energy electrons produced by non-radiative transitions, and their range of action is much lower than that of α-particles and β-particles; thus, they can directly locate the lesion without damaging the surrounding cells.^36^ These particles have an LET of 4-26 keV/m and a tissue range smaller than the diameter of a single cell, making them ideal for nucleus targeting.^37,38^ The representative radionuclides are bromine-77, iodine-125, indium-111, platinum-191, and gallium-67. Notably, iodine-125, indium-111, and gallium-67 can be classified as both Auger electron emitters and γ-emitters based on the type of decay. Among them, the main decay of gallium-67 and indium-111 is the γ-emitter, and for iodine-125 the main decay is the Auger electron. Thus, gallium-67 and indium-111 are listed as γ-emitters, and iodine-125 is listed as an Auger electron emitter in Table 1. Notably, because of their short radiation range, the requirements for their targeting molecules are unique, and the targeting molecules should be able to deliver the nuclide to the targeted cell nucleus, thereby breaking the DNA.

### Nuclear imaging devices

In addition to discovering and producing medical radionuclides, the development of imaging devices plays a crucial role in radiopharmaceuticals. Significant progress has been made in the development of imaging devices, from the early use of scintillation detectors to assess iodine-131 uptake in the thyroid to Hal Anger’s invention of the gamma camera in the 1950s, followed by its widespread adoption and advancements in subsequent decades, including the use of collimation technology for whole-organ imaging.^39^ Currently, the most commonly used imaging modality in nuclear medicine is SPECT, which employs single photons for imaging.^40^ However, its resolution is limited because it relies on single γ detection. This led to the development of PET, which acquires images by detecting two photons generated by the annihilation reaction between emitted positrons and electrons within the body.^41,42^ To increase the quality of molecular imaging, a series of hybrid imaging devices have emerged, such as SPECT/CT, PET/CT, PET/magnetic resonance imaging (MRI), whole-body scanning, and even tri-modality devices, which collectively provide more precise instruments for nuclear medicine diagnosis.^43,44^ The advancements in imaging technology will continue, and the question remains: what will be the next generation of even more precise and clearer imaging devices?

### Radiotheranostics

“Theranostics” is a term derived from the combination of “therapeutics” and “diagnostics.” Theranostics involving the use of radiopharmaceuticals are referred to as “radiotheranostics.” This term uses the same molecule for imaging first and then therapy by conjugating therapeutic radionuclides to target the examined lesions. Because the diagnostic molecule helps refine the treatment plan for each patient, such as selecting appropriate treatment options and adjusting dosages, theranostics can contribute to the development of personalized medicine.^45,46^ Between 2016 and 2018, the FDA successively approved [^68^Ga]Ga-DOTA-TATE and [^177^Lu]Lu-DOTA-TATE for the diagnosis and treatment of well-differentiated neuroendocrine tumours (NETs).^47^ In 2022, [^177^Lu]Lu-PSMA-617 was approved for the treatment of metastatic castration-resistant prostate cancer (mCRPC) patients,^32^ increasing the popularity of the theranostic approach in the field of nuclear medicine and indicating promising directions for its future development. Among these isotopes, lutetium-177 and gallium-68 have been widely utilized radionuclides in recent years and have served as excellent partners for theranostics. In the future, it might be possible to develop a radiopharmaceutical that can simultaneously conduct diagnosis and therapy, thereby enhancing the convenience of radiotheranostics and truly realizing the concept of “treating what you see.” Furthermore, this technology could be applied to multiple types of cancer.

## FDA-approved Radiopharmaceuticals

**Fig. 3:**
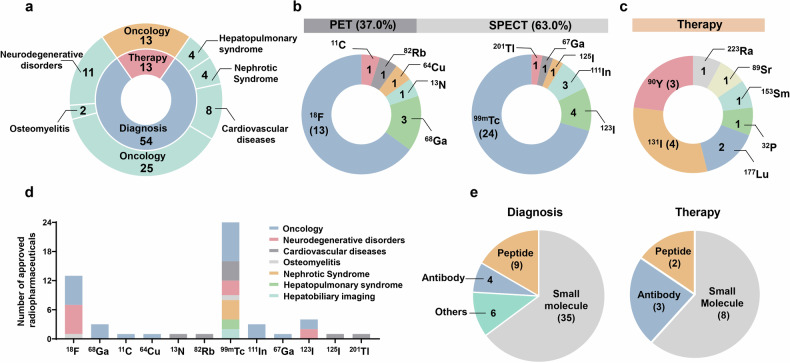
Summary of approved radiopharmaceuticals. (**a**) Approval radiopharmaceuticals used in diagnosis and therapy for different diseases. Diagnostic agents are categorized into seven categories on the basis of indications. Several agents are used in multiple-diseases ([^18^F]FDG, for example), and they are preferentially categorized into their primary indications. All therapeutic radiopharmaceuticals are applied for oncology. (**b**) Radionuclides used in PET (37.0%) and SPECT (63.0%) scanning. Fluorine-18, gallium-68, carbon-11, nitrogen-13, copper-64, and rubidium-82 labelled agents are approved for PET/CT diagnosis. For SPECT/CT imaging, technetium-99m, iodine-123, indium-111, gallium-67, iodine-125, and thallium-201 are used. (**c**) Radionuclides used in cancer therapy, including iodine-131, yttrium-90, lutetium-177, phosphorus-32, strontium-89, samarium-153, and radium-223. (**d**) Numbers of approved diagnostic radiopharmaceuticals used in various diseases catalogued by radionuclides. Technetium-99m is mostly used in clinical imaging for multiple diseases. Fluorine-18 is used mainly in oncology and neurodegenerative disorders. (**e**) Targeting vectors for diagnostic and therapeutic radiopharmaceuticals. Small molecules are used as the major vectors for radiopharmaceuticals discovery. Peptides play a distinct role both in diagnosis and therapy, particularly after the FDA approval of [^68^Ga]/[^177^Lu]Ga-DOTA-TATE for NETs. Antibodies play essential roles in both imaging and therapy because of their strong binding affinity in vivo. Others indicate protein and serum albumin-based radiopharmaceuticals. The number of approved radiopharmaceuticals in each catalogue is presented

According to our research, 67 radiopharmaceuticals are currently approved worldwide, of which 54 are used for disease diagnosis and 13 for therapy (Fig. 3).

### Radiopharmaceuticals for imaging

Marketed radiopharmaceuticals for imaging are widely used for the diagnosis of malignant cancer, neurodegenerative disorders, cardiovascular disease, and other diseases (such as nephrotic syndrome and hepatopulmonary syndrome). With respect to the types of radiation detectors used, 34 SPECT radiopharmaceuticals and 20 PET radiopharmaceuticals are clinically used. For radionuclides for SPECT detection, technetium-99m accounts for 70.6%, and iodine-123, indium-111, gallium-67, iodine-125, and thallium-201 account for 11.8%, 8.8%, 2.9%, 2.9%, and 2.9% respectively. For radionuclides for PET detection, fluorine-18 accounts for the majority (65.0%) due to its suitable half-life (109.8 min), flexible production from cyclotrons, and a well-developed labelling method. In addition, gallium-68, carbon-11, nitrogen-13, copper-64, and rubidium-82 account for 15.0%, 5.0%, 5.0%, 5.0%, and 5.0% respectively. Notably, gallium-68 has potential applications in the future for combination with lutetium-177 or yttrium-90 as a theranostic pair, especially in the development of PSMA-targeting radiopharmaceuticals.^48,49^ Copper-64 has also received increasing attention because of its long half-life (12.8 h), high resolution and detection rate in PET/CT imaging, and high safety. These characteristics make copper-64 suitable for labelling antibodies with high binding affinity and facilitating transportation to distant hospitals for drug preparation and clinical use^50,51^ (Table 2).

**Table 2:** Radiopharmaceutical imaging agents approved wordwide

Disease	PET	SPECT
Oncology	2-[^18^F]Fluoro-2-Deoxy-D-Glucose ([^18^F]FDG)	[^99m^Tc]Tc-DTPA-Tilmanocept
	[^18^F]Flotufolastat	[^99m^Tc]Arcitumomab
	[^18^F]Fluciclovine	[^99m^Tc]TcN-pyridoxyl-5-methyltryptophan
	[^18^F]PSMA-1007	[^99m^Tc]TcECG
	[^18^F]Piflufolastat (DCFPyL)	[^99m^Tc]TcSulfur Colloid
	[^18^F]Fluoroestradiol (FES)	[^99m^Tc]TcPhytate
	[^68^Ga]Ga-DOTA-TATE	[^99m^Tc]TcMDP
	[^68^Ga]Ga-DOTA-TOC	[^99m^Tc]TcGH
	[^68^Ga]Ga-PSMA-11	[^111^In]Capromab pendetide
	[^11^C]Choline	[^111^In]In-DTPA-Octreotide
	[^64^Cu]Cu-DOTA-TATE	[^111^In]Satumomab pendetide
		[^123^I]MIBG
		[^123^I]Sodium iodinate
		[^67^Ga]Gallium citrate
Neurology	[^18^F]Florbetaben	[^99m^Tc]NaTcO_4_
	[^18^F]Flutemetamol	[^99m^Tc]TcHMPAO
	[^18^F]Florbetapir	[^99m^Tc]TcECD
	[^18^F]Flortaucipir	[^123^I]Iomazenil
	[^18^F]FDOPA	[^123^I]FP-CIT
	[^18^F]FP-CIT	
	[^18^F]FDG	
Cardiology	[^18^F]FDG	[^99m^Tc]Tetrofosmin
	[^13^N]NH_3_·H_2_O	[^99m^Tc]Sestamibi
	[^82^Rb]Rubidium chloride	[^99m^Tc]TcRBCs
		[^99m^Tc]TcPYP
		[^125^I]Iodinated albumin
		[^201^Tl]Thallium chloride
Bone imaging	[^18^F]NaF	[^99m^Tc]Sulesomab
Nephrotic Syndrome	--	[^99m^Tc]TcDTPA^2-^
		[^99m^Tc]TcSuccimer
		[^99m^Tc]TcMAG3
		[^99m^Tc]Human serum albumin diethylenetriamine pentaacetic acid
Hepatopulmonary imaging	--	[^99m^Tc]Albumin Aggregate Injection
		[^99m^Tc]Diethylenetriaminepentaacetic acid-galactosyl-human serum albumin
		[^99m^Tc]Macroaggregated human serum albumin
		[^99m^Tc]TcMebrofenin

#### Radiopharmaceuticals for cancer imaging

The approved radiopharmaceuticals used for disease diagnosis can be divided into seven main fields: tumour imaging (46.3%), central nervous system (CNS) imaging (20.4%), cardiovascular imaging (14.8%), renal imaging (7.4%), lung imaging (3.7%), liver imaging (3.7%), and bone imaging (3.7%). Because of their special characteristics, radionuclides can emit radiation when enclosed in a targeted cell, and it is not necessary for them to be inside the cell cytoplasm. Radiopharmaceuticals work either extracellularly by binding to receptors on the cell membrane surface (PSMA, PD-L1, somatostatin receptor (SSTR), αvβ6, etc.) or intracellularly through endocytosis.^52^ Molecular imaging of tumours involves various radiopharmaceuticals on the basis of their specific binding affinity to certain targets: 1) PSMA-targeting radiopharmaceuticals such as [^68^Ga]Ga-PSMA-11 and [^18^F]DCFPyL^53,54^ and 2) SSTR-targeting radiopharmaceuticals such as [^68^Ga]Ga-DOTA-TATE, [^64^Cu]Cu-DOTA-TATE, [^111^In]In-DTPA-Octreotide, and [^68^Ga]Ga-DOTA-TOC.^55^ [^68^Ga]Ga-DOTA-TOC is the first FDA-approved ^68^Ga-labelled radiopharmaceutical for PET Imaging. [^177^Lu]Lu-DOTA-TATE, which binds strongly to SSTRs, was the first therapeutic radiopharmaceutical for NETs. It was first approved by the European Medicines Agency(EMA) in 2017. One year later, it was approved by the FDA. Both [^68^Ga]Ga-DOTA-TATE and [^68^Ga]Ga-DOTA-TOC can form a diagnostic and therapeutic pair with [^177^Lu]Lu-DOTA-TATE for patients with tumour imaging before specific treatment.^56^

#### Radiopharmaceuticals for neurology imaging

Alzheimer’s disease (AD) and Parkinson’s disease (PD) are neurodegenerative disorders associated with high morbidity in the elderly population. To date, 11 radiopharmaceuticals have been approved to diagnose the above two diseases, including [^123^I]FP-CIT/[^99m^Tc]TcHMPAO for SPECT scanning and [^18^F]flutemetamol/[^18^F]florbetapir for PET scanning. Presently, there are six ^18^F-fluorinated derivatives, three ^99m^Tc-labelled conjugates, and two ^123^I-iodinated small molecules ([^18^F]FDG is not included here).

To date, three ^18^F-fluorinated radiopharmaceuticals targeting β-amyloid (Aβ) for imaging AD progression have been approved by the FDA and used in clinical practice: [^18^F]florbetapir (Amyvid), [^18^F]flutemetamol (Vizamyl) and [^18^F]florbetaben (Neuraceq). These radiopharmaceuticals are beneficial for imaging Aβ plaque aggregation and evaluating the therapeutic efficacy in AD patients. [^11^C]PiB is a widely used PET tracer for Aβ imaging. However, the radioactive decay half-life of carbon-11 (20 min) limits its application. Compared with [^11^C]PiB, ^18^F-fluorinated radiopharmaceuticals have a longer half-life (109.8 min) and a narrower dynamic range than [^11^C]PiB and are suitable for PET scans.^57,58^ [^18^F]Florbetapir (^18^F-AV45), which was approved by the FDA in 2012, is the first ^18^F-fluorinated β-amyloid imaging tracer in the context of AD and it was originally developed by Hank F. Kung from a series of styryl pyridines (azastilbenes) lead compounds.^59^ One year later, GE Healthcare developed [^18^F]flutemetamol. In a phase III clinical trial involving 176 AD patients, PET scanning performed with [^18^F]flutemetamol indicated that the novel radiopharmaceutical was effective for the in vivo detection of brain Aβ plaque density, with high sensitivity and specificity.^60,61^ [^18^F]Florbetaben is the third radiopharmaceutical discovered by Piramal Imaging SA and was approved in 2014. In preclinical and clinical studies, [^18^F]florbetaben exhibited a strong binding affinity for synthetic Aβ fibrils and AD brain homogenates at the nanomolar level. Notably, [^18^F]florbetaben was shown to have no binding affinity for tau- or α-synuclein deposits, indicating high specificity for Aβ plaques.^62^ In 2020, Eli Lilly announced that the FDA had approved [^18^F]flortaucipir (Tauvid) for brain PET/CT imaging to diagnose AD patients with cognitive impairment.^58,63^ [^18^F]Flortaucipir is the first and only approved diagnostic agent for imaging tau-neurofibrillary tangles (NFTs) in the brain. The FDA approved Tauvid on the basis of its ability to significantly improve diagnostic outcomes and prevent deterioration of the condition.^58^ [^18^F]FDOPA, which was approved in 2019, is an effective diagnostic agent for PD syndromes that targets aromatic L-amino acid decarboxylase. It is a ^18^F-fluorinated analogue derived from natural L-DOPA. [^18^F]FDOPA exhibits a strong ability to penetrate the cell membrane through large-type amino acid transporters (such as LAT-1 and LAT-2). Compared with [^18^F]FDG, the sensitivity of [^18^F]FDOPA for imaging brain tumours is better. In addition, [^18^F]FDOPA is able to detect low-grade brain tumours and evaluate recurrent tumours in contrast with [^18^F]FDG. Therefore, [^18^F]FDOPA shows promising potential in brain tumour imaging.^64^

#### Radiopharmaceuticals for cardiology imaging and other pathologies

Radiopharmaceuticals have been increasingly used by cardiologists for the diagnosis of infection, inflammation, heart failure, etc. SPECT and PET are noninvasive methods that provide safe, rapid, and convenient options for patients.^65^ Based on the analytical results, eight radiopharmaceuticals have been approved worldwide, covering five main radionuclides, including technetium-99m (4), iodine-125 (1), and thallium-201 (1) for SPECT scanning and nitrogen-13 (1) and rubidium-82 (1) for PET scanning. Most cardiovascular imaging tracers are based on the mechanism of myocardial perfusion/myocardial blood flow and are freely diffusible, metabolically inert tracers, such as [^13^N]NH_3_· H_2_O and [^82^Rb]Rubidium Chloride.

In addition to the three diagnostic applications mentioned above, radiopharmaceuticals are widely used to diagnose bone diseases, nephrotic syndrome, hepatopulmonary syndrome, and hepatobiliary disorders. In these fields, the majority of radiopharmaceuticals are ^99m^Tc-labelled conjugates used for SPECT scanning, except for [^18^F]NaF injection, which is used for PET scanning. [^18^F]NaF was approved by the FDA in 1972 as a bone imaging tracer, and it can improve the accuracy of diagnosing malignant tumour bone metastases.^66,67^ Moreover, [^18^F]NaF is clinically significant for disease staging, determination of the therapeutic efficacy, and selection of appropriate clinical treatment.

### Radiopharmaceuticals for therapy

There are currently 13 therapeutic radiopharmaceuticals approved worldwide. All of them are used for the treatment of cancer. Radiopharmaceuticals are receiving increasing attention for cancer treatment because they emit either α-rays or β-rays thus destroying the DNA of targeted tumour cells. The mechanism of early-stage marketed radiopharmaceuticals for therapy, such as [^223^Ra]RaCl_2_, [^32^P]Sodium orthophosphate, and [^89^Sr]SrCl_2_, is based on organ accumulation but lacks tumour specificity, resulting in unsafety in vivo. To improve tumour uptake and retention, novel targeted radionuclide-conjugated small molecules, peptides, and antibodies have been well developed, such as [^131^I]MIBG, [^177^Lu]Lu-DOTA-TATE, and [^90^Y]Y-DTPA-Ibritumomab tiuxetan (Table 3).

**Table 3:** Radiopharmaceutical therapy agents approved wordwide

Radionuclides	Type	Agent	Targets	Indication
Radium-223	α	[^223^Ra]RaCl_2_	As a calcium mimic accumulating in bone^554^	Prostate cancer bone metastases
Phosphorus-32	β	[^32^P]Sodium orthophosphate	Major bone deposition^69^	Palliation of bone pain due to metastases
Strontium-89	β	[^89^Sr]SrCl_2_	As a calcium mimic accumulating in bone^69,70^	Prostate cancer bone metastases
Samarium-153	β	[^153^Sm]Lexidronam	As a calcium mimic accumulating in bone^71^	Relieving pain associated with bone metastasis
Lutetium-177	β	[^177^Lu]Lu-PSMA-617	PSMA	mCRPC
		[^177^Lu]Lu-DOTA-TATE	SSTR	NETs
Yttrium-90	β	[^90^Y]Resin microspheres	Selective internal radiation therapy	Hepatocellular carcinoma
		[^90^Y]Glass microspheres	Selective internal radiation therapy	Hepatocellular carcinoma
		[^90^Y]Y-DTPA-ibritumomab tiuxetan	CD20	Non-Hodgkin’s follicular lymphoma
Iodine-131	β	[^131^I]MIBG	Norepinephrine tansporter^73^	NETs
		[^131^I]Sodium iodinate	Sodium Iodide Symporter^555^	Hyperthyroidism and thyroid carcinoma
		[^131^I]Metuximab	CD147^556^	Hepatocellular carcinoma
		[^131^I]Tositumomab	CD20	Non-Hodgkin’s follicular lymphoma

#### Introduction and mechanism of therapeutic radiopharmaceuticals

##### Radium-223

[^223^Ra]RaCl_2_, which was approved by the FDA for treating bone metastases from prostate cancer in 2013, is the first and only approved radiopharmaceutical that emits α-radiation. The natural characteristics of α-particles (such as their short range and high energy) can cause irreversible damage to the DNA of cancer cells. Therefore, the development of novel α-particle-targeted therapies is currently a prevalent research topic.^68^ Except for [^223^Ra]RaCl_2_, the other therapeutic radiopharmaceuticals are β-particle-emitting radiopharmaceuticals that induce reversible DNA double-strand breakage in cancer cells.

##### Phosphorus-32, Samarium-153, Strontium-89

In addition to [^223^Ra]RaCl_2_, another three radiopharmaceuticals used for treating bone metastases are [^32^P]Sodium orthophosphate, [^153^Sm]Lexidronam, and [^89^Sr]SrCl_2_. Like radium-223, these radiopharmaceuticals are calcium mimics that can be localized in the skeleton as a component of the hydroxyapatite crystal together with calcium and the hydroxyl moiety.^69–71^

##### Iodine-131

Iodine-131 is the earliest radionuclide used for cancer therapy. [^131^I]Sodium iodinate can be highly ingested and aggregated in the thyroid gland, where it can release high-energy particles through β-decays to kill cancer cells.^72^ [^131^I]MIBG is another ^131^I-iodinated small molecule that can be used for adult pheochromocytoma and childhood neuroblastoma.^73^

Additionally, ^131^I-iodinated antibodies, namely [^131^I]Metuximab and [^131^I]Tositumomab, have been used for treating hepatocellular carcinoma (HCC) and non-Hodgkin’s follicular lymphoma, respectively. Another radio-antibody therapy, namely [^131^I]omburtamab, targets the tumour antigen B7-H3 for treating CNS/soft meningeal metastases in pediatric patients with neuroblastoma.^74^ Unfortunately, it failed to qualify for priority review through the FDA’s Biologics License Application (BLA) because of insufficient evidence supporting the improvement of overall survival.

##### Yttrium-90

As radioparticle pharmaceuticals, [^90^Y]Resin microspheres/glass microspheres have been approved for treating HCC via selective internal radiation therapy (SIRT).^75^ SIRT is based on the characteristics of blood supply in HCC tissues in the liver, where the tumour enlarges and the arterial vasculature thickens, creating conditions for the interventionist to perform. By using superselective intubation, millions of radioactive [^90^Y]Resin microspheres/glass microspheres are injected into the artery to supply blood to the tumour, and a very high dose of radiation is delivered inside the tumour cells to treat liver cancer.^76^

[^90^Y]Y-DTPA-Ibritumomab tiuxetan (Zevalin), a ^90^Y-labelled anti-CD20 monoclonal antibody (mAb), is the first FDA-approved radioimmunotherapy drug used for treating patients with relapsed or persistent low-grade malignant non-Hodgkin’s follicular lymphoma.^77^ However, because of the inconvenience in clinical use and competition with nonradioactive therapeutic drugs, the sale of Zevalin has not flourished.

##### Lutetium-177

^177^Lu-labelled peptide-targeted radionuclide therapy has been successfully used in clinical trials since the approval of [^177^Lu]Lu-DOTA-TATE and [^177^Lu]Lu-PSMA-617 in 2018 and 2022, respectively. Both radiopharmaceuticals are developed by companies affiliated with Novartis. The PSMA-targeting radiopharmaceutical [^177^Lu]Lu-PSMA-617 was approved for the treatment of mCRPC patients. This radiopharmaceutical is designed based on the glutamate-urea motif first reported by Alan P. Kozikowski and demonstrates promising potential for improving the survival of mCRPC patients.^78,79^ [^177^Lu]Lu-DOTA-TATE is an SSTR-targeting therapeutic agents used for treating patients with NETs. It is an analogue of somatostatin, an endogenous peptide that binds to SSTR2, which is overexpressed in cancer cells.^80,81^

### Limitation of the approved radiopharmaceuticals

As shown in Fig. 3, a variety of targeting ligands and radionuclides are applied for PET/CT and SPECT/CT imaging and tumour therapy. Despite the great progress in radiopharmaceutical discovery, limitations with respect to effectiveness and biosafety still exist. The major challenge for radiolabeled imaging agents is their nonspecific uptake in normal lesions. The off-target or on-target off-lesion binding hampers accurate clinical diagnosis, including the difficulty of distinguishing neuroinflammation and neurodegenerative disorder-related regions, benign prostatitis, and prostate cancers. Moreover, patients with low expression of certain targets (such as PSMA) are likely faced with pitfall detection. In addition, diagnostic agents with weak binding capacity are insufficient to detect low-expression tumours. As a result, both false-positive and false-negative diagnostic modalities should be optimized to improve the efficiency of radiopharmaceutical-based diagnosis. Therapeutic agents can prolong the lifetime of advanced-stage cancer patients. However, systemic toxicity, such as bone marrow compromise, xerostomia, and renal damage, is observed in clinical practice and may cause severe side effects. Other adverse effects, including resistance and the possibility of relapse, also need to be resolved. Overall, on the basis of approved radiopharmaceuticals, there is still an enormous scope for the application of radionuclides in various fields. In addition, approved radiopharmaceuticals can guide and provide appropriate directions for researchers to accelerate novel drug discovery.

## Emerging drug targets for radiopharmaceuticals

**Fig. 4:**
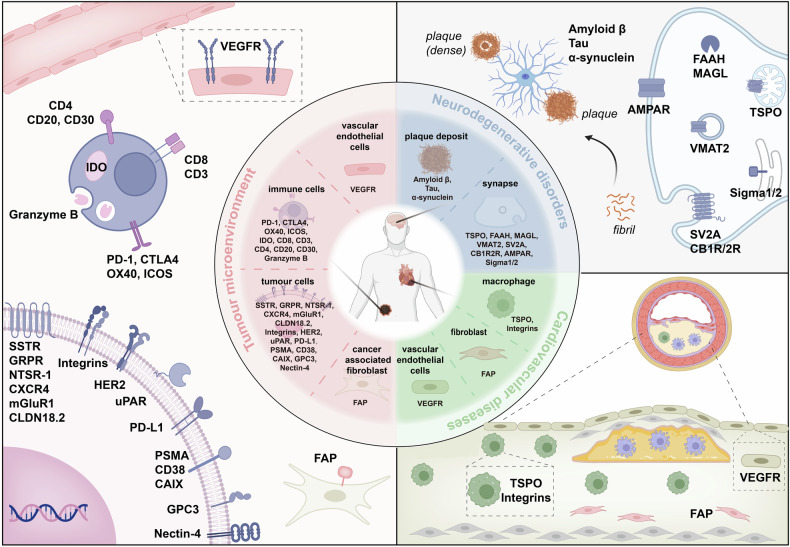
Widely studied targets for radiopharmaceuticals in tumour, neurodegenerative disorders and cardiovascular diseases. Radiopharmaceuticals are mainly used in the diagnosis and treatment of tumours, neurodegenerative disorders, and cardiovascular diseases. The TME contains tumour cells, immune cells, CAFs, and vascular endothelial cells, which play essential roles in cancer progression. Tumour targets for radiopharmaceutical development include GPCR-based transmembrane proteins (SSTR, GRPR, NTSR-1, CXCR4, and mGluR1), transmembrane proteins with four-pass domains (CLDN18.2), heterodimeric receptors (HER2 and the integrin family), other receptor (uPAR with no transmembrane and intracellular domains), immune checkpoints (PD-L1), and tumour antigens or other kinds of tumour biomarkers (PSMA, CD38, CAIX, GPC3, and Nectin-4). FAPs are expressed on both CAFs and tumour cells. VEGFRs are crucial tumour targets expressed by vascular endothelial cells. Immune cells that express checkpoints (PD-1, CTLA4, OX40, and ICOS), antigens (CD8, CD3, CD4, CD20, and CD30), and other biomarkers (IDO and Granzyme B) also serve as critical targets for cancer radiotheranostics. Aβ, tau, and α-synuclein plaques are the main causes of neurodegenerative disorders. The critical proteins expressed on synapses involved in neurotransmitter regulation include AMPAR and VMAT2 (transporter); FAAH and MAGL (signalling); SV2A, CB1R/21 R, sigma-1/2, and TSPO (transmembrane proteins), which have emerged as attractive targets for neurodegenerative disorders. Radiopharmaceuticals are currently used for the diagnosis of cardiovascular diseases. Owing to the important role of macrophages in disease progression, biomarkers that are expressed mainly on macrophages (TSPO, integrins) are potent imaging markers for cardiovascular pathology. Moreover, the FAP and VEGFR also showed potential in cardiovascular imaging. Part of this figure was created with Biorender.com

This chapter provides an in-depth analysis of potential radiopharmaceuticals in clinical and preclinical studies, classified by different disease targets. We focus mainly on introducing targeted radiopharmaceuticals that have been evaluated in clinical trials or first-in-human studies (Table 4). The emerging disease targets related to radiopharmaceuticals that are potential to achieve clinical translation are also briefly introduced, although only preclinical studies are available. This chapter will cover an overview of the characteristics and functions of potential targets, the development of targeted radiopharmaceuticals, and their applications in clinical and preclinical studies. Furthermore, this study addresses current limitations and offers insights into the future direction of these potential radiopharmaceuticals **(**Fig. 4**)**.

**Table 4:** Emerging targets involved in radiopharmaceutical development

Target	Agent	Agent Type	Radionuclide	Subjects	Major finding	Ref.
**FAP**	[^68^Ga]Ga-FAPI-04	Small Molecule	Gallium-68	Various tumour types	[^68^Ga]Ga-FAPI-04 was superior to [^18^F]FDG in terms of sensitivity and accuracy in detecting primary and metastatic lesions (SUVmax for some metastases lesions: 7-29.9).	^86–88^
	[^68^Ga]Ga-FAPI-46	Small Molecule	Gallium-68	BC	[^68^Ga]Ga-FAPI-46 exhibited a long retention time and low normal tissue uptake (Mean SUVmax: 13.4 early vs.14.3 late, SUVmax at 1 h: 1.7-24.0).	^89,90^
	[^18^F]AlF-FAPI-74	Small Molecule	Fluorine-18	Gastric cancer; Liver cancer; Pancreatic cancer	[^18^F]AlF-FAPI-74 was superior to [^18^F]FDG in imaging primary tumour, local recurrence, lymph node involvement, and bone metastases of cancers. It detected more peritoneal metastases than [^18^F]FDG (100% vs. 64%).	^91^
	[^68^Ga]Ga/[^18^F]AlF/[^177^Lu]/Lu-FAP-2286	Peptide	Gallium-68 /Fluorine-18/Lutetium-177	Solid tumours	[^68^Ga]Ga-FAP-2286 with superior binding affinity and prolonged tumour retention might be a preferred alternative to [^18^F]FDG in cancers with low to moderate [^18^F]FDG uptake (Median SUVmax of [^68^Ga]Ga-FAP-2286 and [^18^F]FDG in primary tumours: 11.1 vs. 6.9).	^99,100,102^
**PSMA**	[^68^Ga]Ga-PSMA-11	Small Molecule	Gallium-68	PC; mCRPC	Median SUVmax in the primary dominant intraprostatic tumours of [^18^F]PSMA-1007 vs. [^68^Ga]Ga-PSMA-11: 8.73 vs. 6.94.	^107^
	[^18^F]PSMA-1007	Small Molecule	Fluorine-18	PC	Enabling detection of low-grade lesions, suitable half-life, and high liver uptake.	^107^
	[^177^Lu]Lu-PSMA-617	Small Molecule	Lutetium-177	mCRPC	PSA progression-free survival: 7.6 months. Median OS: 13.5 months.	^49,109^
	[^68^Ga]Ga/[^177^Lu]Lu-PSMA-I&T	Small Molecule	Gallium-68/ Lutetium-177	Metastatic PC	[^68^Ga]Ga/[^177^Lu]Lu-PSMA-I&T was valuable for both diagnosis and treatment (SUVmax of the primary prostate tumour: 65.1; Median PSA progression-free survival and OS were 16.0 weeks and 13.8 months).	^111,112^
	[^18^F]/[^177^Lu]Lu-rhPSMA-7.3	Small Molecule	Fluorine-18/ Lutetium-177	PC	[^18^F]/[^177^Lu]Lu-rhPSMA-7.3 realized the radiotheranostics through the exchange of fluorine-19/fluorine-18 isotopes and lutetium-177/(nat) lutetium for radiolabelling (Tumour lesions received mean absorbed doses of [^177^Lu]Lu-rhPSMA-7.3 of 6.44 ± 6.44 Gy/GBq).	^114^
	[^99m^Tc]Tc-MIP-1404	Small Molecule	Technetium-99m	PC	[^99m^Tc]Tc-MIP-1404 had high diagnostic accuracy, low interobserver variability, and could monitor lymph node and bone metastases (SUVmax: 1.0 to 47.8), but had more hepatic fluid accumulation.	^108^
**SSTR**	[^68^Ga]Ga/[^177^Lu]Lu/[^64^Cu]Cu-DOTA-TATE	Peptide	Gallium-68/ Copper-64/ Lutetium-177	NETs	A significantly high sensitivity and a long progression-free survival. The SUVmax of [^68^Ga]Ga-DOTA-TATE was 20.4 ± 14.7.	^122^
	[^68^Ga]Ga/[^177^Lu]Lu/-LM3	Peptide	Gallium-68/ Lutetium-177	NETs	LM3 is an antagonist with excellent imaging properties, high sensitivity and accuracy (SUVmax of [^68^Ga]Ga-DOTA-LM3 and [^68^Ga]Ga-NODAGA-LM3: 38.9 ± 32.1 and 57.4 ± 38.5).	^132^
	[^68^Ga]Ga/[^177^Lu]Lu-JR11	Peptide	Gallium-68/ Lutetium-177	NETs	JR11 is an antagonist, advantageous imaging in the patient’s digestive system, holding efficacious and safe for treatment (SUVmax of [^68^Ga]Ga-DOTA-JR11 and [^68^Ga]Ga-NODAGA-JR11: 28.9 ± 26.1 and 39.7 ± 26.5).	^131–135^
**Integrin**	[^18^F]FPPRGD2	Peptide	Fluorine-18	Cervical cancer; Ovarian cancer	[^18^F]FPPRGD2 detects small metastases better than [^18^F]FDG with high accuracy (SUVmax of 0.8-5.8).	^140^
	[^18^F]Alfatide II	Peptide	Fluorine-18	BC	The specificity, accuracy, positive predictive value, and negative predictive value were higher for [^18^F]Alfatide II than for [^18^F]FDG.	^141^
	[^99m^Tc]Tc-3PRGD2	Peptide	Technetium-99m	Lung cancer	Good image quality and the TBR: 1.65 ± 0.47 for the planar scan and 2.78 ± 1.52 for SPECT imaging in most lung malignancies.	^142^
	[^18^F]BP	Peptide	Fluorine-18	Metastatic carcinoma	SUVmax of the primary lung lesion and right iliac wing metastasis was 5.2 and 13.5.	^146^
	[^68^Ga]Ga/[^177^Lu]Lu-DOTA-5G	Peptide	Lutetium-177	Pancreatic cancer	The safety evaluation of the first-in-human study demonstrated that the mean absorbed dose to the kidneys and bone marrow are 3.7 and 0.01 Gy, 6.93 and 0.02 Gy, 10.03 and 0.03 Gy, and 14.56 and 0.04 Gy, respectively, for dose level 1 to level 4.	^147^
	[^68^Ga]Ga-Trivehexin	Small Molecule	Gallium-68	Head and neck cancer; Pancreatic cancer;	High specificity, high and sustained uptake in primary lesions and liver metastases (SUVmax: 10–13 in metastatic PDAC).	^149^
**CXCR4**	[^68^Ga]Ga-Pentixafor	Peptide	Gallium-68	Solid tumour; Advanced MM;	High uptake in CXCR4-positive primary and metastatic lesions, with no significant uptake difference and higher detection rate than [^18^F]FDG (SUVmax: 2.13–37.91 in all tumour entities).	^152–154^
	[^64^Cu]Cu/[^177^Lu]Lu/[^90^Y]Y-Pentixather	Peptide	Copper-64/ Lutetium-177/ Yttrium-90	MM	One patient exhibited a reduction of SUVmax by greater than 35% lesions following [^177^Lu]Lu-pentixather treatment, while another patient demonstrated visual resolution of all previous [^18^F]FDG-positive lesions after [^90^Y]Y-pentixather treatment.	^157,158^
**GRPR**	[^68^Ga]Ga-Aca-BBN(7-14)	Peptide	Gallium-68	Optic pathway gliomas	Low metabolic stability. SUVmax and SUVmean of [^68^Ga]-NOTA-Aca-BBN(7–14) vs. [^18^F]FDG: 28.4 ± 5.59 vs. 0.47 ± 0.11 and 18.3 ± 4.99 vs. 0.35 ± 0.07.	^161,162^
	[^111^In]In/[^177^Lu]Lu-RM2	Peptide	Indium-111/ Lutetium-177	PC	Pancreas was the organ with the highest SUVmax measurements (average SUVmax: 64.91). The mean dose for tumour lesions was 6.20 ± 3.00 Gy/GBq.	^164–166^
	[^177^Lu]Lu-AMTG	Peptide	Lutetium-177	PC	Improved GRPR affinity and in vivo stability (Tumour uptake: 11.45 ± 0.43%ID/g).	^161,162^
	[^68^Ga]Ga/[^177^Lu]Lu/[^111^In]In-NeoBOMB1	Peptide	Gallium-68/ Lutetium-177/ Indium-111	Oligometastatic gastrointestinal mesenchymal stromal tumours	The SUVmax of [^68^Ga]Ga-NeoBOMB1 was 4.3-25.9 at 2 h post-injection. The highest uptake organ was pancreas (0.274 ± 0.099 mSv/MBq). The mean effective dose was 0.029 ± 0.06 mSv/MBq.	^170,171^
**UPAR**	[^64^Cu]Cu/[^68^Ga]Ga/[^177^Lu]Lu-AE105	Peptide	Copper-64/ Gallium-68/ Lutetium-177	BC; PC; Bladder cancer	The uPAR-positive lesions were observed in 68% (*n* = 65) of all patients and in 75% (*n* = 18) of patients with high-grade (grade 3 NENs) using [^68^Ga]Ga-NOTA-AE105 PET/CT imaging. [^177^Lu]Lu-AE105 significantly reduced the number of metastatic lesions.	^199–203^
**NTSR-1**	[^111^In]In/[^177^Lu]Lu-3BP-227	Small Molecule	Indium-111/Lutetium-177	Metastatic pancreatic adenocarcinoma	The kidneys are the dose-limiting organ and the most severe adverse event was reversible grade 2 anaemia.	^213,214^
**Nectin-4**	[^68^Ga]Ga-N188	Peptide	Gallium-68	Urothelial carcinoma,	The SUVmax of the bone metastatic lesions was 13.4 with highest contrast, which was higher than that of [^18^F]FDG (SUVmax: 6.2). The tumour uptake in patients with nectin-4(++), nectin-4(+) and nectin-4(-) was 8.3 ± 2.4, 3.7 ± 1.1, and 1.9 ± 0.4, respectively.	^245,246^
**CD8**	[^89^Zr]Zr-DFO-REGN5054	Antibody	Zirconium-89	Solid malignancies	[^89^Zr]Zr-DFO-REGN5054 PET/CT study in patients receiving cemiplimab uncovered key tumour features related to immune system effectiveness (NCT05259709).	^273^
	[^89^Zr]Zr-DFO-IAB22M2C	Antibody	Zirconium-89	Solid malignancies	The first-in-human study showed early imaging of lesions in patients under immunotherapy, suggesting this may be informative for CD8 + T-cell accumulation within tumours (SUV ranging from 5.85 to 22.8 in 6 target lesions).	^276^
	[^89^Zr]Zr-DFO-ZED88082A	Antibody	Zirconium-89	Solid malignancies	CD8^+^ T-cell distribution before and after ICB therapy was detected using [^89^Zr]Zr-DFO-ZED88082A PET/CT imaging in Phase II trials (SUVmax of 5.2 per patient). This tracer could characterize the complex dynamics of CD8^ +^ T cells in the context of ICBs and may inform immunotherapy.	^277^
**CD3**	[^99m^Tc]Tc-anti-CD3	Antibody	Technetium-99m	Various rheumatic diseases	[^99m^Tc]Tc-anti-CD3 was used to evaluate patients with RA and JIA, and there was a noticeable uptake in the joint.	^283^
**CD20**	[^90^Y]Y-DTPA- Ibritumomab tiuxetan	Antibody	Yttriium-90	Follicular lymphoma; NHL	Approved in 2002, it provided a new therapeutic avenue for relapsed and refractory low-grade or follicular B-cell NHL patients.	^294,295^
	[^131^I]-DFO-Tositumomab	Antibody	Iodine-131	NHL	Approved in 2003, it was designated for treating relapsed or refractory NHL patients.	^297,332,557^
**PD-1**	[^89^Zr]Zr- DFO-Pembrolizumab	Antibody	Zirconium-89	NSCLC; Melanoma	The uptake of [^89^Zr]Zr-DFO-Pembrolizumab in patients with metastatic melanoma or NSCLC correlated with response to anti-PD-1 treatment (SUVmax values of 4.9 in melanoma and 6.5 in NSCLC). The uptake in tumour lesions correlated with treatment response and patient survival.	^312^
**PD-L1**	[^18^F]-BMS-986192	Protein	Fluorine-18	Head and neck neoplasms; Lung cancer	It was in Phase I clinical trials for the diagnosis of lung cancer, head and neck cancer and solid tumours (EUDRACT 2015-004760-11).	^321^
	[^89^Zr]Zr-DFO-Durvalumab	Antibody	Zirconium-89	NSCLC	[^89^Zr]Zr-DFO-Durvalumab in stage III NSCLC patients undergoing chemoradiotherapy, could characterize PD-L1 expression.	^322^
	[^89^Zr]Zr-DFO-Atezolizumab	Antibody	Zirconium-89	Renal cell carcinoma	This was used for PD-L1 detection in renal cell carcinoma and was in Phase II clinical (NCT04006522).	^323^
	[^68^Ga]Ga-NOTA-WL12	Peptides	Gallium-68	NSCLC	The first-in-human study in NSCLC patient demonstrated the heterogeneity expression of tumour PD-L1 (SUVmax: 4.87%ID/g in patients with high PD-L1 expression).	^326^
	[^18^F]AlF-NOTA-NF12	Peptides	Fluorine-18	NSCLC; Oesophageal cancer;	PD-L1 could be detected by [^18^F]AlF-NOTA-NF12 in NSCLC patients (SUVmax: 3.29%ID/g).	^327^
**IDO**	α-[^11^C]-methyl-L-tryptophan	Small Molecule	Carbon-11	Lung or mediastinal tumour; BC (stage II-IV);	High IDO activity could be identified by the [^11^C]AMT PET/CT scans. IDO-positive breast cancers exhibited rapid [^11^C]AMT uptake within 20 min post-injection.	^331,332^
**Granzyme B**	[^68^Ga]Ga-NOTA-GZP	Peptide	Gallium-68	Lymphoma	Phase I study (NCT 04169321). First-in-human investigation was proved its safety as a imaging agent in subjects with cancer under ICB treatment.	^341^
	[^64^Cu]GRIP B	Peptide	Copper-64	PC; Renal cancer; Urethral cancer	Phase I/II study (NCT05888532). In vivo study of [^64^Cu]GRIP B was under investigated to evaluate its safety for granzyme B imaging in patients with advanced cancers.	^340^
**Aβ**	[^18^F]AZD4694	Small Molecule	Fluorine-18	Aging and dementia disease	Phase III investigation (NCT01886820360). This tracer was conformed with high cortical affinity in AD patients.	^355^
	[^18^F]FACT	Small Molecule	Fluorine-18	AD	[^18^F]FACT could clearly distinguish AD groups from healthy individuals, but the distribution and the PK in the brains were different.	^356^
	[^18^F]FDDNP	Small Molecule	Fluorine-18	AD	[^18^F]FDDNP could identify AD patients from healthy individuals in the elderly and showed more sensitivity to cognitive decline compared to [^18^F]FDG.	^357^
	[^18^F]FIBT	Small Molecule	Fluorine-18	AD	This tracer showed high affinity and specificity to Aβ, but not tau and α-synuclein, and demonstrated suitable uptake in human brains.	^358^
	[^123^I]IMPY	Small Molecule	Iodine-123	AD	[^123^I]IMPY was a safe radiopharmaceutical with ideal biokinetics for detecting Aβ in patients with AD.	^360^
	[^18^F]DRKXH1	Small Molecule	Fluorine-18	Healthy humans ; AD	The distribution volume ratio of [^18^F]DRKXH1 was higher than the approved [^18^F]AV45.	^362^
	[^11^C]BF-227	Small Molecule	Carbon-11	Healthy humans ; AD	[^11^C]BF-227 had a strong affinity for synthetic Ab1-42 fibrils.	^363^
	[^11^C]AZD2184	Small Molecule	Carbon-11	AD	[^11^C]AZD2184 could also detect AD patients, but the undesired binding with the white matter limited its further use.	^364^
	[^11^C]SB-13	Small Molecule	Carbon-11	AD	[^11^C]SB-13 showed a comparable effect in vivo with [^11^C]PIB when diagnosing AD subjects from healthy individuals.	^365^
**Tau**	[^18^F]SNFT-1	Small Molecule	Fluorine-18	AD (sections)	[^18^F]SNFT-1 had a high affinity for tau aggregates in AD brains. It had the potential to be a selective tau targeting radiotracer candidate.	^368^
	[^18^F]PM-PBB3	Small Molecule	Fluorine-18	Frontotemporal dementia	The PET/CT imaging of [^18^F]PM-PBB3 revealed a slight deposition of tau protein in the cerebral lobes.	^369^
	[^18^F]THK-5351	Small Molecule	Fluorine-18	AD; AGD	It had higher contrast and lower subcortical white matter retention compared with [^18^F]THK-5117. The number of reactive astrocytes in the postmortem brain was proportional to the uptake of [^18^F]THK-5351.	^371,372^
	[^18^F]NML	Small Molecule	Fluorine-18	AD; MCI; Healthy humans	Despite the high affinity of this tracer for tau protein aggregates in vitro, [^18^F]NML didn’t show good results in vivo with low brain retention in patients with MCI and AD.	^375^
	[^18^F]PI-2620	Small Molecule	Fluorine-18	PSP; AD	Phase III investigation (NCT05641688365). Patients with PSP could be identified by [^18^F]PI-2620 imaging with 68% accuracy compared to 80% in the healthy individuals. In AD patients, asymmetric uptake was significantly higher than in the healthy individuals.	^360,376,377^
**TSPO**	[^11^C] PK11195	Small Molecule	Carbon-11	Multiple degenerative diseases;	High correlation of increasing binding of [^11^C]PK11195 to amyloid deposit, inflammatory lesions in MCI patients, and reduced glucose metabolism in patients with neurodegenerative diseases.	^380^
	[^11^C]PBR28	Small Molecule	Carbon-11	Alcohol use disorders; Stroke;	Exhibiting a positive correlation between neuroimmune response and degree of alcohol intoxication. Increased uptake in the infarcted area in the brain of stroke patients.	^383,384^
	[^18^F]FEPPA	Small Molecule	Fluorine-18	AD; OCD;	Increased binding in the white and grey matter of AD patients, and the cortico-striatal-thalamic circuit involving the orbitofrontal cortex of OCD patients.	^385^
	[^11^C]DPA-713	Small Molecule	Carbon-11	AD; PD; Epilepsy;	Examination of microglial activation in AD and PD patients. The uptake confirmed the association between epilepsy and cognitive dysfunction pathologies with neuroinflammation.	^386,387^
	[^18^F]DPA-714	Small Molecule	Fluorine-18	Stroke; ALE; Epilepsy;	This tracer was in Phase II study (NCT03457493). Exhibiting a positive correlation between its uptake and multiple neurodegenerative disorders. Potentially to detect the microglia activation and reactive gliosis-related innate immunity of patients with CD8 + T-cell meditated ALE.	^388–391^
	[^18^F]PBR111	Small Molecule	Fluorine-18	MS	High specific uptake in human brains varies in genetic variation and age. Confirming the feasibility of [^18^F]PBR111 to characterize the immune responses of MS patients.	^392,393^
	[^18^F]GE180	Small Molecule	Fluorine-18	AD; MS; glioma;	High binding capacity to human TSPO and insensitive to *rs6971* polymorphism. Ideal in vivo behaviour in relapsing-remitting MS and AD. [^18^F]GE180 could be used to prodict recurrent glioma patients.	^395–397^
**α-synuclein**	[^18^F]ACI-12589	Small Molecule	Fluorine-18	PD; MSA;	High binding affinity to pathological α-synuclein in different tissues of both PD and MSA patients. Significant accumulation in MSA patients, but limited binding in PD patients.	^411,412^
	[^18^F]-F0502B	Small Molecule	Fluorine-18	PD	Unique selectivity for α-synuclein but not binding tau or Aβ.	^413^
**Sigma receptors**	[^18^F]ISO-1	Small Molecule	Fluorine-18	Lymphoma; BC; Head and neck cancer	Researchers evaluated the safety and dosimetry of [^18^F]ISO-1 in patients, showing that the doses to normal organs allow for the safe administration of up to 550 MBq.	^420^
**CB1R/CB2R**	[^11^C]OMAR	Small Molecule	Carbon-11	Healthy humans	PET/CT imaging of [^11^C]OMAR demonstrated that a declined CB1R availability with increasing age, also in men compared to women.	^422^
	[^11^C]MePPEP	Small Molecule	Carbon-11	Healthy humans	[^11^C]MePPEP had an acceptable uptake in the region of the human brain, which can be observed at least 210 min post-injection.	^423^
	[^11^C]NE40	Small Molecule	Carbon-11	Healthy humans	[^11^C]NE40 had a suitable quick brain kinetics in the healthy human brain.	^425^
**VMAT2**	[^11^C]DTBZ	Small Molecule	Carbon-11	AD; DLB;	A single scan of [^11^C]DTBZ offered as much diagnostic information as 2-scans of [^18^F]FDG.	^427^
	[^18^F]AV-133	Small Molecule	Fluorine-18	PD	Reduction accumulation in PD, and uncertain PD patients. Suitable for monitoring PD progression over long-term.	^430–432^
				Type 1 diabetes	Significant decreased pancreatic uptake in patients with type 1 diabetes.	^433–435^
**SV2A**	[^11^C]UCB-J	Small Molecule	Carbon-11	Lewy body dementia; PSP; AD; Frontotemporal dementia from C9orf72; HD; aMCI;	[^11^C]UCB-J was valuable to early diagnosis of synaptic loss-related neurodegenerative disorder and facilitates disease management.	^437,441–446^
	[^18^F]UCB-H	Small Molecule	Fluorine-18	BvFTD	[^18^F]UCB-H PET/CT iamging indicated loss of synaptic in the right anterior parahippocampal gyrus of bvFTD patients.	^448^
	[^18^F]SynVesT-1	Small Molecule	Fluorine-18	Healthy humans	Excellent PK with rapid brain uptake and high specific binding capacity in vivo.	^449,450^
	[^18^F]SynVesT-2	Small Molecule	Fluorine-18	Healthy humans	High specific uptake, low nonspecific uptake in the brain, and faster kinetics compared with [^18^F]SynVesT-1 and [^11^C]UCB-J	^451^
**MAGL**	[^18^F]T-401	Small Molecule	Fluorine-18	Healthy humans	The distribution volume values were in agreement with the MAGL expression pattern in human brains, which was mainly uptake by the cerebral cortex, modestly accumulated in the thalamus and putamen, and lowest in the brainstem and white matter.	^455^
**FAAH**	[^11^C]CURB	Small Molecule	Carbon-11	ASPD; Human cannabis addiction; Alcohol Psychiatric;	Lower density in the amygdala of ASPD, and negatively associated with assaultive aggression in the cerebellum and striatum of ASPD. [^11^C]CURB PET/CT imaging revealed that higher FAAH activity was associated with lower hippocampus volume and greater hippocampus glutamate glutamine in patients with alcohol psychiatric.	^471,473,474^
	[^11^C]MK-3168	Small Molecule	Carbon-11	Healthy volunteers;	Single doses of [^11^C]MK-3168 (10 mg) resulted in more than 95% occupancy.	^475^
**AMPAR**	[^11^C]K-2	Small Molecule	Carbon-11	MDD; BD; Psychiatric disorders;	SUV level in the cerebellum was higher in MDD than in BD, but in other aera of the brain was higher in BD.	^485,486^
	[^11^C]HMS011	Small Molecule	Carbon-11	Human brains	Quick brain uptake, rapid clearance, and notable individual variability.	^488^

^*^Abbreviations: *FAPI* Fibroblast activation protein inhibitor, *EC50* Half maximal effective concentration, *BC* Breast cancer, *PSMA* Prostate-specific membrane antigen, *FDG* Fluorodeoxyglucose, *PC* Prostate cancer, *mCRPC* Metastatic castration-resistant prostate cancer, *SSTR* Somatostatin receptor, *CXCR4* C-X-C chemokine receptor type 4, *GRPR* Gastrin-releasing peptide receptor, *uPAR* Urokinase-type PA receptor, *NTS-1* Neurotensin-1, *Nectin-4* Nectin cell adhesion molecule 4, *CD8* Cluster of differentiation of 8, *CD3* Cluster of differentiation of 3, *CD20* Cluster of differentiation of 20, *NHL* Non-Hodgkin’s lymphoma, *NSCLC* Non-small cell lung cancer, *PD-1* Programmed death 1, *PD-L1* Programmed death ligand 1, *IDO* Indoleamine 2,3-dioxygenase, *AD* Alzheimer’s disease, *AGD* Argyrophilic grain disease, *PSP* Progressive Supranuclear Palsy, *PD* Parkinson’s disease, *HD* Huntington’s disease, *MCI* Mildly cognitively impaired, *MSA* Multiple system atrophy, *CB1R/CB2R* Cannabinoid receptor 1/ cannabinoid receptor 2, *K*_*d*_ Dissociation constant, *DLB* Dementia with Lewy body, *TSPO* Translocator protein, *OCD* Obsessive compulsive disorder, *ALE* Autoimmune limbic encephalitis, *MS* Multiple sclerosis, *BvFTD* Behavioural variant of frontotemporal dementia, *SV2A* Synaptic vesicle glycoprotein 2A, *MAGL* Monoacylglycerol lipase, *FAAH* Fatty acid amide hydrolase, *ASPD* Antisocial personality disorder, *AMPA* α-Amino-3-Hydroxy-5-Methyl-4-Isoxazolepropionic Acid, *MDD* Major depressive disorder, *BD* Bipolar disorder

### Tumour-directed radiopharmaceutical targets

**Fig. 5:**
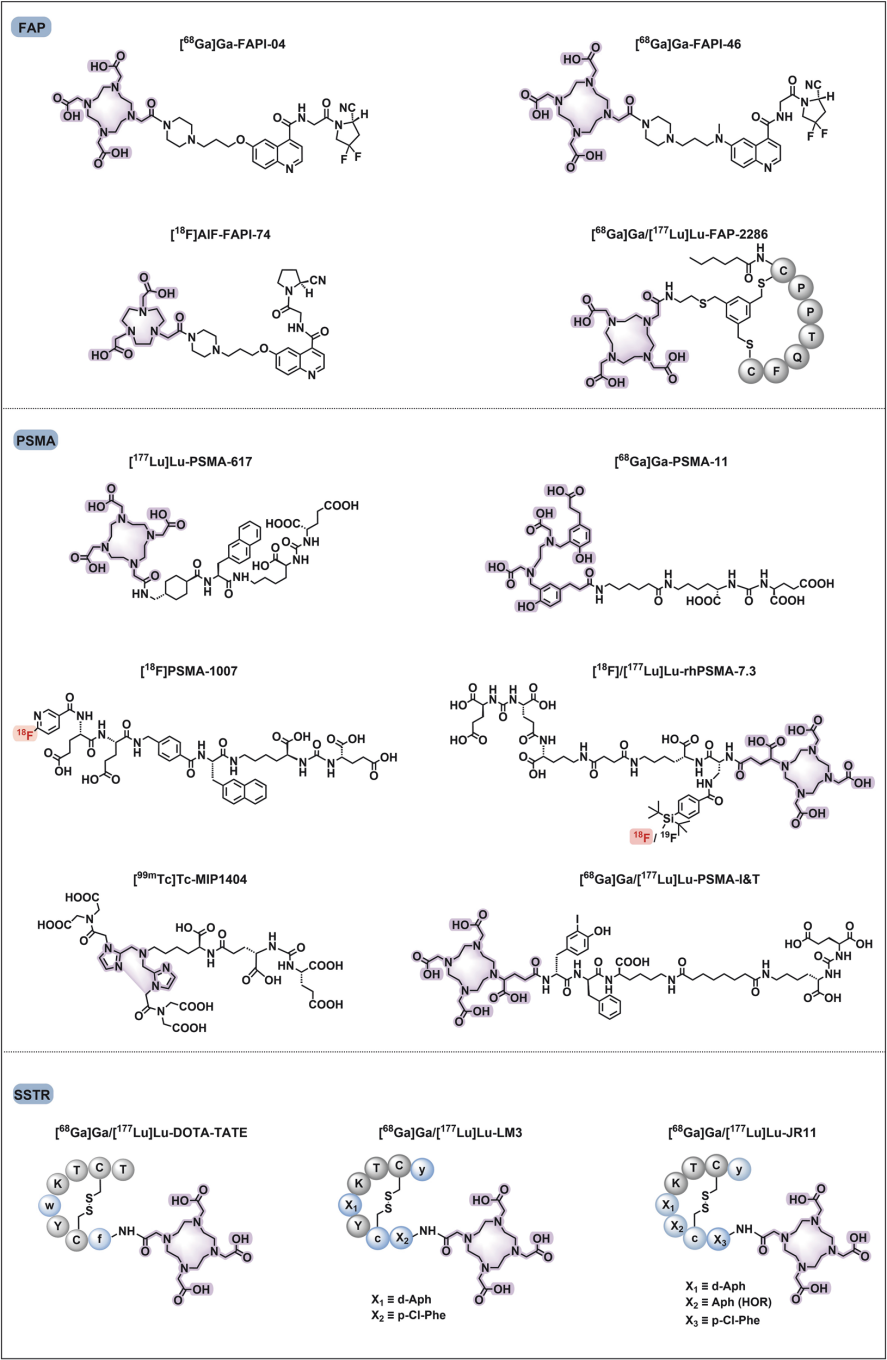
Chemical structures of clinically evaluated tumour-direct FAP, PSMA, and SSTR targeting radiopharmaceuticals. Representative clinically evaluated tumour-directed FAP-, PSMA- and SSTR-targeting radiopharmaceuticals. PSMA-targeting radiopharmaceuticals with a glutamate-urea-lysine structural motif, including PSMA-11, PSMA-1007, PSMA-617, and rhPSMA-7.3, have been approved. PSMA-targeting ligands that enable simultaneous diagnosis and therapy, including PSMA-I&T and rhPSMA, are of high value. SSTR-targeting radiopharmaceuticals play essential roles in the radiotheranostics of NETs. The antagonists, including LM3 and JR11, which have greater safety and affinity, are promising in SSTR-targeting imaging agents. FAP-targeting radiotracers may prove advantageous over [^18^F]FDG in the localization and visualization of solid tumours, such as FAPI-04, FAPI-46, and FAPI-74. Additionally, FAP-2286 has been shown to facilitate radiotheranostics. Grey circles: natural amino acids; blue circles: unnatural amino acids; highlighting in red: labelling with fluorine-18; highlighting in purple: chelators for metal radionuclide labelling

**Fig. 6:**
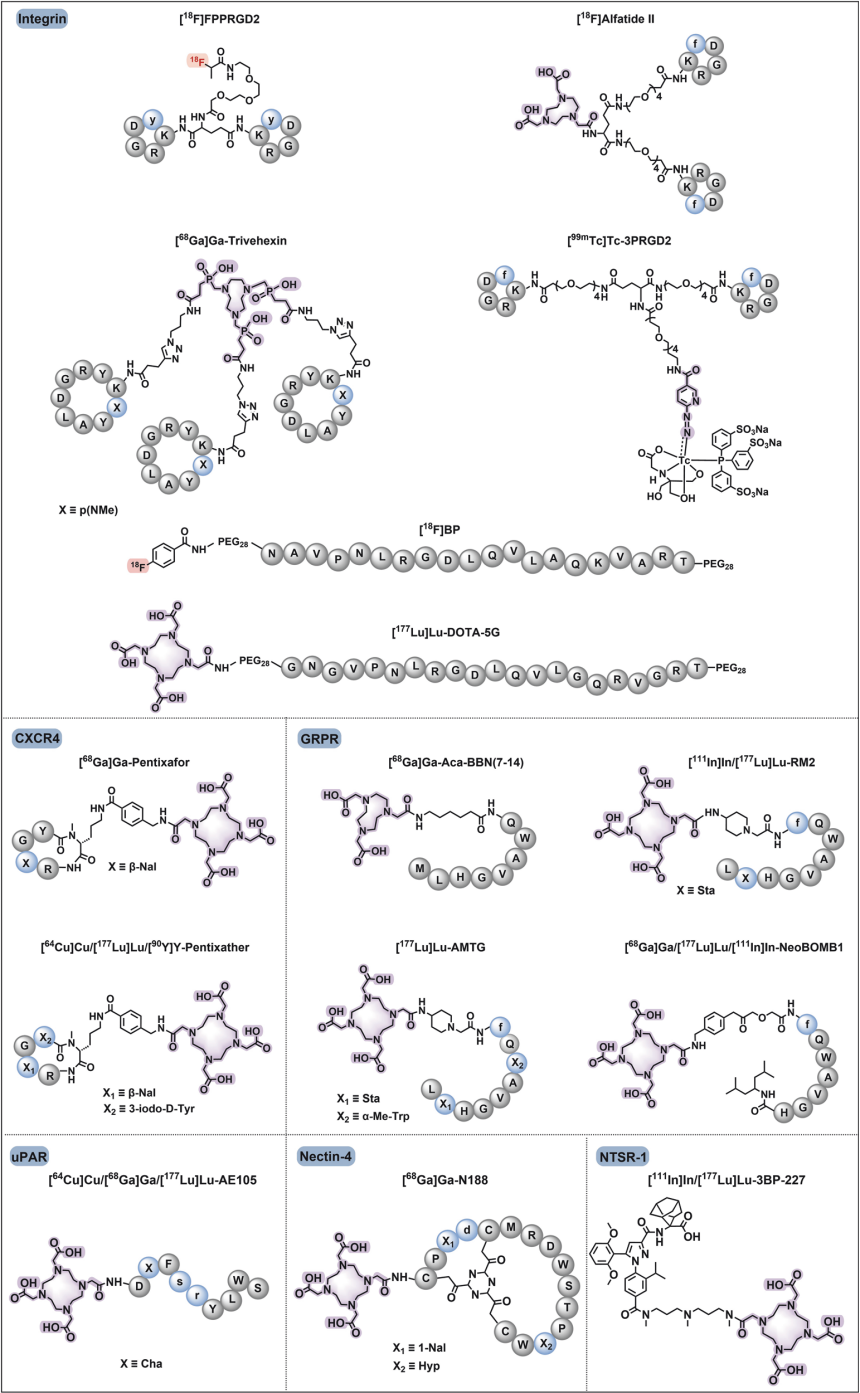
Chemical structures of clinically evaluated tumour-direct Integrin, CXCR4, GRPR, UPAR, NTSR-1, Nectin-4 targeting radiopharmaceuticals. Representative clinically evaluated tumour-directed promising radiopharmaceuticals targeting Integrin, CXCR-4, GRPR, uPAR, NTSR-1, and Nectin-4. In the Integrin family, RGD motif-based αvβ3-targeting radiopharmaceuticals, particularly [^99m^Tc]Tc-3PRGD2, may be the next widely implemented diagnostic agent in clinical applications. Integrin αvβ6 targeting ligand 5 G has also demonstrated promising results in clinical trials. The efficacy of CXCR4-targeting ligands, including pentixafor and pentixather, as well as uPAR-targeting ligand AE105, has been demonstrated in numerous clinical studies. GRPR antagonists based on the BBN-like peptides, such as BBN(7–14), RM2, AMTG, and NeoBOMB1, have demonstrated remarkable therapeutic efficacy in the RPT of GRPR-positive tumours. A non-peptide NTSR-1 antagonist, 3BP-227, has been demonstrated to exhibit great receptor affinity and diminished normal organ uptake, making it a promising NTSR-1-targeted radiopharmaceutical for clinical investigation and translation. The bicyclic-peptide-based radiotracer, [^68^Ga]Ga-N188, has been demonstrated to be efficient for imaging tumour Nectin-4. Grey circles: natural amino acids; blue circles: unnatural amino acids; highlighting in red: labelling with fluorine-18; highlighting in purple: chelators for metal radionuclide labelling

Tumour-targeted radiopharmaceuticals are emerging as promising clinical approaches that offer noninvasive, real-time diagnosis of tumour lesions and highly effective, safe treatments with strong antitumour efficacy.^82^ The identification of suitable targets facilitates successful clinical translation. In this section, we review the promising oncology targets and involved radiopharmaceuticals that exhibit significant clinical progress or remarkable cancer targeting capability. We will also discuss their potential applications, such as the use of tumour-specific targets in cardiovascular imaging, which is anticipated to reach clinical application in the future. Additionally, we summarize the pharmacological characteristics of these targets, and the current research and clinical progress on representative radiopharmaceuticals and provide an insight into their future development. We also present the chemical structures of clinically evaluated radiopharmaceuticals involving tumour-directed radiopharmaceutical targets (Figs. 5, 6**)**.

#### Fibroblast activation protein-α (FAP)

The tumour microenvironment (TME), which comprises tumour cells, cancer associated fibroblasts (CAFs), microvascular cells, immune cells and the extracellular matrix, plays a fundamental role in tumour progression, invasion and migration.^83^ FAP is a transmembranous subtype II serine protease. It is upregulated in 90% of CAFs in multiple cancers and is expressed at low levels in the fibroblasts of healthy adult tissues.^84^ The ability of FAP inhibitors (FAPIs) to target the TME is well-suited for the design and development of targeted radiopharmaceuticals.

FAP-targeting small molecule inhibitors have been studied in-depth in radiopharmaceutical clinical studies. Loktev et al. reported two FAPIs based on the quinoline structure: FAPI-01 for radioiodine labelling and its nonhalogenated derivative FAPI-02. In a patient with locally advanced lung adenocarcinoma, [^68^Ga]Ga-FAPI-02 showed greater metastatic lesion uptake and imaging contrast than did [^18^F]FDG. However, the short retention time of [^68^Ga]Ga-FAPI-02 limits its clinical applications.^85^ Subsequently, 13 novel FAPIs were designed for therapeutic applications by Lindner et al. Among these, FAPI-04 is considered the most valuable.^86^ On the basis of the PET/CT imaging results from 80 cancer patients, the tumour uptake of [^68^Ga]Ga-FAPI-04 is the most pronounced in patients with sarcoma, oesophageal cancer, breast cancer (BC), and cholangiocarcinoma (mean standardized uptake value [SUVmean]: 12 at 1 h after injection).^87^ Chen et al. performed a comprehensive comparative evaluation of [^68^Ga]Ga-FAPI-04 with [^18^F]FDG in 75 patients with different types of cancers and reported that [^68^Ga]Ga-FAPI-04 was more sensitive and accurate than [^18^F]FDG in detecting primary and metastatic lesions (detection rate of primary tumours: 98.2% vs. 82.1%; lymph nodes: 86.4% vs. 45.5%).^88^ To increase tumour uptake and prolong the retention time of FAPIs, Loktev et al. modified the structure of the quinoline molecule, the binding region between quinoline and the chelators and synthesized 15 new FAPIs.^89^ FAPI-46 has demonstrated superiority as a diagnostic agent in clinical imaging studies because of its low uptake in normal tissues. Ferdinandus et al. used [^68^Ga]Ga-FAPI-46 to detect 400 lesions in 69 patients and demonstrated its potential to differentiate between inflammatory and malignant uptake.^90^

Given the lower end-point positron energy and longer half-life, ^18^F-fluorinated FAPIs may be more appropriate for promoting the imaging modalities that benefit patients. [^18^F]FAPI-74 is a potential tracer that is absorbed by tumours and rapidly excreted by the kidneys. Xu et al. investigated the diagnostic performance of [^18^F]FAPI-74 in 112 patients with gastric, liver, and pancreatic cancer. Compared with [^18^F]FDG, [^18^F]FAPI-74 has advantages in detecting primary tumours, local recurrence, and bone and visceral metastases of cancer; however, in terms of specificity, [^18^F]FAPI-74 does not have a significant advantage over [^18^F]FDG.^91^

To meet the challenge of the limited retention time of radiolabelled FAPI within tumour cells, strategies aimed at modulating PK through increased binding ability with serum albumin have gained prominence. Two widely used albumin binders, namely, 4-(p-iodophenyl) butyric acid (IPBA) and truncated Evans blue (EB), have shown potential in enhancing the tumour accumulation and retention of radiopharmaceuticals, thereby reinforcing their therapeutic effects.^92^ Wen et al. developed a series of albumin-binding FAPIs based on FAPI-02, named EB-FAPI-B1 to B4. Among the four radiopharmaceuticals, [^177^Lu]Lu-EB-FAPI-B1 showed significant tumour uptake and prolonged retention time; moreover, it maintained high uptake even at 96 h post-injection.^93^ Fu et al. conducted a first-in-human and dose-escalation study of the EB-conjugated FAPI, [^177^Lu]Lu-EB-FAPI ([^177^Lu]Lu-LNC1004), in patients with metastatic radioiodine-refractory thyroid cancer. In these patients, the dose of 3.33 GBq per cycle was well tolerated, with encouraging therapeutic efficacy (objective response rate and disease control rate of 25% and 83%, respectively) and acceptable adverse effects.^94^ Meng et al. synthesized three albumin-binding FAPI ligands (FSDD_0_I, FSDD_1_I, and FSDD_3_I) derived from FAPI-04 by coupling IPBA with a bifunctional chelator. The authors showed that the binding affinity of these three FAPI ligands was not impaired after IPBA conjugation. PET/CT imaging demonstrated that [^68^Ga]Ga-FSDD_0_I had significant tumour uptake compared with [^68^Ga]Ga-FAPI-04. Notably, [^68^Ga]Ga-FSDD_0_I exhibited significantly greater tumour uptake and prolonged retention time than the other two tracers did, which might be attributed to its enhanced albumin-binding properties or relatively low hydrophilicity.^95^ In addition to conventional albumin binders, fatty acids, such as palmitic acid (C16), have been investigated as albumin-binding moieties. Zhang et al. conjugated lauric acid (C12) and C16 to FAPI-04 and reported that in comparative therapeutic assessments with [^177^Lu]Lu-FAPI-04, both [^177^Lu]Lu-FAPI-C12 and [^177^Lu]Lu-FAPI-C16 demonstrated superior therapeutic efficacy compared with [^177^Lu]Lu-FAPI-04; moreover, [^177^Lu]Lu-FAPI-C16 exhibited significantly prolonged tumour retention compared with [^177^Lu]Lu-FAPI-C12.^96^ Another effective method to resolve the issues of short tumour retention is to develop dimer derivatives. Zhao et al. synthesized a FAPI dimer, DOTA-2P(FAPI)2 based on FAPI-46. Clinical evaluations indicated that [^68^Ga]Ga-DOTA-2P(FAPI)2 exhibited prolonged tumour retention compared with the monomer, [^68^Ga]Ga-FAPI-46, with a sustained high concentration in the blood pool at four hours post-injection.^97^ In a recent retrospective study, Yadav et al. examined the clinical outcomes of FAPI dimer radionuclide therapy utilizing [^177^Lu]Lu-DOTAGA-FAPi in a cohort of 19 patients with metastatic BC. The promising clinical disease control rate was 95%, and the clinical objective response rate was 84%. Furthermore, no severe adverse effects, including haematological, renal, or hepatic toxicities, were observed during the study.^98^

Compared with the small-molecule FAPI series, cyclic peptide FAPIs have increased target selectivity, high binding affinity, and prolonged tumour retention time, thus demonstrating promising outcomes in clinical trials. In particular, 3B Pharmaceuticals reported a potential clinical candidate, FAP-2286 which was screened from 263 different FAP-targeting peptide structures. An initial clinical evaluation or recurrence detection in 64 patients with 15 cancer types revealed that the tumour uptake of [^68^Ga]Ga-FAP-2286 was much greater than that of [^18^F]FDG in primary tumours and lymph node metastases (median SUVmax: 11.1 and 10.6 vs. 6.9 and 6.2), and the primary tumour detection rate of [^68^Ga]Ga-FAP-2286 was significantly greater than that of [^18^F]FDG PET/CT (100% vs. 80.4%). Moreover, it could be considered the preferred alternative to [^18^F]FDG in cancers with low to moderate uptake.^99^ Recently, Liu et al. compared the performance of [^18^F]AlF-FAP-2286 with that of established radiopharmaceuticals in a preclinical study. [^18^F]AlF-FAP-2286 demonstrated superior imaging contrast with high target uptake and satisfactory retention in both mouse models and in cancer patients.^100^ Baum et al. reported the first-in-human results of [^177^Lu]Lu-FAP-2286 in 11 patients with advanced adenocarcinomas of the pancreas, breast, rectum, or ovary. The dosage of 5.8 ± 2.0 GBq was well tolerated, and the whole-body effective dose was 0.07 ± 0.02 Gy/GBq. The mean absorbed doses for the kidneys and red marrow were 1.0 ± 0.6 Gy/GBq and 0.05 ± 0.02 Gy/GBq, respectively. These findings suggest that [^177^Lu]Lu -FAP-2286 RPT is a promising approach for relatively few side effects.^101^ In 2022, Rao et al. reported that [^177^Lu]Lu-FAP-2286 was administered to a patient with systemic metastases from squamous cell carcinoma of the right lung. After 9 weeks of treatment with a single dose of 7.0 GBq, a significant decrease of tumour FAP expression in the patient was observed, as indicated by the PET/CT scans of [^68^Ga]Ga-FAP-2286. This encouraging finding highlights the importance of [^177^Lu]Lu-FAP-2286 as one of the most promising radiopharmaceuticals.^102^

Detection of FAP in myocardial tissue allows early identification of cardiac injury due to increased FAP expression in activated myocardial fibroblasts, and FAPI has been extensively explored clinically in cardiovascular diseases such as infarction, heart failure, tumour treatment-related cardiotoxicity, and cardiomyopathy.^103^ Zhang et al. performed [^68^Ga]Ga-FAPI-04 PET/MR on 26 patients with advanced cardiac infarction and reported that FAPI uptake was significantly greater in the left ventricular remodeling group. A high FAPI signal predicts adverse ventricular remodeling, and FAPI imaging is expected to become a new diagnostic approach for reflecting ventricular remodeling.^104^

Currently, the insufficient retention time of FAPIs in tumours does not meet the requirements of clinical practice. However, several strategies have been developed to overcome this limitation. Moreover, FAP-targeting cyclic peptide radiopharmaceuticals are more advantageous and have greater potential for cancer therapy. We believe that FAP-targeting radiopharmaceuticals will be the protagonists of the next radiopharmaceutical revolution and will gain immense popularity.

#### Prostate-specific membrane antigen (PSMA)

PSMA is a type II membrane glycoprotein first identified in prostate cancer cell lines. PSMA is overexpressed in more than 90% of malignant PCs, and its expression level increases significantly with the degree of malignancy. Moreover, the PSMA expression level in normal tissues is 100- to 1000-fold lower than that in PCs. The excellent biological properties of PSMA make it a key target for developing novel radiopharmaceuticals.^105^ Recently, PSMA-targeting radiopharmaceuticals have become the hallmark of RPT because of their superior clinical performance in the diagnosis and treatment of patients with advanced and metastatic CRPC.^106^

For diagnosis, [^18^F]PSMA-1007 and [^68^Ga]Ga-PSMA-11 were approved for the diagnosis of PCs, and a head-to-head comparative study demonstrated that [^18^F]PSMA-1007 and [^68^Ga]Ga-PSMA-11 could identify intermediate- or high-risk PCs. Notably, [^18^F]PSMA-1007 additionally detected low-grade lesions of limited clinical relevance and overcame several practical restrictions related to ^68^Ga-labelling PSMA-targeting tracers because of its longer half-life, excellent energetic properties, and non-urinary excretion properties.^107^ [^99m^Tc]Tc-MIP-1404 is another prospective PSMA-targeting SPECT radiopharmaceutical. Schmidkonz et al. analyzed 93 patients with histologically proven cancer who underwent [^99m^Tc]Tc-MIP-1404 SPECT/CT scans prior to therapy.^108^ The authors suggested that [^99m^Tc]Tc-MIP-1404 could detect lymph nodes and bone metastases in a subset of previously untreated patients with PC. However, it is worth noting that liver accumulation of [^99m^Tc]Tc-MIP-1404 is high, making it difficult to diagnose liver metastases. The successful clinical translation of diagnostic radiopharmaceuticals has promoted the pursuit of PSMA-targeting therapeutic radiopharmaceuticals; however, the in vivo therapeutic effectiveness of three abovementioned ligands is limited. The successful development of [^177^Lu]Lu-PSMA-617 and advancements in its clinical application demonstrated the therapeutic value of PSMA-targeting radiopharmaceuticals.^49,109^ Images acquired from [^68^Ga]Ga-PSMA-617 PET/CT between 2 and 3 h post-injection appeared to be optimal uptake and imaging contrast, however, this does not match well with the half-life of gallium-68. Therefore, the design of PSMA-targeting ligands that can meet both diagnostic and therapeutic requirements is an emerging trend in the development of PSMA-targeting radiopharmaceuticals.^110^ To address this, Weineisen et al. developed PSMA-I&T with DOTAGA as a chelator, which enabled rapid and high-yield radiolabelling with both gallium-68 and lutetium-177. [^68^Ga]Ga-PSMA I&T shows promise for high-quality PET/CT imaging of metastatic PCs, while its ^177^Lu-labelled counterpart has targeting and retention properties for endoradiotherapy.^111^ A clinical trial using [^177^Lu]Lu-PSMA-I&T in 56 patients with mCRPC revealed that 80.4% of patients presented a decrease in prostate-specific antigen (PSA) levels, whereas 58.9% of patients presented a greater than 50% reduction in pain severity. None of the patients reported clinically significant severe adverse events during hospitalization or at 28 months of follow-up.^112^

A novel series of radiohybrid (rh) PSMA-targeting ligands were recently developed by incorporating a silicon fluoride acceptor (SiFA) for fluorine-19/fluorine-18 isotope exchange radiolabelling and a chelator for complexation with a (radio)metal (lutetium-177, gallium-68, or actinium-225) and have shown promising prospects in clinical applications. The FDA-approved lead rhPSMA diagnostic radiopharmaceutical [^18^F]flotufolastat (^18^F-rhPSMA-7.3) demonstrated favourable biodistribution and diagnostic efficacy for N-staging and localization of biochemical relapse in patients with recently diagnosed and recurrent PCs.^113^ In a first validation of the radiohybrid technology for therapeutic applications, [^177^Lu]Lu-rhPSMA-7.3 demonstrated 2.8-fold and 4.7-fold increases in tumour uptake compared with [^177^Lu]Lu-PSMA I&T at 1 and 168 h after injection, respectively. Nevertheless, the average absorbed dose is also relatively high in different healthy organs. For example, [^177^Lu]Lu-rhPSMA-7.3 accumulates 2.3-fold more in the kidney and 2.2-fold more in the bone marrow than [^177^Lu]Lu-PSMA-I&T does, which raises concerns about side effects.^114^ Wurzer et al. developed a novel radiotracer, ^177^Lu-labelling rhPSMA-10.1, via the isomerization of ^177^Lu-labelling rhPSMA-7 and the substitution of DOTAGA with DOTA, to further improve the PK in normal organs while maintaining high tumour uptake comparable to that of [^177^Lu]Lu-rhPSMA-7.3.^115^ Dierks et al. reported that [^177^Lu]Lu-rhPSMA-10.1 was well tolerated and responded to PSA with durable radiological responses in all four patients evaluated.^116^ Formal clinical trials are currently in progress to assess its potential in a prospective setting (NCT05413850).

Copper-64 and copper-67 are promising groups of diagnostic and therapeutic radionuclides because of their chemical properties in terms of decay characteristics and half-life, which facilitate their use for sequential PET/CT imaging and radiotherapy via the same chelator. The macrobicyclic hexamine cage sarcophagine (sar) is an effective chelator that can form a kinetically inert and stable Cu^II^ complex. In 2019, Zia et al. reported two sar ligands tethered to single or double PSMA-targeting moieties. The monomeric formulation [^64^Cu]CuSarPSMA had a similar tumour uptake effect to that of [^68^Ga]Ga-PSMA-11, while the bivalent formulation [^64^Cu]CusarbisPSMA had significantly better tumour uptake and prolonged retention than the monomeric formulation.^117^ Furthermore, in 2021, McInnes et al. investigated the therapeutic potential of [^67^Cu]CuSarbisPSMA.^118^ The results showed that [^67^Cu]CuSarbisPSMA and [^177^Lu]Lu-PSMA-I&T exhibited similar tumour inhibitory effects and survival prolongation at equivalent doses; moreover, the shorter half-life of copper-67 than of lutetium-177 (61.9 h vs. 6.7 d) implies that dosing could be repeated over a shorter period, thus providing more control of fast-replicating tumours. [^64^Cu]CuSarbisPSMA and [^67^Cu]CuSarbisPSMA are being evaluated in a clinical trial for the detection and treatment of PSMA-positive mCRPC (NCT04868604).

Additionally, several patients with mCRPC failed to respond adequately to targeted β-radionuclide therapy ([^177^Lu]Lu-PSMA) or respond well initially but later develop resistance to this therapy. Therefore, α-particles are also potent for PSMA-targeting radiopharmaceuticals. Selcuk et al. reported their clinical study with [^225^Ac]Ac-PSMA treatment in patients with [^177^Lu]Lu-PSMA-refractory mCPRC, showing that [^225^Ac]Ac-PSMA therapy was effective and safe with manageable toxicity.^119^ This treatment has potential even in advanced mCRPC patients who have exhausted almost all current treatment options.

In terms of the future development of PSMA-targeting ligands, there is an emerging trend to design and develop PSMA-targeting ligands that enable diagnosis and therapy with the same ligand, which could avoid discontinuity in diagnostic integration and tumour uptake or PK differences owing to ligand replacement. On the other hand, attempting to incorporate more radionuclides into the development of PSMA-targeting radiopharmaceuticals is also an attractive strategy for the future. As a leading compound in targeted radionuclide diagnostics and therapeutics, PSMA-targeting radiopharmaceuticals will continue to be reinvented in the future.

#### Somatostatin receptor (SSTR)

SSTR is a cyclic neuropeptide containing 14 amino acid residues. There are more than five types of SSTRs, among which SSTR2 is the most common and abundant. The activity of SSTRs is mediated by interactions with G protein-coupled growth inhibitor receptors. The SSTR is also a pioneer target in the field of targeted radiopharmaceuticals and plays an essential role in the diagnosis, staging and treatment of NETs.^120^ NETs are a diverse group of neuronal and endocrine cell-derived malignancies; the majority of them are characterized by slow and indolent growth, leading to delayed diagnosis with approximately 50% of cases showing metastasis at the time of diagnosis.^121^

SSTR-targeting radiopharmaceuticals provide greater sensitivity and specificity than conventional modalities do and they are unignorable for NET patient radiotheranostics. SSTR-targeting RPTs have been widely implemented as front-line therapeutic options for metastatic/inoperable NETs. Currently, several types of SSTR agonists are attracting increasing interest in clinical and preclinical studies. [^68^Ga]Ga-DOTA-TATE, [^68^Ga]Ga-DOTA-TOC, and [^68^Ga]Ga-DOTA-NOC are commonly used radiopharmaceuticals in clinical practice.^122,123^ These three radiopharmaceuticals differ slightly in their structures and targeting capabilities, however, there is no clinically relevant difference for [^68^Ga]Ga-DOTA-TOC and [^68^Ga]Ga-DOTA-TATE in detecting NETs in patients.^123^ These radiopharmaceuticals are most frequently applied in PET/CT imaging for the diagnosis of NETs. The therapeutic agent [^177^Lu]Lu-DOTA-TATE led to significantly longer progression-free survival and a substantially improved response rate, thus opening a new clinical landscape for the first-line treatment of NETs.^124^ Notably, EB-conjugated [^177^Lu]Lu-DOTA-TATE also exhibited promising clinical efficacy. A study involving 32 patients with NETs who underwent multiple cycles of [^177^Lu]Lu-DOTA-EB-TATE therapy demonstrated that dose escalations of up to 3.97 GBq per cycle seem to be well tolerated and more effective than 1.17 GBq per cycle.^125^ Recently, they reported that an optimized long-acting somatostatin analogue-based radiopharmaceutical with linker substitution, [^177^Lu]Lu-LNC1010, was well-tolerated in patients with various types of NETs, resulting in an 83% disease control rate and a 42% overall response rate after two treatment cycles and 3.3 GBq per cycle was the most appropriate therapeutic dose for subsequent trials.^126^

More efforts have been reported to improve the properties of SSTR-targeting diagnostic and therapeutic radiopharmaceuticals. Owing to its long half-life and excellent energy properties, [^18^F]AlF-NOTA-octreotide is a new potential radiopharmaceutical with favourable properties because of its low background uptake, particularly in the liver, high PET/CT imaging quality and favourable lesion identification rates in NETs, similar to those of [^68^Ga]Ga-DOTA-TATE.^127^ Johnbeck et al. reported that [^64^Cu]Cu-DOTA-TATE had a significantly higher tumour detection rate than did [^68^Ga]Ga-DOTA-TOC in patients with NETs.^128^ A follow-up study revealed that more newly added true-positive lesions were detected by [^64^Cu]Cu-DOTA-TATE than by [^68^Ga]Ga-DOTA-TOC, which could be attributed to the shorter positron range of copper-64 than that of gallium-68. Notably, despite the success of [^177^Lu]Lu-DOTA-TATE in clinical applications, there is considerable scope to improve its safety and efficacy. ^212^Pb-targeted α-emission therapy is effective in further improving both of these aspects. Delpassand et al. reported that eight of ten patients who received all four cycles of [^212^Pb]Pb-DOTAM-TATE had a safe and promising clinical outcome (2.50 MBq/kg).^129^

Major progress in SSTR-targeting radiopharmaceuticals is the development of SSTR antagonists, which appear to engage more binding sites on the receptor with good PK properties and superior tumour imaging than SSTR agonists do. Cescato et al. reported 32 SSTR antagonist analogues and demonstrated that compound 3 and 31 had high SSTR2 binding affinity and selectivity.^130^ Based on the structure of compound 31, a phase I imaging study also demonstrated that [^68^Ga]Ga-NODAGA-JR11 had favourable PK and PET/CT imaging performance, with fast elimination from the blood, leading to low background accumulation, particularly in the liver and gastrointestinal tract.^131^ The effective dose of [^68^Ga]Ga-NODAGA-JR11 was similar to that of ^68^Ga-labelled SSTR agonists established clinically; and showed no obvious toxicity (NCT04897542). A recent PET/CT scan of four radiolabelled SSTR antagonists in 549 patients revealed that among [^68^Ga]Ga-NODAGA-LM3, [^68^Ga]Ga-DOTA-LM3, [^68^Ga]Ga-NODAGA-JR11 and [^68^Ga]Ga-DOTA-JR11, [^68^Ga]Ga-NODAGA-LM3 appeared to have the best imaging characteristics and deserves further clinical development because of its increased sensitivity and accuracy.^132^ Xie et al. reported that the quality analysis and excellent imaging performance of [^18^F]AlF-NOTA-JR11 for NETs were better than those of [^68^Ga]Ga-DOTA-TATE, especially in the patient’s digestive system with low background uptake, which allowed the detection of more SSTR-overexpressing lesions of the primary and metastatic regions with higher imaging contrast.^133^

The successful application of SSTR antagonist-based radiotracers indicated that ^177^Lu-labelling antagonists could be used instead of ^177^Lu-labelling agonists in RPT. In particular, a phase I study of [^177^Lu]Lu-DOTA-JR11 in well-differentiated NETs showed that [^177^Lu]Lu-DOTA-JR11 could deliver the required radiation levels to NETs with a superior tumour-to-normal organ dose ratio; however, in this trial, the primary treatment regimen produced more severe haematologic toxicity than the same dose of SSTR2 agonist did.^134^ Handula et al. conducted the first preliminary clinical evaluation of [^225^Ac]Ac-DOTA-JR11, showing that although both [^225^Ac]Ac-DOTA-JR11 and [^177^Lu]Lu-DOTA-JR11 exhibited comparable biodistribution patterns in vivo, [^225^Ac]Ac-DOTA-JR11 showed poor stability in PBS and mouse serum, and greater renal accumulation than did [^177^Lu]Lu-DOTA-JR11. Thus, further optimization of the PK of [^225^Ac]Ac-DOTA-JR11 is needed for safe and efficacious targeted α-particle therapy for NETs.^135^

Given their greater safety and affinity, multiple clinical studies have proven that the development and modification of SSTR-targeting antagonists are promising research directions for SSTR-targeting radiopharmaceuticals. Emerging radiopharmaceuticals, including somatostatin analogues labelled with fluorine-18 (to overcome the limitations imposed by ^68^Ga), actinium-225 and terbium-161 (to increase therapeutic efficacy), are also promising. In addition, the development of combination therapies and the exploration of new indications are critical directions for SSTR-targeting radiotherapy.

#### Integrin family

Integrins are members of the heterodimeric transmembrane glycoprotein family, which contains 18 distinct alpha subunits and eight beta subunits in mammals. Integrins transmit biomechanical signals across cells and their environment. The integrin receptor is a veteran useful target for radiopharmaceuticals. Integrin receptor expression differs widely between normal tissues and cancer tissues and is significantly correlated with cancer progression and metastasis.^136^ For example, integrins αvβ3 and αvβ6 are typically expressed at low or undetectable levels in most normal epithelial cells but are overexpressed in a wide range of tumours; this characteristic makes them suitable radiotheranostics targets of multiple cancers.^137^

Integrin αvβ3 is the most frequently studied integrin because it is a highly specific biomarker that plays an integral role in tumour metastasis and angiogenesis; therefore, PET/CT and SPECT/CT imaging targeting αvβ3 expression are very promising diagnostic strategies. The binding of αvβ3 to the vitronectin surface is mediated by the RGD (Arg-Gly-Asp) tripeptide, which acts as a core recognition motif. The earliest monomeric integrin-targeting PET tracer for use in clinical practice was [^18^F]galacto-RGD, an ^18^F-fluorinated RGD with added glycosylation, which was primarily used in gliomas imaging because of its lower uptake in normal brain tissue than [^18^F]FDG.^138^ Other derivates, including [^18^F]Fluciclatide and [^18^F]RGD-K5, subsequently appeared. However, the synthesis process of [^18^F]galactose-RGD and its derivates is complex and inefficient; therefore, they are not commercially available on a large scale.^139^ Compared with monomeric peptides, multimeric RGD peptide-based radiopharmaceuticals have relatively prolonged integrin-specific tumour retention times and better PK characteristics in vivo. [^18^F]FPPRGD2 is a targeted dimeric radiopharmaceutical developed via a polyethylene glycolated RGD dimeric peptide that binds to integrin-overexpressing tumours in vitro and in vivo. [^18^F]FPPRGD2 was found to be slightly superior in detecting several small metastatic foci that cannot be detected using [^18^F]FDG. Moreover, inflammatory lymph nodes and lesions that are false positive for [^18^F]FDG and negative for [^18^F]FPPRGD2 are later confirmed to be negative. Similarly, [^18^F]FPPRGD2 showed superior accuracy in detecting recurrent glioblastoma multiforme compared with brain MRI (100.0% vs. 93.3%), thus making it a promising diagnostic agent.^140^ Chen et al. developed a simplified radiolabelling procedure for the ^18^F-fluorinated RGD radiotracer, [^18^F]Alfatide II, which achieved automated production to improve commercial clinical feasibility. [^18^F]Alfatide II can be used for the diagnosis of metastatic axillary lymph nodes (ALNs) in BC patients; however, similar to [^18^F]FDG, it has limited sensitivity (70.59% vs. 64.71%). The sensitivity and negative predictive value are improved markedly by combining [^18^F]Alfatide II and [^18^F]FDG.^141^ A ^99m^Tc-labelled cyclic peptide containing a monomeric RGD tripeptide sequence, [^99m^Tc]Tc-3PRGD2, is valuable for detecting human cancers. Zhu et al. investigated the performance of [^99m^Tc]Tc-3PRGD2 SPECT imaging in the diagnosis of lung cancer. They reported that the majority of malignant lung tumours had good imaging quality at 1 h after administration of the tracer. The TBR was markedly greater than that of benign lesions. The majority of lymph nodes and bone metastases were also identified, suggesting that [^99m^Tc]Tc-3PRGD2 SPECT imaging is highly accurate for the diagnosis of lung cancer (sensitivity: 93–97%).^142^ Currently, [^99m^Tc]Tc-3PRGD2 has completed phase III clinical trials and has met both primary and secondary endpoints, which might be the first integrin-targeting radiopharmaceutical with potential for approval.

Integrin αvβ3 is a consistent and specific marker of ongoing angiogenesis; therefore, integrin αvβ3-targeting PET/CT imaging represents a novel noninvasive approach for the assessment of cardiovascular disease.^143^ The results from a retrospective analysis of data from 44 patients who underwent [^68^Ga]Ga-NODAGA-RGD PET/CT scans revealed that the arterial uptake of [^68^Ga]Ga-NODAGA-RGD was significantly greater in patients with previously clinically atherosclerotic cardiovascular disease, suggesting that [^68^Ga]Ga-NODAGA-RGD PET/CT imaging had the potential to be a non-invasive modality of atherosclerotic disease activity, providing information on angiogenesis within plaques.^144^

Integrin αvβ6 is emerging as a potentially useful biomarker for several cancers. The overexpression of integrin αvβ6 indicates poor prognosis and survival in various cancers and is associated with increased cancer metastasis. Therefore, integrin αvβ6-targeting imaging tracers would have significant clinical benefits.^145^ The ^18^F-fluorinated αvβ6-targeting peptide, BP(NAVPNLRGDLQVLAQKVART) can be used in clinical studies to detect multiple tumours. [^18^F]αvβ6-BP showed favourable specificity and selectivity for integrin αvβ6 in vitro (IC_50_ = 1.2 nM and >10 mM for αvβ6 and αvβ3), as well as high cell binding (72.5% ± 0.9%) and internalization (52.5% ± 1.8%) capacity. In patients, [^18^F]αvβ6-BP was generally tolerable with no severe adverse events. In a 63-year-old female patient with lung cancer, the uptake of [^18^F]αvβ6-BP was clearly visible in a primary lung cancer lesion (SUVmax: 5.2) and in a right iliac flank metastasis lesion (SUVmax 13.5).^146^ On the basis of BP, Ganguly et al. further developed and evaluated a novel αvβ6-targeting peptide, 5 G, radiolabelled with gallium-68 for imaging and with lutetium-177 for therapy. [^68^Ga]Ga-DOTA-5G and [^177^Lu]Lu-DOTA-ABM-5G are mainly cleared by the kidney, and the tumour uptake were 2.6 ± 0.8 and 5 ± 0.8%ID/g at 1 h and 72 h, respectively, in BxPC-3 model mice. Thus far, the average absorbed doses to the kidney and bone marrow are 3.7 Gy and 0.01 Gy, respectively. [^68^Ga]Ga-DOTA-5G detects tumours in patients with locally advanced or metastatic pancreatic ductal adenocarcinoma (PDAC), and [^68^Ga]Ga-DOTA-5G/[^177^Lu]Lu-DOTA-ABM-5G treatment is both safe and tolerable. Currently, patients are receiving treatment at a maximum dose of 7.4 GBq.^147^ Quigley et al. evaluated the PET/CT imaging ability of αvβ6-targeting [^68^Ga]Ga-Trivehexin in patients with head and neck cancer or pancreatic cancer and reported that the radiopharmaceutical binds to other integrins with submolar affinity (IC50 = 0.047 nM), and is highly selective for other isoforms (IC50-based factors: αvβ8, 131 nM; αvβ3, 57 nM; α5β1, 468 nM). In human clinical studies, PET/CT with a radiopharmaceutical has shown high levels and maintained accumulation in patients with metastatic PDAC and head and neck squamous cell carcinoma (HNSCC) (SUVmax = 10–13). [^68^Ga]Ga-Trivehexin enables the imaging of small PDAC metastases, and it has very low uptake in tissues with tumour-associated inflammation, thus demonstrating superior clinical utility.^148^ The ongoing development of integrin receptor-targeted radiopharmaceuticals continues to promote further advancements in RPT, especially for other integrins.^149^ However, the scarcity of optimal ligands should be urgently addressed, as exemplified by αvβ6, which has great promising potential for pancreatic cancer radiotherapy.

#### C-X-C chemokine receptor type 4 (CXCR4)

Chemokines are proinflammatory cytokines, with a molecular weight of 8–10 kDa, and they can activate specific white blood cells to generate a variety of immune/inflammatory responses through G protein-coupled receptor (GPCR) binding. Approximately 50 chemokines and their 20 corresponding receptors have been reported, among which CXCR4 is one of the most valuable targets. CXCR4 is overexpressed in more than 23 human cancers and contributes to tumour growth, invasion, and angiogenesis, thus making it an attractive and translationally promising molecular target.^150,151^

CXCR4-targeting peptide-based diagnostic and therapeutic radiopharmaceuticals are becoming increasingly popular in clinical settings. [^68^Ga]Ga-DOTA-CPCR4-2 (Pentixafor) exhibited high affinity for targeting CXCR4 (the IC_50_ value of [^nat^Ga]Ga-DOTA-CPCR4-2 was 4.99 ± 0.72 nM).^152^ Recently, Dreher et al. performed CXCR4-directed PET/CT using [^68^Ga]Ga-Pentixafor in 142 patients with histologically confirmed tumours, including 23 solid tumours. Scans from 67.8% of the patients revealed a median TBR of 4.4 (1.05–24.98) in 462 lesions with high imaging contrast. The authors also reported that the chemokine receptor levels remained virtually unchanged in patients, with no associated uptake differences between primary and metastatic foci, indicating that CXCR4-targeting imaging could serve as a powerful tool for screening patients with solid tumours.^153^ Specifically, 10 of 14 patients with advanced multiple myeloma (MM) presented manifestations of MM after [^68^Ga]Ga-Pentixafor PET/CT scans, whereas nine patients were visually positive on [^18^F]FDG PET/CT scans, suggesting that [^68^Ga]Ga-Pentixafor is more advantageous for the clinical diagnosis of MM.^154^

By linking amino acid modifications and the N-terminal ^99m^Tc-labelling strategy, Konrad et al. developed and comparatively assessed six mas_3_-conjugated CPCR4 (CXCR4-targeted ligand) analogs on the basis of pentixafor scaffold with N4-L6-CPCR4 (PentixaTec) having an enhanced hCXCR4 affinity of 0.6 ± 0.1 nM, [^99m^Tc]Tc-PentixaTec had the highest internalization efficiency (97% of all cellular activities within 2 h) and the maximum tumour uptake (8.6 ± 1.3%ID/g, 1 h). On SPECT imaging of five patients with haematologic malignancies, [^99m^Tc]Tc-PentixaTec showed good tolerability, biodistribution, and dosimetric profile (2.1–3.4 mSv per 500 MBq) and a favourable TBR.^155^ In a 65-year-old female patient with relapsed MM, SPECT/CT imaging with [^99m^Tc]Tc-PentixaTec revealed significant CXCR4 expression in the skin and muscle lesions, which may help guide the therapeutic approach. Given its lower cost and general availability, [^99m^Tc]Tc-PentixaTec could be an alternative to CXCR4-targeting PET tracers.^156^

Herrmann conducted the first-in-human study of CXCR4-targeting radiotherapy with [^177^Lu]Lu-Pentixather and [^90^Y]Y-Pentixather in three patients. A significant therapeutic effect was observed through a greater than 50% decrease in the difference between involved and noninvolved serum-free light chain levels in two patients. However, pentixather treatment caused bone marrow ablation in all three patients and might have promoted leukopenia and sepsis in one patient.^157^ A study of the biokinetics and dosimetry of [^177^Lu]Lu-Pentixather showed that [^177^Lu]Lu-Pentixather has a high uptake and prolonged retention time in the bone marrow, leading to high dose-specific uptake by the hematopoietic system. Therefore, the maximum safe dose of the tracer may not be sufficient to produce an adequate therapeutic effect on malignant tissue. Treatment with α-emitters could be a promising CXCR4-targeting therapy.^158^

[^68^Ga]Ga-Pentixafor and [^177^Lu]Lu-Pentixather are pioneer tracers for the detection and treatment of CXCR4-positive tumours. However, the challenge of excessive hepatic uptake due to multiple chemokine receptors in the normal liver and renal uptake should be addressed through the design and development of more specific and water-soluble antagonists of CXCR4.

#### Gastrin-releasing peptide receptor (GRPR)

The gastrin receptor is a member of the bombesin (BBN) family, which is a 14-residue peptide and its mammalian counterpart, gastrin-releasing peptide (GRP), is a 27-residue peptide with the same C-terminus as BBN, i.e., Trp-Ala-Val-Gly-His-Leu-Met-NH2. It is upregulated in a wide range of malignancies, such as BC, pancreatic cancer, lung cancer and CNS tumours.^159^ Many of these peptides are derivatives of the GRP/BBN C-terminal fragments with high specificity for the BBN receptor family and have been used as templates for receptor-mediated tumour imaging and radiolabelled peptide analogue therapy.^160^

The highly conserved sequence at the C-terminus of the peptide is essential for the bioactivity of BBN-like peptides and for binding to GRPRs. On the basis of this feature, Zhang et al. evaluated the imaging capability of [^68^Ga]Ga-NOTA-Aca-BBN (7–14) in children with optic pathway gliomas in the field of pediatric neuro-oncology. In eight patients, all 11 lesions displayed significant uptake with favourable imaging contrast. Compared with [^18^F]FDG, [^68^Ga]Ga-NOTA-Aca-BBN (7–14) had a remarkably greater TBR (28.4 ± 5.59 vs. 0.47 ± 0.11).^161^ Ananias et al. conducted a first-in-human pilot investigation using the BBN-based radiotracer [^99m^Tc]Tc-HABBN to investigate its feasibility of in eight patients with PCs. The radiopharmaceutical was safe, but demonstrated low in vivo metabolic stability in humans (less than 20% was intact after 30 min) as there was no uptake in the prostate at any time point in any patient.^162^

The interaction between GRP and its receptor promotes the growth of many human cancer cells. Therefore, efforts have been made to develop GRPR antagonists for the diagnosis and treatment of GRPR-positive tumours. The development of the potent GRPR antagonist [^68^Ga]Ga-RM1 resulted in superior preclinical performance compared with that of the agonist.^163^ Mansi et al. developed the antagonist RM2 with increased binding affinity through the positively charged spacer 4-amino-1-carboxymethyl-piperidine and both [^111^In]In-RM2 and [^68^Ga]Ga-RM2 displayed high specific uptake in LNCap (human PC cell line) tumours.^164^ Baratto et al. performed a study of [^68^Ga]Ga-RM2 uptake and distribution in 95 patients with biochemically recurrent PCs; the authors showed that [^68^Ga]Ga-RM2 could be used primarily for PC detection and for other tumours that overexpress GRPR, such as BC. However, strong radioactivity uptake was observed in the pancreas (average SUVmax: 64.91).^165^ Kurth et al. evaluated the uptake dose to the primary organs and tumour foci in a cohort of mCRPC patients with insufficient PSMA expression or low PSMA uptake following [^177^Lu]Lu-PSMA-617; the authors reported that [^177^Lu]Lu-RM2 can be used for mCRPC RPT because it showed high tumour accumulation and rapid elimination from normal organs, except for the pancreas, which has high uptake and is the dose-limiting organ.^166^ To increase the metabolic stability of RM2, another study replaced the metabolically destabilizing Gln7-Trp8 bond in L-Trp8 with α-methyl-L-tryptophan (α-Me-L-Trp) and obtained a novel ligand, AMTG, which was then subjected to a preclinical evaluation of [^177^Lu]Lu-AMTG. Compared with [^177^Lu]Lu-RM2, [^177^Lu]Lu-AMTG showed mildly improved GRPR affinity, greater in vitro and in vivo stability, and 24 h post-injection tumour retention (11.45 ± 0.43%ID/g).^167^ In the first human clinical trial, [^68^Ga]Ga-AMTG PET/CT revealed multiple significant lesions with strong focal uptake between the peritoneum, the subdiaphragmatic region adjacent to the liver, and the left internal and external iliac arteries. However, only a single lesion with low [^18^F]PSMA-1007 uptake was detected in the subdiaphragmatic lesion.^168^ Recently, the authors reported that [^161^Tb]Tb/[^177^Lu]Lu-AMTG may show greater therapeutic efficacy than [^161^Tb]Tb/[^177^Lu]Lu-RM2, particularly [^161^Tb]Tb-AMTG, because of its additional auger-electron emission at the cell membrane level.^169^

To obtain a more stable targeting ligand, Nock et al. replaced the C-terminal Leu13-Met14-NH2 dipeptide of SB3 with Sta13-Leu14-N, resulting in a new GRPR antagonist, NeoBOMB. [^68^Ga]Ga-NeoBOMB1 and [^177^Lu]Lu-NeoBOMB1 showed considerably greater GRPR-specific tumour uptake in PC-3 model mice (30.6 ± 3.9, 28.6 ± 6.0, and more than 35%ID/g, respectively, at 4 h post-injection). The activity of these tracers is characterized independently of the labelled metal and is cleared from major organs, especially the kidney.^170^ A phase I/IIa clinical trial of [^68^Ga]Ga-NeoBOMB1 in patients with oligometastatic gastrointestinal mesenchymal stromal tumours revealed an average effective dose of 0.029 ± 0.06 mSv/MBq. Significant uptake of the tracer was detected in the tumour lesions of three patients over time. The maximum SUV values was 4.3–25.9 at 2 h post-injection. The maximum organ dose was found in the pancreas (0.274 ± 0.099 mSv/MBq).^171^

On the basis of these findings, the clinical translation of GRPR-targeting radiopharmaceuticals is in demand, with antagonists being the main direction of development, and the challenge of high uptake in normal organs such as the pancreas should be optimized therapeutically.

#### Human epidermal growth factor receptor 2 (HER2)

HER2 is a member of the epidermal growth factor receptor (EGFR) family of receptor tyrosine kinases. Heterodimers with other members of the EGFR family activate multiple signalling pathways, leading to cell proliferation and tumourigenesis. HER2 expression is upregulated in several tumours, particularly in female patients with BC.^172^ Therefore, HER2-targeting diagnosis and treatment have been pursued with particular emphasis on developing more potent radiopharmaceuticals.^173^

Trastuzumab was the first FDA-approved mAb for HER2 + BC treatment. Dijkers et al. performed a preliminary study to determine the appropriate dose and timing of [^89^Zr]Zr-N-SucDf -trastuzumab ([^89^Zr]Zr-trastuzumab). The results suggested that the optimal time to measure the tumour uptake of [^89^Zr]Zr-trastuzumab was 4–5 days after injection. Notably, [^89^Zr]Zr-trastuzumab identified a bone lesion that was not detected by other scans. The maximum uptake was found in the liver lesions, which was significantly greater than that determined in the normal liver tissues (relative uptake values: 12.8 ± 5.8 vs. 5.9 ± 2.4); however, the difference in uptake between the various types of tumours was relatively large.^174^ Ulaner et al. demonstrated that HER2-targeting PET/CT imaging could be a candidate approach for identifying additional HER2-targeting therapies, showing that [^89^Zr]Zr-trastuzumab detected unrecognized positive metastases in patients with HER2-negative primary BC.^175^ Laforest et al. obtained similar results and reported that the liver was the dose-limiting organ.^176^ Tamura et al. reported that [^64^Cu]Cu-DOTA-trastuzumab targets brain metastases and the optimal time to assess the tumour uptake of [^64^Cu]Cu-DOTA-trastuzumab was 48 h after injection. The radiation dose for [^64^Cu]Cu-DOTA-trastuzumab imaging is comparable to that of [^18^F]FDG.^177^ The clinical application of trastuzumab is limited by side effects such as drug resistance and cardiotoxicity. Pertuzumab, which binds to HER2 at a different recognized site, appears more effective and has been approved by the FDA. In a first-in-human study, Ulaner et al. reported that a patient was recently diagnosed with brain metastases and [^89^Zr]Zr-DFO-pertuzumab PET/CT revealed progressively increasing [^89^Zr]Zr-DFO-pertuzumab uptake on post-treatment days 1, 2, 6, and 8 (SUVmax: 13.6, 16.6, 26.0 and 30.1, respectively).^178^

Compared with antibodies, HER2-targeting nanobodies, affibodies and microbodies with smaller sizes have superior PK properties while maintaining affinity and specificity for HER2. These features make them more suitable for tumour imaging. ABY-025, a small affibody molecule (6.5 kDa), targets a specific epitope of the HER2 receptor that is not covered by current therapeutics. Sörensen et al. reported that [^111^In]In-DOTA-ABY-025 had an average effective injectable dose of 0.15 mSv/MBq. In one patient, bone metastases showed enhanced [^18^F]FDG accumulation; but with low uptake of [^111^In]In-DOTA-ABY-025. This finding was confirmed by negative immunohistochemical staining of the biopsy samples, which indicated good specificity for [^111^In]In-DOTA-ABY-025.^179^ Nanobodies are advantageous for SPECT/CT and PET/CT imaging because of their high specificity and rapid elimination from the blood. Keyaerts et al. conducted a phase I trial of radiolabelled nanobodies, [^68^Ga]Ga-p-SCN-Bn-NOTA-HER2, for PET/CT imaging and reported that the effective dose was 0.043 mSv/MBq, with the bladder wall receiving the maximum organ dose of 0.406 mGy/MBq. Tracer uptake was observed in 86.7% (15 patients) of primary tumours (SUVmean: 0.7–11.8).^180^ The first clinical evaluation of ^18^F-fluorinated anti-HER2 nanobodies ([^18^F]AlF-RESCA-MIRC213, K_d_: 1.23 ± 0.58 nM) for HER2-positive cancer PET/CT imaging in patients was reported by Qin et al. Tumour uptake of [^18^F]AlF-RESCA-MIRC213 in HER2-positive patients (SUVmax: 3.62 ± 1.56) was significantly greater than that in HER2-negative patients (SUVmax: 1.41 ± 0.4) at 2 h post-injection.^181^ VHH1 is a single-domain antibody. A phase I trial of [^131^I]GMIB-anti-HER2-VHH1 in healthy volunteers and BC patients demonstrated that the radiotracer was well stabilized in the circulation system and that its accumulation was not increased in the thyroid gland or stomach, as patients were treated with potassium iodide for blocking pretreatment. The kidney showed the maximum uptake (1.54 ± 0.25 mGy/MBq). Highly injectable active [^131^I]GMIB-anti-HER2-VHH1 exerts a therapeutic effect, and the therapeutic value of this compound will be evaluated in a planned phase I/II dose escalation and extension study (NCT04467515).^182^

Peptides are better suited for clinical imaging procedures because of their relatively short half-life in circulation, good permeability, low immunogenicity, and ease of chemical modification. Li et al. prepared a targeted SPECT imaging radiotracer, [^99m^Tc]Tc-HYNIC-H6F, which is based on a HER2-targeting peptide. SPECT/CT imaging revealed apparent accumulation of the radiotracer in the MDA-MB-453 tumours (ROI = 3.58 ± 0.01%ID/g at 30 min post-injection), whereas HER2-negative MDA-MB-231 tumours presented considerably lower signals (ROI = 0.73 ± 0.22%ID/g at 30 min post-injection). As [^99m^Tc]Tc-HYNIC-H6F and trastuzumab bind to different sites of the HER2 receptor, this radiotracer also has the potential to monitor the therapeutic effect of trastuzumab by reexamining HER2 expression levels during treatment with no blockade effects.^183^

The development of smaller HER2-targeting ligands, such as nanobodies and peptides, has more clinical translational benefits. Additional strategies are needed for HER2-negative lesions to expand the scope of diagnosis and treatment of BC with HER2-based radiotracers.

#### Carbonic anhydrase isoform IX (CA IX)

Tumour-associated, membrane-bound human CA IX is upregulated in cancer cells, particularly in most clear cell renal cell carcinomas (ccRCCs) and contributes to hypoxic tumour pH/metabolic regulatory mechanisms. Thus, CA IX is a marker for malignant tissue and can be used to design radiotracers for PET/CT imaging.^184,185^

The mAb G250 (girentuximab), which was originally derived from hybridomas generated from mouse splenocytes immunized with human ccRCC, specifically recognizes an extracellular conformational epitope on the catalytic domain of CA IX. In 2013, Divgi et al. reported a large multicenter phase III clinical trial involving 226 patients treated with the ^124^I-iodinated antibody, [^124^I]girentuximab. The authors reported that [^124^I]girentuximab was well tolerated and showed favourable results in ccRCC CA IX-targeting PET imaging, with a mean sensitivity and specificity of 86.2% and 85.9%, respectively, for PET/CT imaging with [^124^I]girentuximab compared with contrast-enhanced computed tomography (CECT).^186^ However, ^124^I-based immuno-PET agents have general disadvantages in clinical practice, such as the high cost of radionuclides and the tendency of ^124^I-iodinated PET tracers to undergo dehalogenation in vivo, leading to radionuclide accumulation in the thyroid glands. This prompted the entry of [^89^Zr]Zr-DFO-girentuximab into the phase I/II trials. Heckman et al. reported that all PET-positive patients in subgroup 1 were confirmed to have aggressive ccRCC, and PET/CT imaging of [^89^Zr]Zr-DFO-girentuximab detected additional metastases.^187^ Merkx further demonstrated that the liver received the highest dose (1.86 ± 0.40 mGy/MBq), and the median absorbed dose in the tumour was 4.03 mGy/MBq. This finding suggested that [^89^Zr]Zr-DFO-girentuximab is safe and tolerable and can be quantitatively assessed by [^89^Zr]Zr-DFO-girentuximab PET/CT.^188^ Verhoeff and colleagues estimated the efficacy of baseline contrast-enhanced CT, [^89^Zr]Zr-DFO-girentuximab-PET/CT, and [^18^F]FDG-PET/CT for lesion detection in patients with ccRCC who had good or intermediate prognoses. The results showed that the combination of [^89^Zr]Zr-DFO-girentuximab-PET/CT and CT identified more lesions (91%) than CT alone (56%). Moreover, the overall geometric mean SUVmax of [^89^Zr]Zr-DFO-girentuximab was 15.5 for lesions and 4.4 for [^18^F]FDG. This method was also more effective in identifying metastases than CT and [^18^F]FDG-PET/CT.^189^

Small-molecule ligands have received increasing attention in the development of CA IX-targeting radiotracers because of their low production cost, non-immunoreactivity, and tunable PK. [^18^F]VM4-037 is the first small-molecule CA IX-targeting PET radiotracer that has entered into clinical trials. Whole-body PET/CT imaging of normal volunteers revealed a high accumulation of [^18^F]VM4-037 in the kidney and liver, with uptake doses of 273 ± 31 and 240 ± 68 μGy/MBq, respectively, making it unsuitable for imaging CA IX upregulation in these two organs.^190^ A phase II pilot study of [^18^F]VM4-037 further demonstrated that it is difficult to visualize primary ccRCC lesions because of the significant uptake in healthy kidney tissue. However, extrarenal ccRCC lesions can still be effectively identified.^191^ Acetazolamide is a high-affinity small-molecule ligand for CA IX. Kulterer et al. investigated the targeting properties and safety of [^99m^Tc]Tc-PHC-102 in five patients with localized or metastatic ccRCC. Although organs such as the kidney exhibited strong radioactivity accumulation, significant uptake of the tracer was observed in the primary tumours of all five patients with high TBR values. Importantly, [^99m^Tc]Tc-PHC-102 SPECT/CT identified four undetected lung and lymph node metastases in two patients.^192^

Recently, Zhu et al. investigated the application of [^68^Ga]Ga-NOTA-NY104, a small-molecule CAIX-targeting PET agent with an acetazolamide core and NOTA as a chelator, in a ccRCC tumour model and in patients with diagnosed or suspected ccRCC. [^68^Ga]Ga-NOTA-NY104 accumulated in OS-RC-2 tumours within 5 min, and its amount progressively increased for 3 h after injection, with an SUVmax of 29.29 ± 6.82%ID/g. Moreover, patients 1 and 2 tolerated [^68^Ga]Ga-NOTA-NY104 well, with an SUVmax of 42.3. In the third patient, the lesion was accurately diagnosed as a non-metastatic lesion on the basis of the negative uptake of [^68^Ga]Ga-NOTA-NY104.^193^

Owing to the excellent diagnostic performance of CA IX in RCC, the development of non-renally metabolized CAIX-targeting small molecule ligands is more advantageous in terms of increased specificity and detection of clinical renal lesions. CA IX ligands, represented by cyclic peptides and mimetic peptides, have been designed and entered clinical trials for RPT. The future development of cyclic peptide ligands with subnanomolar affinity for both diagnostic and therapeutic applications has good prospects for clinical translation.

#### Urokinase-type plasminogen activator receptor (uPAR)

The uPAR glycoprotein comprises 313 amino acids, which enables the binding of uPA on the cell surface and the regulation of protein hydrolysis to the invasive margins of cancer cells. uPAR can also modulate several activities, including tumour progression and metastasis.^194^ The elevated expression of uPAR in cancer cells makes it not only a valuable diagnostic tool, but also an attractive target for therapeutic intervention.^195^

In 2001, Ploug et al. used combinatorial chemistry to develop and validate a 9-polymer linear peptide antagonist, AE105 (IC_50_: 0.36 nM), which is a promising synthetic peptide antagonist that binds specifically to human uPAR.^196^ In 2012, Persson et al. performed PET/CT imaging of [^64^Cu]Cu-DOTA-AE105 in three mouse models with different uPAR expression levels. The authors reported a clear association between tumour accumulation and uPAR expression (R^2^ = 0.73; P < 0.0001).^197^ The authors also evaluated the safety, biodistribution and dosimetry of [^64^Cu]Cu-DOTA-AE105 in 10 cancer patients. An SUVmax of 12.5 was achieved in Patient 9 with a PC. The effective dose of tumour was 0.0276 mSv/MBq, with the highest absorbed dose in the liver (0.175 mGy/MBq).^198^

Considering that the use of copper-64 is hampered in clinical practice because of its limited supply and the need for a cyclotron facility for production, the authors conducted a phase I clinical study of [^68^Ga]Ga-NOTA-AE105 to validate its feasibility for tumour imaging in 10 patients. The radiotracer exhibited favourable in vivo stability, which was rapidly cleared from normal organs and was primarily excreted by the kidney. The effective dose was 0.015 mSv/MBq. Additionally, radiotracer uptake has been noted in both local tumour lesions and metastatic lesions.^199^ In the [^68^Ga]Ga-NOTA-AE105 PET/CT phase II clinical trial of 96 patients with neuroendocrine neoplasms (NENs), the hazard ratio for progression-free survival and OS with a high uPAR expression level was 1.19–5.88. The authors concluded that uPAR-PET is a valuable tool for risk stratification.^200^ The results from an additional phase II clinical trial revealed that compared with [^18^F]FDG, [^68^Ga]Ga-NOTA-AE105 was potentially valuable in identifying patients suitable for de-escalation therapy and risk-stratified follow-up protocols.^201^

Given the advantages in terms of the half-life and wide availability of fluorine-18, Persson and colleagues also developed the earliest ^18^F-fluorinated uPAR-targeting ligand by using Al^18^F radiolabelling. PET/CT images revealed that the tumour uptake was 5.90 ± 0.35%ID/g on the basis of a region of interest (ROI) analysis at 0.5 h post-injection and the uptake increased with decreasing time. [^18^F]AlF-NOTA-AE105 is primarily excreted through the kidney, with relatively high radioactivity uptake in bone.^202^

For uPAR-RPT, [^177^Lu]Lu-DOTA-AE105 substantially decreased the number of metastatic lesions in disseminated metastatic PC tumour-bearing mice compared with that in control mice; moreover, 65% of the mice did not develop disseminated metastatic lesions at 65 days after the initial administration.^203^

AE105 has become the hallmark of uPAR-targeting radiotracers and coupling it with more radionuclides with excellent properties is favourable for expanding the clinical application of uPAR-targeting radiopharmaceuticals. PET/CT imaging of uPAR in cancer is still in its infancy, and notably, the sensitivity of PET/CT imaging with uPAR ligands varies from tumour to tumour. With the widespread use of high-throughput screening techniques, it is critical to develop a greater number of uPAR ligands with high levels of specificity.

#### Metabotropic glutamate receptor 1 (mGluR1)

mGluR1, a seven-transmembrane domain GPCR, is known for its critical regulatory role in brain disorders and the nervous system. Recently, mGluR1 has been intensively studied as a molecular target for tumour imaging because it is upregulated in several cancers, including melanoma, BC, and lung cancer.^204^

In 2012, Fujinaga et al. developed three novel PET tracers, [^11^C]6, [^18^F]7 and [^18^F]8, demonstrating the effectiveness of [^11^C]6 for monitoring mGluR1 expression in the rodent brain.^205^ On the basis of the studies with multiple radioligands in brain imaging, the authors further reported the first imaging of mGluR1 in melanoma using [^18^F]7. By replacing methoxy groups with fluorine-18, they developed an oncoprotein-based PET/CT imaging platform for non-invasive imaging and quantification of melanomas mGlu1 via a novel targeting radiotracer, [^18^F]FITM. The [^18^F]FITM was accumulated in tumours within the first 30 min following injection and reached peak values of 5.75–7.46%ID/g after 120 min.

However, the high accumulation and slow clearance from the brain reduce the selectivity of this tracer for identifying tumours from healthy brain tissues to non-targeted organs and could cause radiation-induced damage.^206^ In 2015, the authors designed three mGluR1-targeting PET tracers: [^11^C]4-6, which showed comparable tumour uptake rates (∼4.0–4.2%ID/g at 60 min). However, the brain uptake of these tracers decreased with increasing atomic number of the halogen atom, which might be partly attributed to the lipophilicity of [^11^C]6 together with a reduced binding affinity for mGluR1 compared with that of other radiotracers. Strong lipophilicity might cause [^11^C]6 to bind to blood albumin, limiting its entry into the brain.^207^ These interesting findings prompted the authors to label IITM with the commonly available halogen isotope iodine-131 and the α-emitting radionuclide astatine-211, which exhibit potent halogen properties. The authors then developed two new mGluR1-targeting radiopharmaceuticals, [^131^I]IITM and [^211^At]AITM, for melanoma RPT studies. The maximum tumour uptake rates were 4.66 ± 0.70 and 7.68 ± 0.71%ID/g, respectively, and both were rapidly eliminated from the non-targeted organs after injection. The tumour volume was reduced by approximately 61% in the [^131^I]IITM group compared with that in the control group; moreover, [^211^At]AITM showed better therapeutic efficiency (reduction in tumour volume by 73.48% at a 1.11 MBq dose).^208^ Because most conventional strategies regulate mGluR1 activity through antagonists, this approach is susceptible to metabolic compensatory regulation. Additionally, few studies have investigated how tumour protein phenotypes change after irradiation. The authors also reported that [^211^At]AITM-based RPT is a one-two-punch strategy to combat mGluR1+ pancreatic cancer. [^211^At]AITM-based therapy not only reduces mGluR1 expression but also induces cancer senescence through reprogrammed senescence-associated secretory phenotype (SASP) variants, which together modulate cell fate through phenotypic changes in synergy with genetic alterations.^11^

Owing to insufficient clinical and preclinical studies, the development of more novel ligands, new clinical indications, and exploration of the mechanism of action may be the next direction for the development of mGluR1-targeting radiopharmaceuticals. On the other hand, exploring the regulatory role and underlying mechanisms of mGluR1 in tumour metabolism via mGluR1-targeting tracers is highly important.

#### Neurotensin receptor 1 (NTSR-1)

Neurotensin (NTS) is a peptide with 13 amino acid residues, and it functions as a messenger or neuromodulator in the CNS. NTS predominantly interacts with NTSR-1 to regulate a range of oncogenic processes including cell proliferation, migration, invasion and neoangiogenesis. NTSR-1 is overexpressed early during tumourigenesis in many cancers, suggesting that the neurotrophin signalling pathway plays a critical role in the development of cancer diagnosis and therapy.^209^

Leonte et al. reported that [^68^Ga]Ga-DOTA-NT demonstrated substantial tumour accumulation in HT-29 colon tumour-bearing mice (11.56 ± 2.14%ID/g at the tumour margin and 4.5 ± 0.8%ID/g at the tumour centre). However, the uptake of [^177^Lu]Lu-DOTA-NT in the renal system and bone marrow was high, which might hinder its clinical application.^210^ Wu et al. developed a series of ^18^F-fluorinated NT-targeting radiotracers, including [^18^F]DEG-VS-NT, which were synthesized easily and achieved substantially enhanced in vivo contrast; moreover, the high tumour-to-muscle ratios (30.65 ± 22.31) of this tracer were maintained at 2 h after injection. The authors also reported that the tumour-to-muscle ratio of [^18^F]AlF-NOTA-NT was 7.3; thus, this tracer can be used as a diagnostic agent for NTSR-targeting therapies, thereby enabling personalized theranostics.^211^

Using a selective and non-peptide antagonist of NTSR, SR48692, Schulz et al. reported three ^111^In-substituted antagonists of NTSR, 3BP-227, 3BP-228 and 3BP-483, and SPECT/CT imaging revealed high-contrast tumour visualization for the three compounds. In vitro biodistribution studies revealed that [^111^In]In-DOTA-3BP-227 exhibited strong and sustained tumour accumulation, peaking at 6 h post-injection (8.4 ± 3.1%ID/g).^212^ The effects of its ^177^Lu-labelled analogue on NTSR-1-positive HT-29 colon tumour-bearing mice were subsequently investigated. Biodistribution studies revealed high and sustained accumulation of [^177^Lu]Lu-DOTA-3BP-227 in HT29 model mice (19.0 ± 3.6%ID/g and 2.7 ± 1.6%ID/g at 3 and 69 h post-injection, respectively). The average relative tumour volume in the mice injected with 165 MBq of [^177^Lu]Lu-DOTA-3BP-227 was decreased by 88% compared with that in the control groups.^213^ Baum et al. reported the first clinical study of [^177^Lu]Lu-DOTA-3BP-227 in patients with metastatic PDAC. High kidney uptake was observed, and the most severe side effect was reversible grade 2 anaemia. One patient survived for 11 months after the start of therapy with [^177^Lu]Lu-DOTA-3BP-227.^214^

Owing to their advantages of better binding affinity and lower normal organ uptake than agonists do, NTSR-1 antagonists are still the leading ligands in clinical research and translation. In terms of indications, exploring the benefits of NTSR-1-targeting radiopharmaceuticals in the imaging and treatment of primary and metastatic pancreatic cancer will receive more widespread attention in the future.

#### Claudin-18 isoform 2 (Claudin18.2)

Claudins are a group of proteins that function as essential modulators of cellular tight junctions, which form a paracellular barrier that regulates the flow of molecules between cells. Claudins are aberrantly expressed in different tissues, and their altered expression is associated with cancer progression. For example, claudin-18 expression has prognostic significance in gastric cancer. The expression of claudin-18.2 in normal tissues is restricted to the differentiated epithelial cells of the gastric mucosa. Claudin-18.2, however, is highly expressed in primary gastric cancers and their metastatic lesions, making it an attractive target for diagnostic and therapeutic applications.^215^

Hu et al. reported three anti-CLDN18.2 antibodies that were radiolabelled with zirconium-89 for PET/CT imaging. Among these antibodies, anti-CLDN18.2 VHH-ABD was the most favourable, with tumour accumulation of 21.46 ± 1.78%ID/g and liver uptake of 6.70 ± 0.74%ID/g.^216^ Chen et al. developed an ^89^Zr-labelled-recombinant humanized CLDN18.2 antibody, TST001, to monitor the expression of CLDN18.2 in human gastric cancer tissues. The authors demonstrated strong tumour accumulation of the radiotracer in the BGC823^CLDN18.2^ mouse models. Biodistribution assays revealed greater tumour uptake in BGC823^CLDN18.2^ model mice than in BGC823 model mice (2.05 ± 0.16%ID/g and 0.69 ± 0.02%ID/g, respectively). The effective dose of [^89^Zr]Zr-DFO-TST001 was 0.0705 mSv/MBq.^217^ The authors also evaluated the efficacy of [^177^Lu]Lu-DOTA-TST001 against CLDN18.2-positive tumours. The tumour volume in the high-dose group (11.10 MBq) was markedly lower than that in the control group, and no apparent hematotoxicity or liver toxicity was observed.^218^ Wang et al. conducted a single-centre, phase 0 study to assess the safety, distribution, dosing, and specificity of a ^124^I-iodinated CLDN18.2-targeting scFv-Fc fusion protein, [^124^I]18B10(10 L), in 17 patients. High accumulation of [^124^I]18B10(10 L) was observed in the spleen and liver. The SUVmax of the radiotracer accumulation in the tumour lesions was 0.4–19.5. Notably, PET/MR imaging demonstrated high uptake of the tracer in the lymph nodes adjacent to the left iliac vessel (SUVmax = 6.6).^219,220^

The high cost of manufacturing humanized mAbs and the prolonged imaging cycle hamper the clinical translation of antibody-based radiotracers. Therefore, developing CLDN18.2-targeting antibody derivatives and protein scaffold-mimicking antibodies is essential. Wei et al. developed three CLDN18.2-targeting nanobody-based radiotracers, [^68^Ga]/[^18^F]/[^64^Cu]Ga-NOTA-hu19V3T.^221^ [^68^Ga]Ga-NOTA-hu19V3T imaging enables visualization of minial subcutaneous Chinese hamster ovary (CHO)-CLDN18.2 lesions; however, further refinement of this tracer is needed to address the issues of stomach clearance and high kidney uptake. To confirm whether the kidney uptake of the radiotracer might diminish with a longer imaging time, the authors performed immunoPET/CT scans with [^64^Cu]Cu-NOTA-hu19V3 and reported that the kidney uptake decreased over time (13.73 ± 2.16%ID/g at 1 h and 0.11 ± 0.05%ID/g at 48 h); however, the ability of the tracer to detect disseminated CHO lesions expressing CLDN18.2 was almost absent. To address this, the authors then developed a CLDN18.2-targeting tracer, [^18^F]hu19V3. This tracer was eliminated mainly from the urinary and hepatobiliary systems, and it enabled the imaging of disseminated CHO lesions with CLDN18.2 overexpression (2.10% ± 0.53%). Recently, Wang et al. developed a CLDN18.2-specific peptide, T37. The highest SUVmax value of [^68^Ga]Ga-DOTA-T37 was 0.48 ± 0.01 at 15 min, and the renal uptake rapidly decreased over time, which needs to be further optimized.^222^

As a newcomer in radiopharmaceuticals, several clinical results have shown the superiority of CLDN18.2. However, in order to achieve simultaneous targeting for RPT, further development of ligand, such as peptides with high specificity, and additional clinical studies are needed to fully assess the clinical value of this target.

#### Cluster of differentiation 38 (CD38)

CD38 is a transmembrane type II glycoprotein that is overexpressed in plasma cells and MM cells. In recent decades, CD38 has emerged as a valuable molecular target for treating MM. Daratumumab is a human anti-CD38 IgG1 antibody developed by Genmab and was approved by the FDA in 2020 for treating adult patients with newly diagnosed or relapsed/refractory MM. Concurrently, advancements in CD38-targeting PET/CT imaging are progressing into translational clinical trials, which present promising outcomes for myeloma detection.^223^

Researchers have developed a series of immunoPET tracers based on daratumumab for tumour imaging and therapy. Caserta et al. reported that [^64^Cu]Cu-DOTA-daratumumab showed superior performance as an immunoPET tracer for MM imaging compared with [^18^F]FDG. The use of [^64^Cu]Cu-DOTA-daratumumab in the diagnosis of patients with MM has entered phase I clinical trials. Moreover, both preclinical and early clinical trials have utilized [^89^Zr]Zr-DFO-daratumumab, and the results have shown the ability of [^89^Zr]Zr-DFO-daratumumab to detect MM in human patients whose diagnosis was missed by [^18^F]FDG.^224^

Because radiolabelled daratumumab has limited tumour imaging ability at early time points, Wang et al. used gallium-68 to label Nb1053, a nanobody with a smaller molecular weight, which addressed the issue of mismatch of the half-lives between the mAb and copper-64/zirconium-89.^225^ [^68^Ga]Ga-NOTA-Nb1053 showed an average tumour uptake of 1.76 ± 0.305%ID/g, and has high specificity for CD38, with a tumour-to-muscle ratio of 10.90 and a tumour-to-bone ratio of 5.79. [^64^Cu]Cu-NOTA-Dara-F(ab′)2, which showed rapid and high tumour uptake at 2 h after injection (6.9 ± 1.2%ID/g) and peaked (9.5 ± 0.7%ID/g) at 12 h.^226^ Apart from the marketed drug daratumumab, the anti-CD38 mAb OKT10 is also used as a key component of the CD38-targeting antibody.^227^ The [^211^At]CD38 mAb has been proven to be an effective α-emitting radiolabelled mAb, leading to durable relief and prolonged patient survival (>150 days).^228^

In addition, addressing the clinical limitations of [^18^F]FDG, such as the diagnostic accuracy of MM, is an important research direction for the development of CD38-targeting radiopharmaceuticals. Moreover, the development of specific, smaller CD38-targeting ligands may have greater safety and translational value in clinical radiotherapies.

#### Glypican-3 (GPC3)

GPC3 is a heparan sulphate proteoglycan that is overexpressed on the surface of HCC cells. It belongs to the glypican family and binds to the cell membrane through a glycolphosphatidylinositol anchor. Previous studies have shown that GPC3 is a highly specific biomarker for HCC diagnosis. Although several GPC3-targeting ligands are presently under clinical investigation for treating HCC, the clinical translation of GPC3-targeting imaging is still limited, underscoring the urgency to find targeting ligands with high-affinity specificity and excellent PK properties.^229^

To date, ^124^Iodine-labelled GPC3-targeting antibody, [^124^I]codrituzumab has entered the clinical trial stage. A phase Ib study (NCT00976170) revealed that among the 14 patients who underwent PET scans, 13 had tumour-positive signals, and 6 of the 13 patients had significant uptake of [^124^I]codrituzumab with SUVs greater than 9.^230^ Sham et al. used the smaller targeting moiety F(ab’)2 of αGPC3 to create a better immunoPET/CT imaging tracer [^89^Zr]Zr-DOTA-αGPC3-F(ab’)2, which had a lower blood half-life of 11 h and allowed high-quality imaging on PET at 4 h post-injection, with a peak contrast ratio of 23.3 between tumour and the liver.^231^ PET/CT imaging of ERY974, a bispecific antibody, revealed that the tumour uptake of the antibody was associated with T cell infiltration.^232^ The authors found that [^89^Zr]Zr-N-suc-Df-ERY974 was preferentially distributed in T cell-rich tumour tissues. Moreover, An et al. and Fayn et al. developed GPC3-targeting immunoPET/CT imaging radiotracers by conjugating a single domain antibody (sdAb) with radionuclides. An et al. labelled sdAb G2 with gallium-68 and fluorine-18, and the tumour-to-muscle ratios were 6.58 ± 0.28 and 12.93 ± 3.01, respectively.^233^ Fayn et al. coupled native HN3 (nHN3)-DFO with zirconium-89 to yield [^89^Zr]Zr-DFO-ssHN3 and [^89^Zr]Zr-DFO-nHN3, and both of these tracers showed high tumour uptake (7.2 ± 1.2 and 5.7 ± 1.8%IA/g, 1 h after injection, respectively) and decreased accumulation in the liver.^234^ To develop GPC3-targeting RPT agents, Labadie et al. conjugated the chelator Octapa to a GPC3-targeting antibody (αGPC3) for radiolabelling of thorium-227, which showed optimal tumour killing effects after 23 days of treatment.^235^

In addition to antibodies, peptides can also be used as ligands for GPC3-targeting radiopharmaceuticals. Wang et al. labelled GPC3-targeting peptide L5 with 5-carboxyfluorescein (FAM) and Al^18^F, yielding [^18^F]AlF-NOTA-MP-6-Aoc-L5, which clearly represented imaging visualization but a low tumour-to-liver ratio (0.93 ± 0.16).^236^ Therefore, the chemical structure of the GPC3-targeting radiotracer remains to be modified to reduce its liver accumulation. Berman et al. reported that L5 and another GPC3-targeting peptide, TJ12P1 (DHLASLWWGTEL), had insufficient selectivity for use in accurate tumour imaging.^237^ To address this, Qin et al. used a two-step phage display to screen the GPC3-specific binding peptide TJ12P2 (SNDRPPNILQKR). Following ^18^F-fluorination, [^18^F]AlF-NOTA-TJ12P2 accumulates in liver tumours (1.825 ± 0.296%ID/g in the HepG2 tumours and 1.575 ± 0.520%ID/g in the SMMC-7721 tumours).^238^ Li et al. developed a promising PET imaging radiotracer, namly [^18^F]AlF-GP2633, revealed increased uptake in HepG2 tumor (3.37 ± 0.35% ID/g) and reduced accumulation in liver (1.70 ± 0.26%ID/g) at 1 hour post-injection.^239^ In addition, Xu et al. synthesized the SPECT radiotracer [^99m^Tc]Tc-(tricine)-(TPPTS)HYNIC-PEG4-THVSPNQGGLPS ([^99m^Tc]Tc-HPG). The authors reported that the maximum tumour-to-muscle ratio was 11.55 ± 0.54 at 1 h post-injection.^240^

Owing to its high specificity and accuracy in the diagnosis and treatment of HCC, GPC3 has great potential for clinical translational applications, highlighting the importance of developing highly specific GPC3-targeting ligands. Along with the development of new high-screening technologies, the development of peptide ligands with submolar affinity is a critical requirement.

#### Nectin cell adhesion molecule 4 (Nectin-4)

Nectin-4 is a type I transmembrane protein that belongs to the related immunoglobulin-like adhesion molecule family, and is thought to modulate Ca^2+^-independent cell‒cell adhesion at adheren junctions by recruiting cadherins. Several studies have confirmed that Nectin-4 is upregulated particularly in bladder cancer and BC cell membranes, making it a potential target for bladder cancer and BC.^241^

A novel Nectin-4-targeting antibody‒drug conjugate (ADC) (ASG-22ME) was developed by Challita-Eid et al. This ADC comprises a fully human-targeting Nectin-4 antibody and the potent microtubule-disrupting agent monomethyl auristatin E segment.^242^ Campbell et al. performed a preclinical evaluation to determine the biodistribution and tumour binding capacity of [^89^Zr]Zr-DFO-AGS-22M6 and [^18^F]AGS-22M6.^243^ The results revealed an average high uptake of [^89^Zr]Zr-DFO-AGS-22M6 (45.3 ± 2.4%ID/g) in MDA-MB-231-Nectin-4 tumours in mice. To further study the biodistribution properties, researchers used [^18^F]AGS-22M6 instead of [^89^Zr]Zr-AGS-22M6 and conducted 4-h dynamic PET scans on cynomolgus monkeys. The heart and bladder of two cynomolgus monkeys had the highest uptake of the radiotracer; however, further investigations are needed to determine whether the bladder uptake is due to the specific uptake of [^18^F]AGS-22M6 or elimination of the radiotracer. Ren et al. used the enfortumab vedotin as the targeting ligand.^244^ Iodine-124/125 labelling revealed that Nectin-4-positive SW780 cells had increased uptake of 4.05 ± 0.32%IA/5×10^5^ cells at 8 h, and the SUVmax was 0.60 ± 0.01 at 2 h p.i. and 1.50 ± 0.01 at 24 h p.i. These findings indicated that [^124/125^I]enfortumab vedotin could serve as a radiotracer in clinical application. Duan et al. developed a Nectin-4-targeting radiotracer [^68^Ga]Ga-DOTA-BT009 ([^68^Ga]Ga-N188) based on the bicycle toxin conjugates (BTCs) BT8009 designed by Mudd et al. in 2022.^245^ A preclinical experiment revealed that its uptake in SW780 tumours at 1 h post-injection was 2.94 ± 0.36%ID/g, which was notably greater than the uptake in the blocking group (1.44 ± 0.17%ID/g) and the Nectin-4 negative tumours (2.02 ± 0.22%ID/g). A subsequent clinical trial (NCT05321316) was conducted to investigate the ability of [^68^Ga]Ga-N188 to detect Nectin-4 upregulation in patients with urothelial carcinoma, particularly in patients with recurrent or advanced bladder cancer; the results confirmed that [^68^Ga]Ga-N188 can be used as an efficient PET tracer for imaging Nectin-4.^246^

Ongoing clinical trials of radiopharmaceuticals that target Nectin-4 have shown promising results. The further development of more novel Nectin-4-targeting ligands, such as bicyclic peptide ligands, will accelerate the clinical translation of Nectin-4-targeting radiopharmaceuticals because of their excellent targeting specificity and well-defined indications.

#### Vascular endothelial growth factor receptor (VEGFR)

VEGF plays a crucial role in vasculogenesis and angiogenesis. Although VEGF primarily binds to endothelial cells, previous studies have demonstrated its influence on non-endothelial cells, including tumour cells. VEGF can mediate tumour pathogenesis mainly through its neovascularization effect. VEGF is not only critical for maintaining vascular homoeostasis in normal tissues, but also has an essential role in the molecular pathogenesis of tumour growth and metastasis.^247^ VEGF receptor 1 (VEGFR1), a high-affinity tyrosine kinase, was reported in 1992,^248^ and subsequent studies confirmed that VEGFR2, which has low affinity and high homology, was the main signal transduction receptor of VEGF.^249^ Emerging evidence indicates that VEGFRs, which were initially thought to be exclusively expressed in endothelial cells, are also expressed in tumour cells; this finding suggests that anti-VEGF therapy could function on both tumour cells and endothelial cells.^250^ To achieve optimal treatment outcomes, the use of targeted VEGFR therapy requires precise dose optimization and continuous monitoring of treatment response. The development of a noninvasive and reliable quantitative method to assess VEGFR expression in vivo by using PET/CT imaging can facilitate the personalization of VEGFR-targeting chemotherapy.

There are several radiotracers that use the natural ligands of VEGFR as ligands. VEGF121 interacts with VEGFR1 and VEGFR2, and promotes the proliferation and migration of endothelial cells.^251^ Kang et al. conjugated VEGF121 with p-SCN-NOTA, and reported that the accumulation of [^68^Ga]Ga-NOTA-VEGF121 reached 2.73 ± 0.32%ID/g in U87MG tumour-bearing mice at 2 h post-injection.^252^ In 2018, Rainer et al. firstly evaluated the prognostic value of [^123^I]VEGF in patients with brain tumours; the authors reported that [^123^I]VEGF scintigraphy yielded positive results only in WHO grade IV glioma, whereas less malignant tumours, such as Grade II and III gliomas and B-cell lymphoma, presented negative [^123^I]VEGF scintigraphy findings.^253^

In recent years, many immunoPET radiotracers based on VEGFR antibodies have been developed. Bevacizumab (Avastin®) is a mAb that targets VEGF-A by neutralizing its signal through VEGFR-1 and VEGFR-2 receptors. Bevacizumab can suppress various VEGF-driven cellular responses, including cell proliferation, survival, migration, and tissue factor production.^254^ Gaykema et al. performed a clinical feasibility study on the application of [^89^Zr]Zr-*N*-sucDf-bevacizumab PET/CT for diagnosing BC patients for the first time.^255^ The results revealed that tumour uptake of [^89^Zr]Zr-*N*-sucDf-bevacizumab was observed in 96.1% of the primary lesions, and the SUVmax in the tumours was greater (1.85 ± 1.22) than that in the normal breasts (0.59 ± 0.37). [^89^Zr]Zr-N-sucDf-bevacizumab is now undergoing a clinical trial for PET/CT imaging in neurofibromatosis type 2 (NF2) patients (NCT05685836). Zhang et al. developed a copper-64-based radiotracer by combining tetrazine with bevacizumab, and used pre-targeted immunoPET near-infrared fluorescence for imaging.^256^ This tracer can be used to image colorectal cancer (CRC) tumours with VEGF overexpression. In addition to bevacizumab, ranibizumab can also be radiolabelled as an immunoPET tracer. Luo et al. used [^64^Cu]Cu-NOTA-RamAb, a PET/CT imaging agent based on ramucirumab, to map the expression of VEGF-2 in vivo.^257^ The result showed specific and prominent uptake of [^64^Cu]Cu-NOTA-RamAb in VEGFR-2-positive HCC4006 tumours (9.4 ± 0.5%ID/g).

TMVP1 (LARGR) is a VEGFR-3-targeting peptide. Li et al. successfully labelled TMVP1 with gallium-68 and demonstrated that the radiotracer can be rapidly cleared at 30–60 min post-injection in preclinical models. In another study, [^68^Ga]Ga-DOTA-TMVP1 PET/CT detected 8 out of 10 metastatic lesions in all 5 patients with recurrent ovarian cancer.^258^ In addition to targeted imaging of tumour angiogenesis, many studies have focused on targeted imaging of angiogenesis in animal models of induced ischaemia. In a rat model of myocardial infarction (MI), targeted PET/CT imaging with [^64^Cu]Cu-DOTA-VEGF121 demonstrated enhanced angiogenesis with increased signalling within the infarcted area.^259^

Although several VEGFR-targeting radiopharmaceuticals have been developed, most of them are still based on antibodies with large molecular weights. In the near future, the development of targeting molecules with smaller size, such as nanobodies and small molecule drugs, will become a new trend in VEGFR-targeting candidates development. These smaller molecules have the potential to have higher affinity and specificity, providing better options for visible VEGFR-targeting radiopharmaceuticals.

### Immune-related targets

**Fig. 7:**
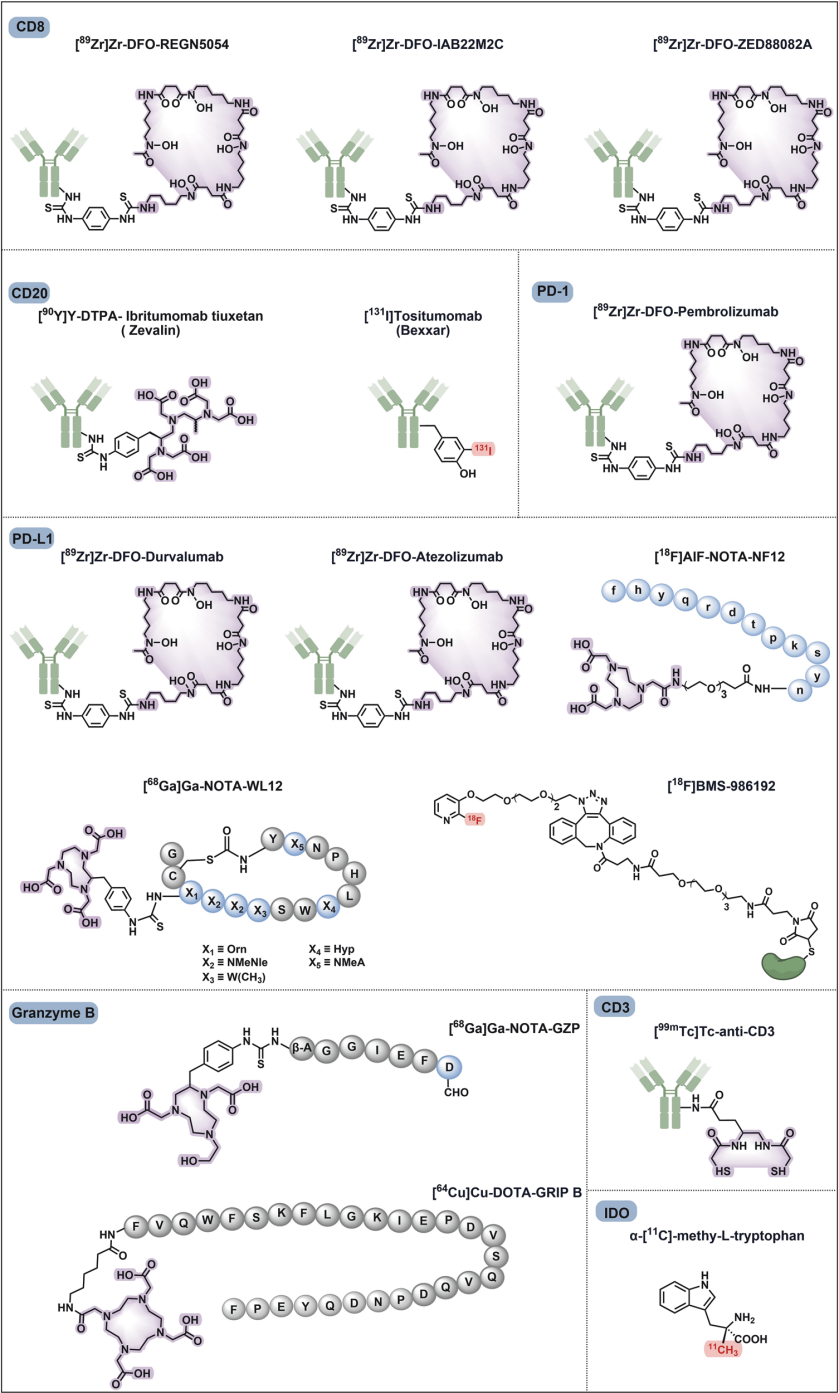
Chemical structures of clinically evaluated radiopharmaceuticals involving immune-related targets. Representative clinically evaluated immune-related radiopharmaceuticals, such as CD8, CD3, CD20, PD-1, PD-L1, IDO and Granzyme B, CD20 and PD-L1/PD-1 are well-established in clinical settings. Two CD20-targeting radiopharmaceuticals have been approved ([^90^Y]Y-DTPA-Ibritumoma btiuxetan and [^131^I]Tositumomab), while emerging targets such as CD8 and Granzyme B are gaining attraction for their potential for clinical translation. Although CD3 and IDO have fewer clinical applications, they represent promising areas for future exploration. Antibody-based radiopharmaceuticals are highly specific but can present challenges in terms of PK and tumour penetration, while peptide-based drugs provide faster clearance and better tissue penetration and are ideal for imaging. Small molecules, while cleared quickly, require careful design to ensure specificity. Grey circles: natural amino acids; blue circles: unnatural amino acids; highlighting in red: labelling with fluorine-18, carbon-11, or iodine-131; highlighting in purple: chelator for metal radionuclide labelling

Growing evidence strongly suggests that the tumour immune microenvironment plays a crucial role in tumour immune surveillance and immunological evasion. Tumour immunotherapy has been established as the standard treatment option for a range of malignancies.^260–264^ However, increasing numbers of clinical practices have shown that patients’ benefits from immunotherapy are limited due to the heterogeneity of immune-related target expression. Therefore, monitoring expression in the immune status of cancer patients is necessary to preselect patients who will benefit from immunotherapy.^265,266^ Radiopharmaceutical-based PET/CT or SPECT/CT imaging provides an ideal modality for rapid, accurate dynamic, and noninvasive monitoring of the tumour immune status,^267^ enabling the immune profiling of patients before treatment and predicting the effectiveness of immunotherapy. Additionally, investigating the immune environment in autoimmune diseases and inflammation is highly important. In autoimmune diseases, PET/CT imaging can visualize inflammation and immune responses to drugs at affected sites, allowing the diagnosis and monitoring of disease progression. In various inflammatory diseases, PET/CT imaging can reveal the status of inflammation, providing guidance for clinical intervention. In this section, we focus on the use of immuno-PET/CT imaging to characterize clinically relevant molecular targets, including general T-cell markers (such as CD3, CD4, and CD8) and immune checkpoints (such as PD-1, PD-L1, and CTLA-4).^268,269^ We also present the chemical structures of clinically evaluated radiopharmaceuticals involving tumour immune-related targets (Fig. 7).

#### Cluster of differentiation 8 (CD8)

CD8 is a transmembrane glycoprotein that functions as a co-receptor for the T-cell receptor (TCR), consisting of a CD8-α chain and a CD8-β chain. It primarily defines cytotoxic effector cells and may also delineate subsets of natural killer and regulatory T cells. CD8 plays a crucial role in facilitating the interaction between the TCR and class I major histocompatibility complex (MHC) molecules. Tumour-infiltrating lymphocytes (TILs) have revealed the critical role of the tumour immune microenvironment in the progression and treatment of cancer. Specifically, cytotoxic CD8^+^ T cells at tumour sites have been identified as a predictive marker for overall patient survival in various cancers, including BC, lung cancer, ovarian cancer, melanoma, and colorectal cancer.^270–272^ Consequently, the noninvasive characterization of both systemic and tumour-infiltrating CD8^+^ T lymphocytes is critical for patients undergoing cancer immunotherapy. This approach provides crucial insights into the immune landscape of both the tumour and the whole body, thereby enabling the development of more tailored and effective treatment strategies.

Tavaré et al. conducted preclinical studies of [^89^Zr]Zr-DFO-REGN5054, demonstrating that the tracer specifically targets T cells-rich lymphoid tissues of VelociT mice. And they developed two in vivo tumour models, which differ in the number of T-cells present systemically outside of the tumour, to detect therapy-induced changes in CD8^+^ lymphocytes. In the Raji/hPBMC/NSG co-implant model treated with REGN1979 (0.1 mg/kg), the PET/CT imaging at six days post-injection of this tracer showed that the uptake of the tumour (ROI: 35.5%ID/g) was higher than the control group (ROI: 18.9%ID/g). In the Raji/hPBMC/SRG-15 model treated with REGN1979 (0.5 mg/kg), PET/CT imaging exhibited that the value of tumour uptake (ROI: 27.2%ID/g) was much higher than control group (ROI: 7.3%ID/g). Those demonstrated systemic CD8 expression can impact the distribution and tumour uptake of [^89^Zr]Zr-DFO-REGN5054. These models demonstrate the utility of [^89^Zr]Zr-DFO-REGN5054 to detect and quantify changes in both systemic and intratumorally CD8 + T cells. Supported by the results of preclinical trials, they initiated clinical trials to monitor T-cell responses in patients receiving cancer immunotherapy. They focused on using [^89^Zr]Zr-DFO-REGN5054 in patients with solid malignancies treated with cemiplimab (NCT05259709).^273^ These clinical trials are ongoing, and there is no relevant result reported yet. IAB22M2C is a humanized 80-kDa minibody engineered single-chain variable-fragment–heavy-chain 3 antibody fragment that specifically targets human CD8 T cells with high binding affinity. Griessinger et al. first performed in vitro and in vivo preclinical studies with [^89^Zr]Zr-DFO-IAB22M2C. Preclinical PET/CT imaging studies have shown that [^89^Zr]Zr-DFO-IAB22M2C can detect infiltrating CD8^+^ T cells across various mouse models. The CEA-TCB/CEA-4-1BBL combination therapy was used to treat MKN-45 human gastric cancer cells implanted in humanized mice, and treatment-related CD8^+^ T-cell infiltration was detected by [^89^Zr]Zr-DFO-IAB22M2C, which resulted in a higher uptake of 5.13 ± 0.30%ID/cc than the vehicle group and monotherapy group.^274,275^ Taskar et al. performed the first-in-human study of [^89^Zr]Zr-DFO-IAB22M2C in six subjects with metastatic solid tumours who received approximately 3 mCi of [^89^Zr]Zr-DFO-IAB22M2C, followed by PET/CT scans at approximately 1–2, 6–8, 24, 48, and 96–144 h post-injection. Higher and earlier [^89^Zr]Zr-DFO-IAB22M2C uptake was detected in tumour (SUV ranging from 5.85 to 22.8) and CD8-rich tissues. And the tracer infusion was well tolerated, with no side effects.^276^ The clinical evaluation of [^89^Zr]Zr-DFO-IAB22M2C showed that it was safe and had the potential to be used as a CD8 targeting PET tracer and to predict early response to immunotherapy which had entered a phase II clinical trial (NCT03802123). Additionally, Ruijter et al. revealed the CD8-specific antibody [^89^Zr]Zr-DFO-ZED88082A, which showed high uptake in tumours across different patients two days post-injection. They demonstrated that [^89^Zr]Zr-DFO-ZED88082A can effectively characterize the intricate behaviour of CD8^+^ T cells in the context of immune checkpoint blockade (ICB), thereby potentially guiding immunotherapy.^277^

Tracers targeting CD8^+^ T cells have the potential to be used as clinical tools to guide personalized treatment. However, at present, there are no CD8 targeting tracers have been approved. A number of clinical data show that immune organs have high nonspecific uptake, which impacts the image of lesions. High uptake was also detected in the liver because many CD8 PET tracers are based on antibodies. Consequently, larger CD8 PET/CT imaging datasets need to be generated and correlated with clinical outcomes to assess whether the accuracy of CD8 radiotracer-based therapeutic guidance.

#### Cluster of differentiation of 3 (CD3)

CD3, an essential biomarker present on the T-cell membrane, is composed of four unique polypeptide chains: ε, γ, δ, and ζ. These chains form three distinct pairs of dimers, namely εγ, εδ, and ζζ, which together constitute the complex structure of the CD3 molecule.^278,279^ The CD3 complex functions as a co-receptor on T cells and noncovalently connects with the TCR to form the TCR/CD3 complex. This complex is essential for T-cell development, ensuring the maturation of T cells, and which are equipped to participate in the immune response. This interaction between CD3 and TCRs is fundamental to the ability of the adaptive immune system to detect and respond to antigens.^280^ The CD3 antigen is found on more than 95% of circulating T cells in human peripheral blood and activated T cells in inflammatory tissues. The presence of CD3 is a key marker for identifying T cells in both healthy and disease states, thus highlighting its importance in immune surveillance and response.

Visilizumab (Nuvion®) is a humanized anti-CD3 mAb that specifically binds to the CD3ε chain of the TCR, which is predominantly expressed on activated T cells.^281^ In 2009, Malviya et al. proposed that [^99m^Tc]Tc-SHNH-visilizumab could be used as a tracer for imaging T-cell trafficking and lymphocytic infiltration in tissues and organs affected by autoimmune diseases. They evaluated [^99m^Tc]Tc-SHNH-visilizumab in vitro and in vivo. The specific uptake of [^99m^Tc]Tc-SHNH-visilizumab showed that the T/B ratio increased with the number of HuT78 cells (CD3-positive) injected at both 6 and 24 h.^282^ In 2010, Flávia et al. used [^99m^Tc]Tc-anti-CD3 scintigraphy for the differential diagnosis of rheumatic diseases. Their study involved a clinical evaluation of 77 patients suffering from various rheumatic conditions. SPECT/CT scans indicated significant joint uptake of the tracer in joints from patients with rheumatoid arthritis (RA) and juvenile idiopathic arthritis (JIA). Moreover, this uptake progressively increased in later images. Conversely, in cases of gouty arthritis, joint uptake decreased in later scans. This different pattern of joints uptake enables to distinct these rheumatic diseases, thus highlighting the potential of [^99m^Tc]Tc-anti-CD3 scintigraphy as a valuable diagnostic tool in the clinical diagnosis of rheumatic disease.^283^ Another study used [^89^Zr]Zr-DFO-CD3 antibodies in a murine tumour xenograft model to study the response of anti-CTLA-4 immunotherapy on colon cancer, and revealed that high uptake in mice treated with anti-CTLA-4 correlated with a subsequent reduction in tumour volume. This correlation suggests that CD3-targeting radiotracers can serve as potential diagnostic tools for predicting patient response to anti-CTLA-4 immunotherapy.^268^

Although CD3 PET/CT imaging results strongly predict immune responses in tumours treated with ICB and in autoimmune diseases, further work is needed to validate their potential for clinical application. Future work may look at PK optimization using smaller carriers such as peptides, small molecules, or other smaller biological constructs to optimize the retention time for imaging and improve specific uptake.

#### Cluster of differentiation 4 (CD4)

CD4, is a transmembrane glycoprotein that functions as a co-receptor for the TCR. The CD4 protein contains 458 amino acids with a molecular weight of approximately 55 kDa. It is a single-chain molecule comprising four immunoglobulin-like structural domains. It binds to a conserved site on the structural domain of the β2 domain of a class II MHC molecule.^284^ CD4 is expressed predominantly on the surface of T lymphocytes and serves as a molecular marker of the TCR. CD4 plays an important role in regulating the immune response, mediating downstream signalling through the secretion of specific cytokines, and activating the expression of transcription factors, thereby promoting T-cell activation. To date, five major CD4^+^ T helper cell subpopulations have been identified: Th1, Th2, Th17, Treg, and Tfh cells.^285,286^ The CD4 protein is one of the main receptors for HIV, and HIV infection invades CD4^+^ T cells mainly by binding to the CD4 protein.^287^ The determination of CD4^+^ T-cell counts can help monitor the immune status of HIV-infected patients. Additionally, the application of CD4^+^ T cells in immunotherapy can be expanded to include CAR-T cell therapy.^288^ Therefore, the molecular imaging of systemic CD4^+^ T cells is important for monitoring autoimmune diseases and cancer immunotherapy.

The gut is an important component of the immune system. The dysregulation of the immune response between microbial-derived antigens and the immune response to pathogens in the gut can lead to human inflammatory bowel disease (IBD), which is partly characterized by an abnormal CD4^+^ T-cell response. Currently, the identification of inflammatory lesions in the gut requires invasive procedures such as biopsy and colonoscopy. In 2010, Hindryckx et al. used [^18^F]FDG to monitor the common used dextrose sodium sulphate (DSS)-induced mouse model of IBD.^289^ However, the physiological uptake of [^18^F]FDG in the gut is highly variable, limiting its application in IBD.^290^ In 2018, Freise et al. developed [^89^Zr]Zr-DFO-GK1.5 cDb, a fragment of an anti-mouse CD4 antibody derived from the GK1.5 hybridoma. This PET tracer was used to visualize the distribution of CD4^+^ T cells in the abdominal region and lymphoid organs of mice with DSS-induced colitis. PET/CT imaging revealed increased uptake of CD4^+^ T cells in the colon, caecum, and mesenteric lymph nodes of the mice with colitis. This finding serves as a crucial guideline for further research on CD4^+^ T cells in preclinical models of IBD.^291^

Studies of CD4 targeting PET tracers have shown that their use in inflammation is more prominent and that they can stratify sites of inflammation compared to [^18^F]FDG. Therefore, many clinical data are needed to support the use of CD4 in inflammatory scoring in future studies.

#### Cluster of differentiation 20 (CD20)

CD20, a transmembrane protein found on the surface of B cells, is expressed mainly during the early developmental and maturation stages of B cells, and plays a crucial role in the activation, proliferation, and differentiation of B cells. CD20 is present on more than 95% of B-cell malignancies; however, it cannot be found on hematopoietic stem cells, plasma cells or other normal tissues, thus making CD20 a tumour-specific target.^292^ Therefore, plenty of attempts have focused on the design and generation of specific antibodies that bind to CD20. CD20 antibodies represent one of the most effective antitumour therapeutic strategies currently available,^293^ and rituximab, ofatumumab, obinutuzumab and ublituximab were approved for treating chronic lymphocytic leukaemia in the USA in 1997 and 2010 and in Europe in 2014.

Given the rapid progress of radioimmunotherapy, new treatment options are emerging for patients with lymphoid cancers. There are two approved CD20 targeting radiopharmaceuticals and a series of radiopharmaceuticals at the preclinical stage. In 2002, the FDA approved [^90^Y]Y-DTPA- Ibritumomab tiuxetan (Zevalin®) as a new therapeutic modality for patients with relapsed and refractory low-grade or follicular B-cell non-Hodgkin’s lymphoma (NHL).^294,295^ In 2003, the FDA approved another anti-CD20 antibody, namely, [^131^I]Tositumomab (Bexxar®), for treating patients with relapsed or refractory NHL.^296,297^ Jauw et al. performed a clinic study in which [^89^Zr]Zr-DFO-rituximab was used to assess CD20-targeting in 6 patients with relapsed/refractory diffuse large B cell lymphoma. In only five patients, tracer uptake in the tumour was consistent with CD20 expression. Three patients showed tumour uptake of [^89^Zr]Zr-DFO-rituximab (SUVpeak = 3.2-5.4), and one patient demonstrated intense tumour uptake (SUVpeak = 12.8), which was consistently positive for CD20 expression determed by IHC.^298^ Although [^89^Zr]Zr-DFO-rituximab shows some clinical potential, additional research is needed to determine whether tumour uptake correlates with clinical benefits in patients treated with rituximab, which could be achieved by [^89^Zr]Zr-DFO-rituximab PET for guiding personalized treatment strategies. Like rituximab, [^131^I]rituximab has been used for treating patients with aggressive CD20-expressing B-cell NHL.^299^

Although CD20 targeting radiopharmaceuticals are the most rapidly developing CD family, they have shown limited sales and clinical use for several reasons. Intact antibodies have slow blood clearance and high uptake in the liver. The peak tumour uptake is observed for several days after injection, for mAb which delay the diagnsitic.^300^ Hence, the focus of targeting should be shifted to vector discovery with small efficiency, such as peptides and small molecules.

#### Cluster of differentiation 30 (CD30)

CD30, a member of the tumour necrosis factor (TNF) receptor family, is a type I transmembrane protein. It contains three main structural domains: an intracellular structural domain, a transmembrane domain, and a cysteine-rich extracellular structural domain. CD30 is expressed on activated T cells (both CD4^+^ and CD8^+^) and a subset of B cells but not on quiescent T cells. Typically, CD30 is not expressed in normal human tissue. In contrast, its expression is higher in disease states such as viral infections, autoimmune diseases and various lymphomas.^301^ A review of more than 1200 cases by the German Hodgkin Study Group (GHSG) revealed that 98.4% of classical Hodgkin’s lymphomas were positive for CD30, thus making it an important biomarker for lymphohaematopoietic malignancies.^302^

Rylova et al. utilized [^89^Zr]Zr-DFO-AC-10 antibodies for detecting CD30-positive human lymphomas and demonstrated their potential to effectively target and visualize CD30 expression in lymphomas within these human Karpas 299 tumours (CD30-positive model) or A-431 tumours (CD30-negative model), with the highest uptake in CD30-positive tumours (ROI: 37.9 ± 8.2%ID/g), 72 h post-injection. This significant uptake supports the use of [^89^Zr]Zr-DFO-AC-10, which could be instrumental in selecting patients for brentuximab vedotin (BV) treatment, an approved CD30-specific ADC, monitoring clinical responses to tailor therapy effectively, and reducing the toxicity of BV. This targeted approach allows for more precise and personalized treatment strategies for managing CD30-positive lymphomas.^303^ Therefore, Kang et al. used [^89^Zr]Zr-DFO-BV for noninvasive imaging of CD30 expression in different lung cancer models. The results confirmed that the tracer accumulation was highest in the H460 tumour model, reaching 9.93 ± 2.70%ID/g at 24 h post-injection. Therefore, [^89^Zr]Zr-DFO-BV was also reported to be a promising agent for the noninvasive determination of CD30 expression in lung cancer.^304^ Dietlein et al. compared new anti-CD30 radioimmunoconjugates of vary structures radiolablled with iodine-131 in Hodgkin lymphoma xenografts. In vivo, tumour accumulation results showed promising potency of the PET tracer, with 2.6%ID/g for [^131^I]-5F11 and 12.3%ID/g for [^131^I]Ki-4.^305^ Schnell et al. used [^131^I]Ki-4 to treat 22 patients with relapsed or refractory CD30-positive refractory Hodgkin’s lymphoma. [^131^I]Ki-4 achieved an OR in 27% of patients but induced severe haematologic toxicity in 33% of patients.^306^

Some adverse effects have also been observed for the existing CD30-targeting radiopharmaceuticals. Hence, the optimization of CD30-targeting vectors and labelling techniques are required to increase the stability and targeting ability of the drugs in vivo, which could improve their therapeutic efficacy and safety.

#### Programmed death 1 (PD-1)

PD-1 is a type I transmembrane protein comprising 288 amino acids and belongs to the B7 receptor immunoglobulin family, which includes three domains: an immunoglobulin superfamily domain, a transmembrane domain, and an intracellular domain.^307^ Notably, PD-1 is highly expressed on tumour-specific T cells, indicating its critical role in the immune response to cancer.^308^ The interaction between the PD-1 pathway and its ligand PD-L1 is crucial for suppressing anti-tumour immune responses, which represents a substantial breakthrough in cancer therapy. However, not all patients respond to ICB, thus highlighting the need to preselect patients with high tumour PD-1 expression. Therefore, it is necessary to develop diagnostic and prognostic imaging tools that can be used to evaluate PD-1 status in vivo. Currently, the most suitable method is immune-PET/CT imaging, which can be used as a promising non-invasive method for detecting and quantifying the presence of PD-1 expressing TILs in the TME. This approach can facilitate the screening of cancer patients who are more likely to respond to anti-PD-1 immunotherapy, thereby optimizing treatment plans and enhancing patient outcomes.

Pembrolizumab is an antibody known as ICB that specifically binds to the PD-1 on the surface of T cells. This binding inhibits the PD-1, thereby enhancing the immune response against cancer cells.^309^ With respect to pembrolizumab, Natarajan et al. performed a series of immuno-PET preclinical experiments. They evaluated [^64^Cu]Cu-DOTA-pembrolizumab in two different mouse tumour models, one bearing hPD-1-expressing 293 T stable cell line xenograft (NSG/293 T/hPD-1) and the other bearing A375 tumour that does not express hPD-1 but in which some infiltrating TILs do express hPD-1 (hNSG/A375), to observe tracer retention time in clearance organs and predict human equivalents dosimetry. The blocking experiments demonstrated that this tracer specifically targeted in NSG/293 T/hPD-1 (the tumour uptake of blocking group vs. non-blocking group: 5.5 ± 0.1%ID/g vs. 14.8 ± 1.2%ID/g (p = 0.005) at 48 h. The tracer’s predicted annual human dose is well within the FDA-accepted limits.^310,311^ Kok et.al. used [^89^Zr]Zr-DFO-pembrolizumab to assess the clinical response to PD-1 blockade in patients with cancer. The results revealed that patients with advanced or metastatic melanoma or NSCLC who subsequently received PD-1 antibody treatment presented similar tumour SUVmax values (SUVmax=4.9 and 6.5, respectively; P = 0.49). They demonstrated that [^89^Zr]Zr-DFO-pembrolizumab is safe and that its tumour uptake is correlated with ICB treatment response and patient survival.^312^

Few PET tracers have been developed for PD-1, as targeting PD-L1 offers more advantages. Compared with PD-L1, PD-1 is expressed mainly in immune cells, and because of the wide distribution of immune cells, tracers targeting PD-1 may not provide high-contrast tumour imaging, affecting diagnostic accuracy. In future research, the development of PET tracers for PD-1 will need to focus on highly specific molecular designs to ensure that they can effectively bind tumour PD-1.

#### Programmed death ligand 1(PD-L1)

As mentioned above, the PD-1/PD-L1 interaction is critically involved in the immune evasion mechanisms of tumour cells. Since PD-L1 is expressed on the surface of tumour cells, the study of PD-L1, rather than its receptor, is far more promising. PD-L1 is widely overexpressed in various tumours.^313^ This broad expression profile highlights its importance as a key factor in the immune evasion strategies of diverse cancer types and reveals its potential as a universal target for immunotherapy.^262^ PD-L1 expression is predominantly determined by IHC analysis of tissue samples, a process that requires invasive biopsy methods. However, PD-L1 expression is known to be heterogeneous and can vary substantially over time. This dynamic nature of PD-L1 expression poses challenges for accurate assessment of the response to immunotherapy. Consequently, the traditional method limits the ability to fully evaluate therapy-induced alterations in PD-L1 expression in real time.^314^ By combining PET/CT imaging and immunotherapy, noninvasive quantification of total PD-L1 expression in all lesions concurrently has the potential to enhance the accuracy of therapeutic decision-making by overcoming these limitations.^262,315^

To date, several preclinical PET studies have been conducted using antibodies, peptides, and small molecules to quantify PD-L1 expression in tumours during immunotherapy.^9,316–318^ Josefsson et al. labelled anti–PD-L1 antibodies with indium-111 for SPECT/CT imaging and in vivo biodistribution studies. In vitro evaluation of [^111^In]In-DTPA-anti-PD-L1 showed a high affinity to PD-L1 (KD = 8.3 ± 3.2 nM). The SPECT imaging demonstrated that the uptake of [^111^In]In-DTPA-anti–PD-L1 antibodies in tumour increased to 29.5 ± 7.4%ID/g at 24 h, reached the pick value of 56.5 ± 16.7%ID/g at 72 h post-injection.^319^ Maute et al. developed an engineered nanobody (HAC–PD-1) for PET imaging. [^64^Cu]Cu–DOTA–HAC–PD-1, in PD-L1–negative tumours or hPD-L1–positive tumours blocked by unlabelled HAC–PD-1, lacked uptake signals which indicated a high degree of specificity of [^64^Cu]Cu–DOTA–HAC–PD-1 for PD-L1 binding.^320^ By using multiple PD-L1 inhibitors approved for marketing by the FDA, researchers have subsequently developed new radiopharmaceuticals based on approved drugs that are already in the clinical stage. These include (1) [^18^F]BMS-986192 PET/CT imaging in advanced-stage non–small cell lung cancer (EUDRACT 2015-004760-11);^321^ (2) [^89^Zr]Zr-DFO-Durvalumab imaging in non-small cell lung cancer,^322^ and (3) [^89^Zr]Zr-DFO-Atezolizumab imaging in patients with locally advanced or metastatic renal cell carcinoma (NCT04006522).^323^ In addition to antibodies and small molecules, many effective peptide-based radiopharmaceuticals have emerged as suitable choice in recent years. Lesniak et al. reported a novel PD-L1 targeted peptide, namely WL12, which has a high binding affinity for human PD-L1. PET/CT imaging demonstrated a significantly higher accumulation of [^18^F]AlF-NOTA-WL12 in hPD-L1 tumours than in CHO controls. These findings indicate the potential of this radiopharmaceutical for assessment of PD-L1 expression in vivo.^324^ Ravindra et al. compared [^68^Ga]Ga-NOTA-WL12 with [^64^Cu]Cu-NOTA-WL12, both tracers showed similar uptake (approximately 16%ID/g) in hPD-L1 tumours at 60 min post-injection. [^68^Ga]Ga-NOTA-WL12 showed low uptake in the liver and muscle than did [^64^Cu]Cu-NOTA-WL12.^325^ Together, these findings indicate that [^68^Ga]Ga-NOTA-WL12 can provide appropriate contrast for swift imaging of changes in PD-L1 expression in tumours. Zhou et al. modified the chelator with NOTA and conducted a first-in-human evaluation of [^68^Ga]Ga-NOTA-WL12 in NSCLC patients(*n* = 9). The results revealed that in patients with high PD-L1 expression, the tumour uptake of [^68^Ga]Ga-NOTA-WL12 was higher (SUVmax: 4.87) than that in patients with low PD-L1 expression (SUVmax: 1.84).^326^ Another D-peptide antagonist radiopharmaceutical, [^18^F]AlF-NOTA-NF12, was reported by Zhou and his colleagues. The uptake of [^18^F]AlF-NOTA-NF12 in MC38 tumour-bearing mice was 5.04 ± 0.29%ID/g at 30 min, revealing its high PD-L1 specificity and safety. They studied 7 patients and reported that NSCLC patients with high PD-L1 expression had greater tumour uptake of [^18^F]AlF-NOTA-NF12 (SUVmax: 3.29, SUVmean: 2.75) than did those with low PD-L1 expression (SUVmax: 2.22, and SUVmean: 1.75). There was also a similar trend among patients with oesophageal cancer. These results provide sufficient data for its entry into clinical studies.^327^

We reviewed a range of PD-L1 targeting radiopharmaceuticals that are currently in preclinical and clinical research. There are still several challenges, such as low expression of PD-L1 and normal tissue expression of PD-L1. This may have an off-target effect, resulting in a low TBR. Therefore, it is necessary to develop more specific molecules for use in radiopharmaceuticals. These radiopharmaceuticals may soon transform the application of ICB-based therapies to enhance patient benefit rates. It is hoped that more radiopharmaceuticals that can help predict ICB patients in the early stage for efficient personalized therapy, benefiting more patients, will be developed.

#### Indoleamine 2,3-dioxygenase (IDO)

IDO is a hemoglobinase that degrades tryptophan (Trp), a necessary amino acid, through the kynurenine (Kyn) pathway (KP).^328^ IDO is crucial for sufficient levels of kynurenine, which is necessary for the proper proper functioning of human tissues, such as endothelial cells, epithelial cells, and mature DCs.^329^ Acquired immunological tolerance is associated with IDO activity, which can induce Treg activation and inhibit T-cell activation, thus enabling tumour cells to evade immune surveillance.^330^

In 2009, Juhász et al. conducted a study on 10 patients with lung or mediastinal tumours and performed PET/CT to evaluate the kinetics of α-[^11^C]-methyl-L-tryptophan ([^11^C]AMT) in vivo. The results revealed that high IDO activity could be identified by [^11^C]AMT.^331^ In 2012, Juhász et al. performed a dynamic PET/CT scan with [^11^C]AMT in nine women with BC (stages II-IV); the results revealed that IDO-positive BC (stages II-IV) exhibited rapid [^11^C]AMT uptake within 20 min post-injection.^332^ In 2016, Xin et al. synthesized two radiopharmaceuticals, namely 1-L-[^18^F]FETrp and 1-D-[^18^F]FETrp, for evaluating tryptophan metabolism mediated by the IDO-KP in tumours. The authors conducted a small animal PET/CT imaging study and reported that the in vivo tumour uptakes of 1-L-[^18^F]FETrp and 1-D-[^18^F]FETrp were 4.6 ± 0.4 and 1.0 ± 0.2%ID/g, respectively.^333^ Moreover, because 1-L-[^18^F]FETrp results in greater tumour uptake than 1-D-[^18^F]FETrp, Xin et al. demonstrated that 1-L-[^18^F]FETrp highly accumulates in gliomas, pancreatic cancer, and lung cancer, thus indicating that it is a potential radiopharmaceutical for imaging cancers with high IDO expression.^334^ Huang et al. synthesized a novel radiopharmaceutical, [^18^F]IDO49, for IDO-targeting PET/CT imaging in HeLa tumour-bearing mice; the results showed that [^18^F]IDO49 specifically accumulated in IDO1-expressing tumours.^335^ In 2017, Giglio et al. developed 5-[^18^F]F-AMT by directly introducing fluorine-18 into the aromatic ring of tryptophan. This tracer was used for IDO1-targeting PET/CT imaging in B16F10 melanoma mouse models, and their study unequivocally showed that ^18^F could be added directly to the Trp backbone at position 5, including the 1-methyl or α-methyl Trp.^336^

Among the IDO-targeting radiopharmaceuticals listed, [^11^C]AMT has been widely used for imaging of multiple types of cancer (such as brain tumours and metastatic breast cancer) in the clinic. However, the shorter half-life of carbon-11 limits its application. To overcome this challenge, many tryptophan analogues labelled with fluorine-18 have been developed, including 1-L-[^18^F]FETrp and 1-D-[^18^F]FETrp, as mentioned above. There are still challenges for improving the specificity and high tumour-uptake of IDO-targeting tracers for accurate tumour diagnosis.

#### Granzyme B

Granzyme B is a member of the serine protease family, and it is released by activated CD8^+^ T cells and NK cells for kill target cells.^337^ The visualization of granzymes B might therefore facilitate the assessment of the potential response to immunotherapy.^338^ In 2017, Larimer et al. designed and synthesized [^68^Ga]Ga-NOTA-GZP to predict the response of CT26 tumour-bearing mice to cancer immunotherapy.^339^ The authors reported that granzyme B could serve as a quantitatively valuable predictive biomarker for the assessment of cancer immunotherapy response.^340^ In a phase I study, the safety of [^68^Ga]Ga-NOTA-GZP was examined in 2019 (NCT 04169321) after its first-in-human research. In 2021, Zhao and coworkers developed [^64^Cu]Cu-GRIP B to detect in vivo granzyme B expression, suggesting that it may play a significant role in evaluating the early treatment response to immunotherapies.^341^ Currently, [^64^Cu]Cu-GRIP B is undergoing a phase II trial in the United States (NCT 05888532). In the future, the treatment of many diseases may benefit from the successful application of granzyme B imaging to tumours. Targeting granzyme B, which is overexpressed in tumours or inflammatory regions, is promising for imaging the atherosclerotic diseases, systemic lupus erythematosus.

#### Inducible T-cell costimulator (ICOS)

ICOS is defined as a homodimeric protein located on the surface of T cells and is expressed following TCR stimulation.^342^ ICOS is a major member of the CD28 superfamily, and it shares many similarities with CD28 in terms of structure and function.^343^ The primary difference between the two proteins is that ICOS cannot be expressed constitutively on resting T cells. A previous study used ICOS-targeting PET/CT imaging to detect ICOS expression and evaluate T-cell activity.^344^ In 2020, Xiao et al. used [^89^Zr]Zr-DFO-ICOS mAb for PET/CT scanning of Lewis lung cancer models; the authors demonstrated that ICOS expression indicates an immune response linked with T cells and confirmed that ICOS immunoPET is a potential method for monitoring, contrasting, and predicting the effectiveness of immunotherapy in cancer patients.^345^ In 2021, Simonetta et al. used the [^89^Zr]Zr-DFO-ICOS mAb to monitor CAR-T-cell therapy in a B-cell lymphoma mouse model. The authors showed that the use of immunoPET targeting as an endogenous biomarker could enable in vivo evaluation of CAR-T-cell dynamics and that this strategy could be useful in the clinical context for assessing the PK/PD of any investigational and commercially available CAR-T-cell products.^346^ Furthermore, Xiao and colleagues reported that the [^89^Zr]Zr-DFO-ICOS mAb could be an effective tool for the early diagnosis of graft-versus-host disease (GvHD) before the occurrence of clinical symptoms.^347^ In 2023, Alsaid et al. designed and synthesized [^89^Zr]Zr-IAB42M1-14 to evaluate the dynamics of CD8^+^ T-cell infiltration into lymphoid and tumour tissues after receiving antibody therapy that blocked PD-1 and ICOS.^348^ For targeted-ICOS imaging, challenges exist including normal tissue toxicity and low tumour specificity even though the two antibodies based radiotracers tracers emerged. Considerations must be taken when balancing the efficacy and toxicity of ICOS targeted PET tracer discovery.

#### TNF Receptor Superfamily Member 4 (OX40)

OX40, also known as CD134 or TNFSF4, is a member of the tumour necrosis factor receptor superfamily. It is predominantly expressed on the cell membrane of activated CD4^+^ and CD8^+^ T cells whereas OX40L is mainly expressed in antigen-presenting cells. The binding of OX40 with its ligand OX40L can activate CD8^+^ T cells.^344^ OX40 and OX40L are critical immune checkpoints in cancer cells. The binding of OX40 to OX40L reduces the immunosuppressive effect of Treg cells and induces the proliferation of T cells, thereby enhancing the immune response to specific antigens.^349^ Although many radiopharmaceuticals have been developed against this target and have shown good results, none of them has yet reached the clinical trial stage.

Alam et al. developed [^64^Cu]Cu-DOTA-AbOX40, a noninvasive PET/CT imaging tracer, which indicates T-cell activation in the entire body after treatment with the local CpG oligonucleotides (immunologic adjuvants) in tumour cells. The authors used A20 cells to establish a subcutaneous double-homogeneous tumour model in mice, in which CpG was administered in only one tumour. Two days after in situ treatment with CpG, PET/CT imaging revealed a specific OX40 expression along with T-cell activation. Compared with the untreated tumours, the tumours treated with CpG presented a strong PET signal. PET/CT revealed significant enhancement of the tracer in the spleen 9 days after CpG treatment. This radiopharmaceutical is a promising candidate for monitoring tumours immunotherapy in the clinic.^350^ Simonetta et al. developed [^64^Cu]Cu-DOTA-mAbOX40, which can act as an immunoPET tracer to achieve specific imaging of OX40^+^-activated T-cells in a mouse model of acute graft versus host disease (GVHD), which is a systemic disease characterized by multisystem damage (skin, oesophagus, gastrointestinal, liver, etc.) that occurs after bone marrow transplantation (BMT).^351^ Nobashi et al. developed an [^89^Zr]Zr-DFO-OX40 mAb to detect OX40^+^ activated T cells in a murine orthotopic glioma model. The [^89^Zr]Zr-DFO-OX40 mAb was able to monitor the therapeutic effect of dendritic cells in treating glioblastoma.^352^ OX40 imaging is promising for immunoPET/CT imaging. Some adverse effects (such as the exacerbation of GVHD after administration of a tracer) are observed despite its high accumulation in tumours. Novel targeting vectors with improved specificity and reduced toxicity are urgently needed for OX40 imaging.

### Neurological disease-directed drug targets

**Fig. 8:**
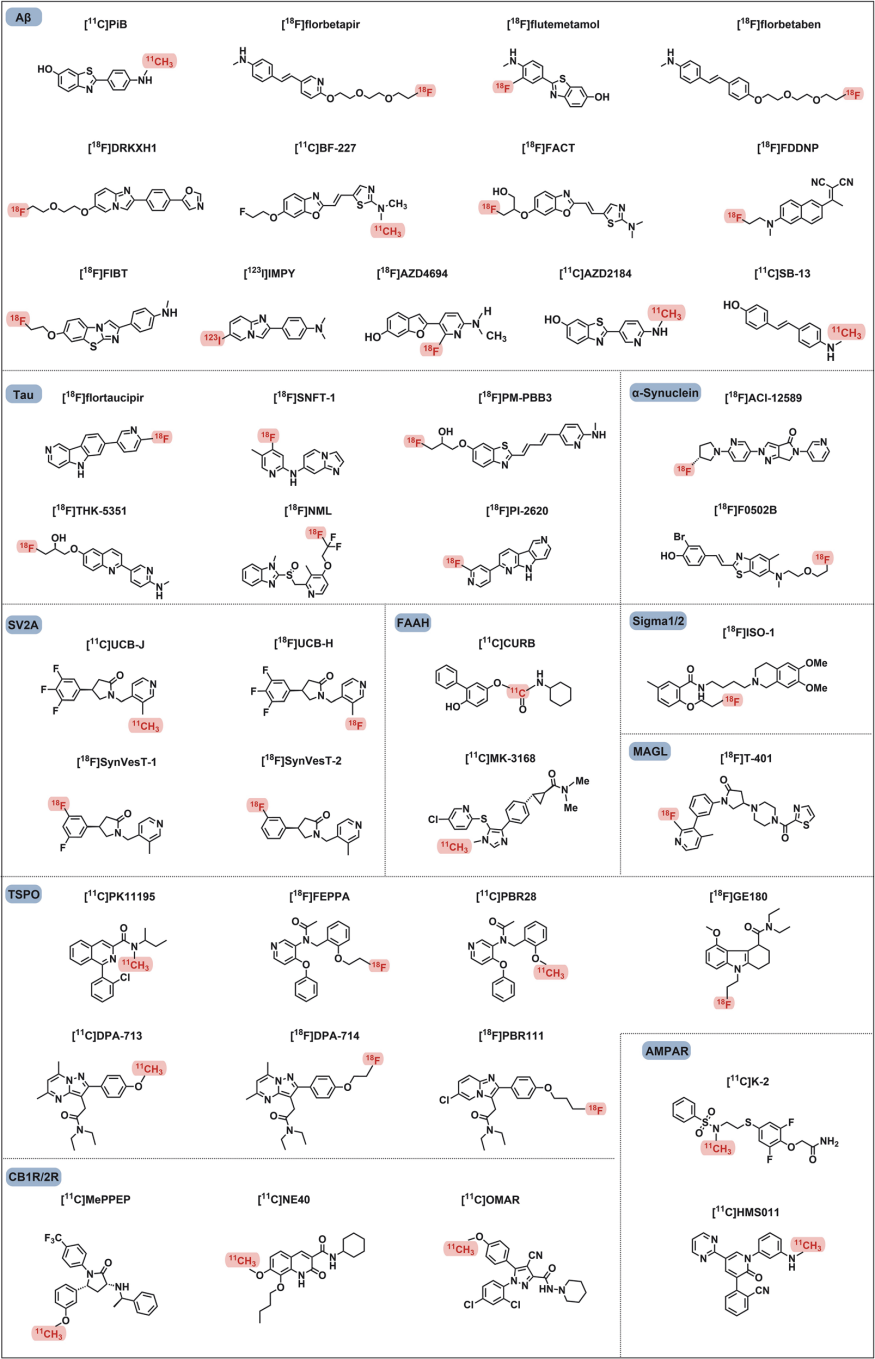
Chemical structures of clinically evaluated radiopharmaceuticals involved in neurodegenerative disorders. Representative clinically evaluated radiopharmaceuticals for neurodegenerative disorders. For AD diagnosis, the ^18^F-fluorinated radiotracers are gaining more focus with the approval of [^18^F]florbetapir, [^18^F]flutemetamol, [^18^F]florbetaben, and [^18^F]flortaucipir (Tau-targeting). Although [^11^C]PiB is mostly used in AD diagnosis, [^18^F]AZD4694 (Aβ-targeting) and [^18^F]PI-2620 (Tau-targeting) are most promising approved radiotracers. TSPO-targeting diagnosis has been well investigated in brain imaging. On the basis of [^11^C]PK11195, many radiopharmaceuticals have emerged and [^18^F]GE180 was investigated in phase II. For Sigma1/2, effective and promising imaging results with the discovery of [^18^F]ISO-1 and other high binding affinity tracers are under developed. Highlighting in red: labelling with fluorine-18 and carbon-11

Radiopharmaceuticals are also widely used for detecting abnormally overexpressed proteins in the nervous system. Neurodegenerative disorders, such as AD, adversely influence millions of people, causing impaired memory and cognition. Molecular imaging-based PET/CT scans offer unique opportunities for the early diagnosis of neurodegenerative disorders, achieving early intervention and controlled disease progression. The hallmark pathogenesis includes abnormal protein aggregation, synaptic and neuronal network dysfunction. The FDA-approved radiopharmaceuticals (such as [^18^F]fluorobetapir and [^18^F]flortaucipir) hold great promise for the clinical diagnosis of patients with neurodegenerative disorders. Aβ plaques, tau protein and α-synuclein aggregates are well-studied targets in AD diagnosis and therapy. In addition, radiopharmaceuticals have been widely investigated for the diagnosis of neuroinflammation. This chapter focuses on the current progress of radiopharmaceutical discovery for neurological diseases, and provides representative drug candidates for use in clinical studies and provides perspectives for the future development and clinical application of relevant radiopharmaceuticals. We also present the chemical structures of clinically evaluated radiopharmaceuticals involving neurological disease-directed drug targets (Fig. 8).

#### Amyloid β (Aβ)

AD is recognized as the most common neurodegenerative disorder. The abnormal aggregation of Aβ plaques in the brain is highly correlated with AD progression. Hyperphosphorylation of Aβ leads tonerve fibre degeneration and nerve cell apoptosis.^353^ The development of drugs that target Aβ plaques for early diagnosis and control of disease progression is a great scientific challenge. Gratefully, three ^18^F-fluorinated Aβ-targeting diagnostic agents have been approved. Consequently, based on these approved candidates, scientists are devoted to developing more specific radiopharmaceuticals for accurate diagnosis of AD. This section summarizes the disclosed Aβ-targeting radiotracers and focuses mainly on clinical investigations, especially those that are expected to be approved.

[^18^F]AZD4694 ([^18^F]NAV4694), [^18^F]FACT, [^18^F]FDDNP, and [^18^F]FIBT are ^18^F-fluorinated small molecules that have been used for imaging Aβ in clinical practice. [^18^F]AZD4694, which was developed by Rowe et al., is a promising radiopharmaceutical for Aβ in aging and dementia population. Researchers revealed that the SUV ratio of [^18^F]AZD4694 was strongly correlated with the diseases.^354^ In another clinical trial, 45 patients who underwent PET/CT imaging with [^11^C]PiB (the gold standard for Aβ imaging) and [^18^F]AZD4694 showed similar imaging performance.^355^ Presently, [^18^F]AZD4694 is being investigated in a phase III clinical trial in the USA. [^18^F]FACT, which is an analogue of [^11^C]BF-227(discussed below), can clearly differentiate AD patients from normal controls; however, their PKs and distribution patterns in the brain are substantially different.^356^ [^18^F]FDDNP, which binds to both Aβ and NFT, can distinguish AD patients from healthy controls in the elderly population and is more sensitive to cognitive decline than [^18^F]FDG.^357^ The [^18^F]FIBT was developed by Henriksen and Wester et al. for cerebral Aβ deposits.^358^ This tracer showed high affinity and specificity for Aβ and low binding affinity for α-synuclein aggregates. It also exhibited suitable uptake in the mouse brain as well as in the first-in-human study. A further study reported that [^18^F]FIBT showed comparable results to those of [^11^C]PiB, which supports its further investigation in larger human samples.^359^

[^123^I]IMPY, developed by Newberg et al., is a ^123^I-iodinated small molecule for diagnosing AD. In a clinical study, this radiopharmaceutical was tested to evaluate its imaging ability among nine patients between aged 44–80. This tracer was found to be effective and safe for identifying AD patients.^360^ [^125^I]DRK092 is a new ^125^I-iodinated imidazo[1,2-a]pyridine derivative developed by Chen et al. for Aβ imaging and has a similar structure to [^123^I]IMPY. An in vivo imaging study revealed that [^125^I]DRK092 is more specific and sensitive for Aβ accumulation detection than [^123^I]IMPY. This finding also confirmed a greater accumulation of this tracer in the cortical area enriched with Aβ plaques. However, the use of [^125^I]DRK092 in clinical applications is limited because of its lower resolution in SPECT scanning.^361^ To overcome this challenge, Wang et al. designed an ^18^F-fluorinated tracer, [^18^F]DRKXH1. A small animal PET/CT study displayed high uptake in Aβ plaques and rapid washout in the AD brain. The distribution volume ratio of [^18^F]DRKXH1 was greater than that of the approved tracer [^18^F]AV45.^362^

[^11^C]BF-227, [^11^C]AZD2184, and [^11^C]SB-13 are ^11^C-labelled PET tracers used for imaging dense Aβ deposits in AD patients. [^11^C]BF-227 was first reported by Hiroyuki Arai and was evaluated in 11 healthy individuals and 10 AD patients. Dynamic PET images revealed that [^11^C]BF-227 has a strong affinity for synthetic Aβ1-42 fibrils.^363^ [^11^C]AZD2184 can also identify AD patients. However, the undesired binding with white matter limits its further use.^364^ [^11^C]SB-13, developed by Kung et al., also demonstrated an in vivo effect comparable to that of [^11^C]PiB for the distinct diagnosis of AD patients from normal individuals.^365^ However, [^11^C]SB-13 also exhibited greater uptake in the brain of the control group.

Aβ-targeting imaging tracers for the diagnosis of AD have evolved for several decades. Carbon-11, fluorine-18 and iodine-123 labelled radiopharmaceuticals have been fully developed and used in clinical trials. Although [^11^C]PiB is the most commonly used imaging agent, ^18^F-fluorinated tracers have more potential in applications because of their suitable half-life. Radiotracers that show high specificity and binding affinity as well as rapid washout from the brain, will be more promising for Aβ imaging.

#### Tau

In AD patients, tau protein aggregation generally occurs after the appearance of Aβ plaques, and the amount of aggregation is significantly lower than that of Aβ plaques. There is strong evidence that tau is the main constituent of the paired helical filaments and a major hallmark of neurodegenerative disorders. Tau proteins are highly phosphorylated to form neurofibrillary tangles, along with senile plaques (SPs), which function as pathological markers of AD.^366,367^ However, there was no radiopharmaceutical demonstrating strong binding affinity and high specificity for the tau protein until the development of T807. Notably, the approval of [^18^F]flortaucipir (originally named T807) broke new ground in tau protein imaging for AD patients and was successful in clinical practice. Hopefully, scientists have made sustained efforts to develop novel tau-targeting radiopharmaceuticals and achieved great progress both preclinically and clinically. Most tau-targeting radiopharmaceuticals are ^18^F-fluorinated small molecules, such as [^18^F]SNFT-1, [^18^F]PM-PBB3, [^18^F]THK-5351, and [^18^F]MK-6240. The clinical progress of these molecules is described in detail here.

[^18^F]SNFT-1, which was optimized from imidazo[1,2-a]pyridine derivatives, bound strongly to tau and exhibited high initial brain uptake and rapid clearance from normal tissues. This tracer shows high selectivity and affinity only for the tau protein and does not bind to any other protein.^368^ [^18^F]PM-PBB3 can also be used for PET/CT imaging in neurodegenerative disorders. Su et al. conducted a PET/CT study of [^18^F]PM-PBB3 in 35 patients with frontotemporal dementia and revealed slight deposition of the tau protein in the cerebral lobes.^369^ Weng et al. noted that [^18^F]PM-PBB3 uptake in the cerebral cortex was significantly increased in 6-month-old mice and further increased in 9-month-old mice.^370^ [^18^F]THK-5351 is a brain imaging agent developed from [^18^F]THK-5117 (another tau-targeting tracer). Harada et al. reported that [^18^F]THK-5351 has a greater binding affinity for AD brain hippocampal homogenates and elutes faster from the white matter tissue than [^18^F]THK-5117 does. Thus, [^18^F]THK-5351 resulted in greater contrast and lower subcortical white matter retention than did [^18^F]THK-5117.^371^ Kobayashi et al. reported that the number of reactive astrocytes in the postmortem brain was proportional to the uptake of [^18^F]THK-5351 in vivo.^372^ [^18^F]MK-6240 is a promising tau-targeting radiotracer that is being investigated in a phase III clinical trial. Pascoal et al. reported that [^18^F]MK-6240 showed favourable kinetics with rapid brain delivery and washout in vivo.^373^ Hostetler et al. revealed that [^18^F]MK-6240 exhibited rapid stabilization of its distribution volume and good tracer kinetics. Off-target side effects were not observed in self-blocking studies on rhesus monkeys.^374^ Kramer et al. synthesized [^18^F]N-methyl lansoprazole ([^18^F]NML) via a novel ^18^F-labelling method. In a clinical study conducted on 11 patients, the authors reported that although the high affinity of this tracer for tau protein aggregates in vitro, it did not yield positive results in vivo and exhibited low brain retention in AD patients with mild cognitive impairment (MCI).^375^ [^18^F]PI-2620 is another potent agent currently in a phase III clinical trial. A study involving 36 patients with progressive supranuclear palsy (PSP) revealed that patients with PSP can be identified by visual assessment uptake with 68% accuracy compared with 80% accuracy in the normal control group.^376^ In AD patients, tracer uptake was significantly greater than that in healthy individuals.^377^

To date, there is only one FDA-approved tau-targeting radiopharmaceutical has been developed, which has attracted the development of more effective radiopharmaceuticals by pharmaceutical companies. [^18^F]MK-6240 and [^18^F]PI-2620 are two novel ^18^F-fluorinated BBB-penetrating tau-specific brain imaging agents that distinguish AD patients from normal subjects. However, they still maintain certain forms of off-target binding which deserves careful consideration.

#### Translocator protein (TSPO)

TSPO is an 18 kDa protein that is expressed mainly on the mitochondrial membrane and is involved in the translocation of cholesterol from the outer membrane of mitochondria to the inner membrane. TSPO is involved in immunomodulation, apoptosis, mitochondrial metabolism, cell oxidative processes, protein import and ion transport.^378^ Compared with the resting brain microglia, activated brain microglia presented increased TSPO expression. As a type of macrophage that resides in the brain and CNS, microglia are responsible for monitoring and modulating the brain microenvironment through changes in their morphology and secretion of various factors to combat infection or heal brain injury.^379^ Therefore, the activated state of brain microglia highly corresponds to neuroinflammation. Neuroinflammatory responses are also observed in AD, PD, ALS, MS, MDD, OCD, and other nervous system diseases, suggesting that TSPO imaging tracers are potential tools for monitoring neurodegenerative inflammatory pathology.

Currently, several TSPO-targeting radiopharmaceuticals have been discovered and introduced in clinical trials to evaluate their feasibility for application in human brains. The first developed TSPO-targeting imaging agent [^11^C]PK11195 exhibited a K_d_ value of 0.6 nM for rodent TPSO. However, the K_d_ value for human TSPO ranged from 2.3 to 28.5 nM. Clinical studies have demonstrated a strong correlation between increased [^11^C]PK11195 binding and the presence of amyloid deposits in AD patients, inflammatory lesions in MCI patients, and reduced glucose metabolism in patients with multiple neurodegenerative disorders.^380^ Although the positive results of [^11^C]PK11195 in human studies have been confirmed, its clinical use has been hampered by various limitations, including low binding affinity and selectivity for human TSPO, a short half-life of ^11^C (20 min), difficulty in crossing the BBB and poor in vivo stability.

Second-generation TSPO imaging tracers with improved binding potential to human TSPO, decreased off-target binding, and increased brain uptake have been developed in recent years. Here, we provide an overview of the following agents that have been introduced in clinical studies, with a focus on their progress in the past two years: [^11^C]PBR28, [^11^C]DPA-713, [^18^F]DPA-714, [^18^F]FEPPA and [^18^F]PBR111. Compared with [^11^C]PK11195, [^11^C]PBR28 exhibited an 80-fold increase in binding affinity in rhesus macaques, enabling a high signal-to-noise ratio and low nonspecific target. Although [^11^C]PBR28 has been clinically evaluated in patients with neurodegenerative diseases,^381,382^ it has also been used in PET/CT imaging for other neuroinflammation and injury-related disorders. Raval and colleagues performed the first in vivo assessment of [^11^C]PBR28 in patients with alcohol use disorders and reported a positive correlation between the neuroimmune response and alcohol intoxication.^383^ [^11^C]PBR28 also showed increased uptake in the infarcted area of the brain in stroke patients.^384^ [^18^F]FEPPA, a novel ^18^F-fluorinated TSPO-targeting imaging agent, is a fluoroethoxy analogue of PBR28. [^18^F]FEPPA has been used to assess neuropsychiatric diseases; it shows increased binding in the white and grey matter of AD patients, and elevated uptake in the cortico-striatal-thalamic circuit involving the orbitofrontal cortex of OCD patients.^385^ [^11^C]DPA-713 also shows a relatively high signal‒to‒noise ratio and selective binding to human TSPO. [^11^C]DPA-713 could be used to examine microglial activation in AD and PD patients. Currently, several studies have confirmed the association between epilepsy and cognitive dysfunction pathologies with neuroinflammation by [^11^C]DPA-713.^386,387^ The replacement of carbon-11 with fluorine-18 and the relative modification of [^11^C]DPA-713 generate [^18^F]DPA-714. In recent years, [^18^F]DPA-714 has been used for the diagnosis of multiple diseases, including stroke, autoimmune limbic encephalitis (ALE) and drug-resistant focal epilepsy.^388–390^ Notably, [^18^F]DPA-714 has the potential to detect microglial activation and reactive gliosis, which are related to the innate immunity of patients with CD8 + T-cell mediated ALE; this may provide a novel modality to assess these patients, considering that the diagnosis of ALE is a challenge in clinical practice.^391^ Thus, increasing the clinical application of [^18^F]DPA-714 should be a promising future direction. [^18^F]PBR111 exhibited high metabolic stability under in vivo conditions and high binding capacity for the TSPO protein. A clinical study in healthy subjects revealed the high specific uptake of [^18^F]PBR111 in human brains of various ages and genetic groups, thus demonstrating its ability to quantify the TSPO expression level in humans.^392^ [^18^F]PBR111 has also been applied to assess neuroinflammation in the white matter of multiple sclerosis (MS) brains, thus confirming the feasibility of [^18^F]PBR111 for characterizing the immune responses of MS patients.^393^

Exon 4 of the human TSPO gene carries a single nucleotide polymorphism (*rs6971*), which results in the substitution of alanine with threonine. Because of this critical amino acid replacement, *rs6971* strongly influences the binding ability of TSPO-targeting ligands.^394^ Although substantial progress has been made since the development of second-generation TSPO PET imaging agents, the development of novel tracers that exhibit low sensitivity to the *rs6971* polymorphism is essential. Third-generation TSPO PET tracers, including [^18^F]GE180, [^11^C]ER176, and [^18^F]SF12051, are insensitive to the rs6971 polymorphism, necessitating clinical studies to investigate their in vivo performance in human brains. [^18^F]GE180 is characterized by high binding affinity to human TSPO. In addition to the ideal in vivo effect on relapsing-remitting MA and AD,^395,396^ [^18^F]GE180 has also been applied to evaluate TSPO expression and survival rates in patients with glioma, thus suggesting that [^18^F]GE180 could be used to determine the prognosis of patients with recurrent glioma.^397^ [^11^C]ER176 shows high binding capacity to all TSPO *rs6971* polymorphisms, as indicated by PET/CT imaging in healthy subjects with high-affinity binding (Ala/Ala), mixed-affinity binding (Ala/Thr) and low-affinity binding (Thr/Thr). Compared with the second-generation TSPO PET tracer [^11^C]PBR28, [^11^C]ER176 shows greater binding affinity to TSPO and better stability as demonstrated by PET scanning of healthy subjects.^398^ The radiosynthesis method of [^11^C]ER176 in the current good manufacturing practice environment has been developed, which could help increase its production for clinical use.^399^ Currently, a novel third-generation TSPO PET tracer, [^18^F]SF12051, with high binding affinity for TSPO overcomes the limitations of [^11^C]ER176. PET/CT imaging in monkey models demonstrated that [^18^F]SF12051 can be used to quantify TSPO expression with high selectivity and minimal displaceable uptake.^400,401^

Although TSPO is one of the most interesting inflammatory biomarkers, to date, several TSPO radiopharmaceuticals have been employed for cardiovascular imaging, representing a new and valuable opportunity to exploit the potential of PET imaging.^402,403^ [^11^C]PK11195 is used for imaging atherosclerosis, a chronic disease of large and medium-sized arteries involving inflammatory processes that leads to major CVDs, such as ischaemic heart disease, stroke, and peripheral vascular disease.^404^ [^11^C]PK11195 was evaluated in fifteen patients by measuring its uptake, which was calculated as the TBR of activity normalized to venous blood. The results revealed that focal [^11^C]PK11195 uptake in the arterial wall and increased vascular uptake (target-to-background ratio 2.41 ± 1.59 vs. 0.98 ± 0.10; p = 0.001) can be visualized in symptomatic patients.^405^ [^18^F]Fluoromethyl-PBR28 and [^18^F]CB251 were used in a comparative study to evaluate their suitability for myocarditis diagnosis. A comparative study was performed in a rat experimental autoimmune myocarditis (EAM) model.^406^ Regarding targeting specificity, [^18^F]CB251 resulted in more specific TSPO uptake in the hearts of EAM rats than did [^18^F]fluromethyl-PBR28 (1.32-fold greater heart-to-lung uptake ratio versus healthy controls). These results suggest that [^18^F]-CB251 is a sensitive tool for noninvasive diagnosis.^406^ [^18^F]GE180 has been used in the imaging of atherosclerotic plaque inflammation in a mouse model. The highest retention was observed at the 1-minute time point (SUV: 13 ± 2.6), which decreased to 2.8 ± 0.45 at 30 min.^407^ Overall, although the outcomes of cardiac contractility are controversial, these studies may contribute to future studies that explore variable models or drug dosages adopted in different studies.

TSPO-targeting PET tracers have high potential for the diagnosis of not only neurodegenerative disorders but also cancers and cardiovascular diseases, including myocarditis and myocardial infarction. We and other researchers have focused on developing novel TSPO-targeting PET tracers and evaluating their ability to diagnose more diseases. A critical aspect of current third-generation tracers is that they are insensitive to the *rs6971* phenotype. However, the limited knowledge regarding the binding pattern of the targeting ligands to TSPO and disease-related TSPO alterations has hampered the clinical application of TSPO-targeting radiotracers. Therefore, although substantial advances have been made, novel agents that address these issues and meet clinical standards should be discovered.

#### α-Synuclein

α-Synuclein is the major component of Lewy bodies and neurites, which are the pathological properties of degenerating neurons in multiple neurodegenerative diseases.^408^ Various neurodegenerative disorders exhibit distinct locations and filament morphologies of α-synuclein deposition and differ in mutant genes, thus enabling α-synuclein-targeting imaging agents to perform differential diagnosis of PD and other atypical parkinsonism types, such as Lewy body disease (LBD), and multiple system atrophy (MSA). α-Synuclein deposition may occur earlier than the expression of other biomarkers in PD and LBD, thus indicating its potential role in identifying patients with these disorders in the early stage.^409^ Currently, several promising α-synuclein-targeting imaging agents have been developed; only one of them has been evaluated in a human clinical trial, while the remaining tracers have been tested in nonhuman primates.^410^

Ruben et al. reported [^18^F]ACI-12589 on the basis of the Morphomer® library of low-molecular-weight and conformation-specific ligands; this tracer is selective for α-synuclein compared with tau and Aβ. [^18^F]ACI-12589 showed high binding affinity for pathological α-synuclein in different tissues of both PD and MSA patients.^411^ Hansson et.al. from the Clinical Memory Research Unit at Lund University evaluated the characteristics of [^18^F]ACI-12589 in patients with α-synuclein-related diseases, including PD and MSA. [^18^F]ACI-12589 showed differences in binding between MSA patients and healthy participants as well as those with other neurodegenerative diseases. The cerebellar white matter and middle cerebellar peduncles of MSA patients exhibited evident accumulation of the tracer; however, limited binding was observed in PD patients.^412^ The clinical evaluation of [^18^F]ACI-12589 demonstrated its potential for diagnosing MSA and enabled the identification of novel targeted therapies.

Another promising α-synuclein PET tracer is [^18^F]F0502B, which allows the imaging of α-synuclein deposits in related disorders.^413^ They screened the common molecular backbones that are targeted to α-synuclein and synthesized their derivatives and analyzed their binding ability. After screening for binding affinity, autoradiography of the brain sections of mice and PD patients, and PET/CT imaging of nonhuman primates, the researchers discovered that [^18^F]F0502B showed unique selectivity for α-synuclein, with a relatively low binding affinity for tau and Aβ. The atomic structure of the complex formed with the tracer further confirmed the high binding affinity of F0502B for α-synuclein filaments.

Additionally, the α-synuclein PET tracer (d3)-[^11^C]MODAG-001 showed high binding in rodents and pig models injected with α-synuclein preformed fibrils or human brain homogenates from AD patients.^414,415^ The in vivo performance of (d3)-[^11^C]MODAG-001 was evaluated to determine its suitability for clinical translation. α-Synuclein has also emerged as a promising target for diagnosing neurodegenerative disorders involving Lewy bodies.

Although substantial advancements have been made on this topic in recent years, multicenter clinical trials of current PET tracers, such as [^18^F]ACI-12589 and [^18^F]F0502B, should be conducted to provide more evidence of their clinical value. Moreover, a limited number of α-synuclein-targeting PET tracers have been reported. Thus, future studies should focus on discovering new molecules with improved selectivity or on further chemical optimization of the current tracers.

#### Sigma receptors

Sigma receptors are well-adaptive biomarkers in cancer cell imaging. They can be categorized into two subtypes, σ1 and σ2, according to their location of binding in the instinct process of combination with the [^3^H]DTG. Because of their high expression level in tumour cells, a myriad of σR-associated ligands for SPECT and PET/CT imaging have been developed. Since the identification of sigma receptors by Gilbert et al. in 1976, breakthroughs have been achieved in the discovery of sigma targeting radiopharmaceuticals in the past 3 decades.^416^ Although sigma receptor ligands have shown potential efficacy, there are still undiscovered potential therapeutic indications for sigma receptor-targeted therapy combined with SPECT or PET.

The sigma-1 receptor is a chaperone protein that serves as a molecular chaperone and resides mainly in the cell membrane within the mitochondria-associated endoplasmic reticulum (ER). It functions as a mediator between the ER and mitochondria and modulates the Ca^2+^ signalling.^417^ Jia et al. reported direct radiosynthesis of [^18^F]FBFP as a powerful sigma-1 receptor radioligand with favourable properties that was produced from an iodonium ylide precursor in a single step and the first PET/CT imaging evaluation of sigma-1. The results revealed that [^18^F]FBFP showed excellent brain uptake and appropriate tissue kinetics in cynomolgus monkeys.^418^ A number of ligands have been engineered for binding to σ2R. However, only a small number of them exhibit strong selectivity and affinity toward σ2R, and the majority bind to both major isoforms of the sigma receptor with comparable efficacy. The ligands that bind to σ2R can be categorized into several different subtypes: morphan, siramesine-related indole analogues, and benzimidazolone-based ligands. However, although many investigations have been reported, only [^18^F]ISO-1, a member of the benzamide class, has shown excellent in vivo and in vitro performance, which is concomitant with the disadvantage of a lower uptake ratio in low-proliferation tumours (such as BC).^419^ In 2013, Dehdashti et al. evaluated the safety and corresponding dosimetry of [^18^F]ISO-1 for the first time in humans and reported a correlation between [^18^F]ISO-1 and Ki-67. Moreover, the administration of the tracer at a dose as high as 550 MBq was safe, which ensured the relatively safe distribution of the tracer in normal organs.^420^ Nevertheless, despite a number of investigations backing the search for vectors that target σRs, no drugs have yet progressed to the clinical stage. The major reason is most likely the unclear biological effects of σR ligands, which are connected to the involvement of σRs in the etiopathogenesis and pathophysiology of cancer. Therefore, it is essential to address the ADME (absorption, distribution, metabolism, excretion) characteristics and off-target effects in the early phases of drug design.

#### Cannabinoid receptors (CBRs)

CBRs are brain cannabinoid receptors that are classified as GPCRs and are closely correlated with the regulation of mood, hunger, autonomic tone, and cognition.^421^ In a recent study, Radhakrishnan et al. used the [^11^C]OMAR PET imaging to visualize certain brain regions in healthy individuals, aiming to clarify the factors correlated between CB1Rs and gender, age, and body mass index (BMI). They reported a decrease in CB1R availability with increasing age, in men compared with women, and in BMI, which was negatively correlated with CB1R in women but not in men.^422^ In 2010, Terry et al. reported that the density of CBRs could be assessed as the distribution volume (VT) by using the gold standard of compartmental modelling with [^11^C]MePPEP, a PET tracer for CB1 receptors. This study revealed good performance of tracer uptake in the human brain 210 min post injection.^423^

Among type two cannabinoid receptors, CB2Rs presented limited brain expression under physiological conditions compared with the most prevalent GPCRs in the CNS. CB2R is extensively expressed in tissues, cells, and organs related to the immune function, such as spleen, tonsils, and leucocytes. In 2012, Evens et al. conducted a preclinical assessment of [^11^C]NE40 and demonstrated its attractive properties as a radiotracer for visualizing hCB2R via in vivo PET/CT imaging. This study demonstrated the selective binding of [^11^C]NE40 to CB2Rs in the spleen and blood of common rats and revealed high tracer uptake in the brains of rhesus monkeys.^424^ To confirm the biodistribution and radiation dosimetry of [^11^C]NE40, a CB2R-PET tracer, Ahmad et al. examined the safety and tolerability of the tracer in healthy male participants; the results revealed a predicted biodistribution pattern that was consistent with tracer uptake in the lymphoid tissue and appropriate drug kinetics in the healthy human brain.^425^ The utilization of preclinical data in well-designed clinical trials is a significant problem for CBR. The translatability of these results may be hindered by factors such as skewed signalling and the off-target-effects of CBR agonists.

#### Vesicular monoamine transporter type-2 (VMAT2)

VMAT2 is a dopamine presynaptic marker commonly used to identify the integrity of nigrostriatal neurons. VMAT2 is a protein localized at the vesicular membrane and is involved in transporting monoamine transmitters from the cytosol into the synaptic membrane and storing neurotransmitters in human brain neurons.^426^ Patients with PD presented a reduction in VMAT2 expression in the brain striatum, which enables the diagnosis of PD and other disorders based on the abnormal VMAT2 density determined using VMAT2-targeting PET tracers.

[^11^C]dihydrotetrabenazine ([^11^C]DTBZ) is an efficient PET tracer for imaging VMAT2, and it has been successfully evaluated in patients with AD and dementia with Lewy bodies.^427^ Considering the short half-life of carbon-11 (t_1/2_ = 20 min), ^18^F- fluorinated (t_1/2_ = 110 min) fluoropropyl analogues, namely, [^18^F]FP-( + )-DTBZ (also known as [^18^F]AV-133), with better clinical availability for PET/CT imaging of VMAT2, have been developed. Clinical studies of [^18^F]AV-133 in healthy participants confirmed its safety in humans, with satisfactory in vivo biodistribution and radiation dosimetry.^428^ PET/CT imaging showed the apparent binding of [^18^F]AV-133 in the striatum of healthy subjects with an average age of 59.3 ± 6.0 years old, which further confirmed the usefulness of [^18^F]AV-133 for imaging VMAT2 in humans and for the diagnosis of PD and VMAT2-related neurodegenerative disorders in clinical practice.^429^ PET/CT imaging of [^18^F]AV-133 in PD patients demonstrated decreased VMAT2 density in the posterior and anterior putamen of PD patients.^430^ Additionally, in patients with uncertain Parkinsonian syndrome, [^18^F]AV-133 showed altered PET signals in the brain and substantial management impact.^431^ Recently, a clinical study monitored the progression of neurodegenerative diseases over two years by [^18^F]AV-133 PET/CT imaging, indicating the sensitivity of [^18^F]AV-133 in detecting alteration in VMAT2 density in subjects with PD at least 3–6 years before symptom occurrence and in clinical diagnosis, thus making [^18^F]AV-133 a powerful tracer to monitor PD progression over the long term, which could provide better benefits to patients, including early diagnosis and intervention and better prognosis.^432^ VMAT2 is also expressed by the insulin-producing β-cell mass (BCM). Preclinical studies have confirmed the binding of VMAT2 in the rodent pancreas. A modest reduction in [^11^C]DTBZ signals was observed in patients with type 1 diabetes.^433^ Following the discovery of [^18^F]AV-133, PET/CT scans for patients with BCM and type 1 diabetes were reported. The uptake of [^18^F]AV-133 in the pancreas of patients with type 1 diabetes was significantly decreased compared with that in healthy controls, thus suggesting the efficiency of [^18^F]AV-133 in detecting BCM density and the feasibility of this tracer for diagnosing type 1 diabetes in clinical settings.^434^ Moreover, PET/CT scans with [^18^F]AV-133 and its inactive enantiomer [^18^F]DTBZ in healthy subjects and patients with type 1 diabetes have been used to provide a suitable method for measuring [^18^F]AV-133 and VAMT2 binding in vivo.^435^

Several clinical studies have proven the utility and availability of [^18^F]AV-133 in detecting VMAT2 expression, thus making it a promising PET agent for diagnosing PD and type 1 diabetes. Further research studies should focus on accelerating the FDA approval process for [^18^F]AV-133.

#### Synaptic vesicle glycoprotein 2 A (SV2A)

SV2A is an isoform of the synapse vesicle protein, and it is ubiquitously expressed in all synapses. SV2A is located on the secretory vesicles of neurons for trafficking, endocytosis, and exocytosis. Thus, SV2A has been used as a biomarker to investigate synaptic density.^436^ Neurodegenerative diseases with alterations in synaptic density can be detected on the basis of the uptake of SV2A-targeting PET tracers. Several PET tracers with selectivity for SV2A have been reported to show high sensitivity to synapse density in preclinical models.^437–440^ In humans, [^11^C]UCB-J shows a satisfactory capacity to cross the BBB, high binding affinity to SV2A, and low nonspecific binding in the white matter of the brain. This method has been used to examine the loss of synaptic density in patients with Lewy body dementia,^441^ progressive supranuclear palsy (PSP),^442^ AD,^443^ frontotemporal dementia from C9orf72,^444^ HD,^445^ and amnestic mild cognitive impairment (aMCI),^446^ demonstrating that [^11^C]UCB-J is valuable for the early diagnosis of synaptic loss related neurodegenerative disorders and facilitates disease management. Notably, a head-to-head clinical study suggested that, for diagnosing early AD, [^11^C]UCB-J is more sensitive than [^18^F]FDG, a PET tracer widely used in clinical practice.^447^ Subsequently, analogues of [^11^C]UCB-J_,_ i.e., ^18^F-fluorinated SV2A-targeting PET tracers with longer half-lives were developed. [^18^F]UCB-H based PET/CT scans revealed synaptic loss in the right anterior parahippocampal gyrus of the behavioural variant of frontotemporal dementia (bvFTD) patients, thus revealing synaptopathy in bvFTD patients.^448^ However, [^18^F]UCB-H showed lower binding affinity to SV2A than did [^11^C]UCB-J. Another ^18^F-fluorinated SV2A-targeting PET tracer, [^18^F]UCB-J, is difficult to radiosynthesize and is used for routine automated production. Therefore, mono- or difluorinated UCB-J derivates [^18^F]SynVesT-1 ([^18^F]SDM-8) and [^18^F]SynVesT-2 ([^18^F]SDM-2) were discovered and their clinical studies have been reported. [^18^F]SynVesT-1 PET/CT scans in healthy subjects have shown excellent PK with rapid brain uptake and high specific binding capacity in vivo, making it a valuable PET tracer for monitoring SV2A expression and synapse density-related neuropsychiatric disorders.^449,450^ Furthermore, clinical studies of [^18^F]SynVesT-2 have indicated high specific uptake, low nonspecific uptake in the brain, and faster kinetics than those of [^18^F]SynVesT-1 and [^11^C]UCB-J.^451^

Because SV2A is highly expressed in all synapses, it can be used to diagnose multiple neurodegenerative disorders. In addition to typical radiotracers such as [^18^F]UCB-J, novel SV2A PET tracers including [^18^F]SynVesT-1 and [^18^F]SynVesT-2, have excellent PKs in humans. Hence, further studies should focus on evaluating the in vivo performance of these novel tracers in patients with neuropsychiatric diseases rather than in healthy subjects.

#### Monoacylglycerol lipase (MAGL)

MAGL, a serine hydrolase, is a critical enzyme in the endocannabinoid (eCB) system. As a lipid signalling network in the central and peripheral systems, eCBs are regulated by two main cannabinoid receptors, namely CB1 and CB2. Two endogenous ligands, i.e., anandamide (AEA) and 2-arachidonoylglycerol (2-AG), bind to and stimulate CB1 and CB2, respectively. MAGL catalyzes and hydrolyses 2-AG to generate arachidonic acid (AA), which constitutes more than 50% of the total AA in human brains. AA and its eicosanoid metabolites are strongly associated with the occurrence of neuroinflammation, resulting in the development of neurodegenerative disorders, including AD, PD, and Huntington’s disease (HD). Therefore, the accumulation of AA in the CNS suggests the overexpression of MAGL, which has been identified as a pathogenetic mechanism of neurodegenerative diseases.^452^ Additionally, the high expression of MAGL in human tumour cells promotes cancer invasion, migration, progression and tumourigenesis through the fatty acid network.^453^

[^18^F]T-401 is the only MAGL-targeting radiotracer for application in human PET/CT scans, showing reversible binding to MAGL with a high contrast PET signal. PET/CT imaging and blocking studies of [^18^F]T-401 in rhesus monkeys revealed that [^18^F]T-401 is a valuable tracer for the evaluation and quantification of MAGL in healthy and diseased monkey brains.^454^ The first-in-human PET/CT scans of [^18^F]T-401 in healthy participants demonstrated rapid brain uptake and reversible radioligand kinetics. The distribution volume (*V*_T_) of [^18^F]T-401 is consistent with the MAGL expression pattern in human brains, where the tracer showed the highest uptake in the cerebral cortex, modest accumulation in the thalamus and putamen, and the lowest uptake in the brainstem and white matter.^455^ This initial clinical study confirmed the potential of [^18^F]T-401 for monitoring MAGL expression in the human CNS.

In addition to [^18^F]T-401, we and other groups have reported MAGL PET tracers that have been applied to PET/CT imaging in monkeys, including [^11^C]PF-06809247, [^11^C]SAR127303, and [^18^F]MAGL-2102. The glycol-derived irreversible MAGL PET tracer [^11^C]PF-06809247 shows high selective binding affinity to MAGL, with an IC_50_ value of 13 nM. The rodent and non-human primate PET/CT scans of [^11^C]PF-06809247 revealed satisfactory brain uptake, low-nonspecific binding, and suitable whole-body distribution, thus confirming its essential role in cross-species measurements of brain MAGL expression.^456^ [^11^C]SAR127303 is a sulfonamido-based MAGL-targeting PET tracer, and its precursor SAR127303 is a known MAGL inhibitor with an IC_50_ value of 39.3 nM. A study in mice revealed suitable ex vivo biodistribution, brain uptake and in vivo clearance. PET/CT imaging in non-human primates further demonstrated the high specificity and permeability of [^11^C]SAR127303 in monkey brains.^457,458^ We also reported a reversible MAGL-targeting PET tracer, namely, [^18^F]MAGL-2102, which was designed on the basis of the piperazinyl azetidine scaffold. PET/CT imaging in rodents and monkeys revealed that this tracer has favourable brain permeability, high selective binding capacity, heterogeneous radioactivity distribution, and ideal brain kinetics, thus suggesting high potential for application in human disease diagnosis.^459^

As promising MAGL-targeting PET tracers with high relevance for successful clinical translation have been developed, further studies should focus on their clinical evaluation. For [^18^F]T-401, PET/CT scans in patients with neurodegenerative disorders are critical for identifying of its availability in clinical diagnosis. Furthermore, considering the critical role of MAGL in cancer pathogenesis, PET/CT imaging of tumour MAGL should be performed by using current radiotracers to investigate their availability in cancer theranostics.

#### Fatty acid amide hydrolase (FAAH)

FAAH is located on neurons and non-neuronal cells in the brain and plays a critical role in the degradation of bioactive fatty acid amides such as 2-arachidonoylglycerol (2-AG) and N-arachidonoylethanolamide(AEA).^460^ FAAH, combined with AEA and 2-AG, constitutes the endocannabinoid system (ECS), which has an essential role in modulating CNS energy balance and peripheral nervous system metabolism in the human body.^461^ FAAH is a potent target in a variety of human neuron related diseases, including pain, anxiety disorder, depression, epilepsy, schizophrenia, and posttraumatic stress disorder (PTSD).^462–467^ Over the past few decades, several radiotracers for PET imaging of FAAH have been developed for preclinical and clinical evaluation,^468–470^ including the irreversible tracer [^11^C]CURB and the reversible tracer [^11^C]MK-3168, which have been evaluated in humans.^471^

[^11^C]CURB, also known as URB694, is the first reported irreversible radiotracer for the successful monitoring of in vivo FAAH. URB694 is derived from its close analogue URB597, a well-studied FAAH inhibitor.^472^ Owing to its ideal brain penetration ability and plasma stability, [^11^C]CURB has been recently applied to rodents and humans with cannabis addiction, alcohol use disorders, borderline personalities, and psychiatric disorders.^471^ Kolla et al. used [^11^C]CURB PET/CT scans to investigate the expression level of FAAH in 16 individuals with antisocial personality disorder (ASPD). The results revealed a lower FAAH density in the amygdala of individuals with ASPD, and this decrease was inversely correlated with the incidence of aggressive behaviour in the cerebellum and striatum of those with ASPD.^473^ Moreover, Watts et al. performed PET/CT imaging via [^11^C]CURB to detect FAAH activity in young adults with psychotic disorders, and the results revealed that increased FAAH activity was associated with decreased hippocampus volume and increased hippocampal glutamate levels.^474^

[^11^C]MK-3168 is the first reported reversible FAAH-targeting PET tracer that exhibits excellent potency and selectivity, reasonable lipophilicity, and amenability for radiolabelling. The in vivo performance of [^11^C]MK-3168 in humans and non-human primates has been reported. Postnov et al. conducted a PET study with [^11^C]MK-3168 to investigate brain FAAH occupancy after single and multiple doses of JNJ-42165279 (an oral selective FAAH inhibitor). The results demonstrated that single doses of JNJ-42165279 as low as 10 mg resulted in >95% occupancy.^475^ Additionally, Liu et al. reported that [^11^C]MK-3168 exhibited good brain penetration and FAAH-specific binding in rhesus monkeys. Thus, [^11^C]MK-3168 could serve as a suitable PET tracer for detecting FAAH in the brain.^476^

As a serine hydrolase, FAAH is involved in the biosynthesis and metabolism of endocannabinoids and other lipids. Covalent and reversible FAAH inhibitors, which have the potential to be developed as PET tracers, have been reported. Given that PET tracers targeting FAAH are mainly ^11^C-labelled agents, more ^18^F-fluorinated tracers should be more actively developed because of their longer half-life than that of carbon-11, allowing for longer scan times and diverse radiolabelling procedures. Besides the number of these radiopharmaceuticals is limited, and further studies on these radiopharmaceuticals and their close analogues in higher species are needed.^471^

#### Alpha-amino-3-hydroxy-5-methyl-4-isoxazole propionic acid receptor (AMPAR)

AMPAR is a class of ionotropic glutamate receptor that enables rapid excitatory neurotransmission in the brain. Glutamate an excitatory neurotransmitter, is released by synaptic terminals and, acts on AMPARs. Additionally, glutamate plays a critical role in MDD.^477^ An increase in AMPAR levels is associated with neurobiological changes in MDD.^478^ Therefore, to improve our understanding of AMPAR related disorders in the CNS, imaging and quantifying AMPAR in vivo are necessary.^479–481^ The PET tracers [^11^C]K-2, [^11^C]HMS011, and [^18^F]AMPA-2109 have been successfully developed and are currently being evaluated in humans and monkeys.

[^11^C]K-2, a derivative of 4-[2-(phenylsulphonylamino)ethylthio]-2,6-difluorophenoxyacetami-de (PEPA), was the first radiotracer to be developed for visualizing and quantifying AMPAR levels in the human brain.^482^ PET/CT imaging in rats revealed that [^11^C]K-2 represented rapid brain uptake correlated with 200% of the standardized uptake value, and it specifically binds to AMPAR, as indicated by regional differences among different brain regions and a strong positive correlation with AMPAR expression.^483^ [^11^C]K-2 is metabolized to [^11^C]K-2OH (a hydrolysate of [^11^C]K-2) within 10 min post-injection, and it can cross the BBB through paracellular transport, thus, exhibiting AMPAR signals at the cell surface.^484^ PET/CT imaging of [^11^C]K-2 in patients with MDD and bipolar disorder (BD) demonstrated that the standardized uptake value ratio(SUVR) in the cerebellum was greater in MDD patients than in BD patients, whereas the SUVR level was greater in the parietal lobe and postcentral gyrus of BD patients.^485^ Another study demonstrated that PET/CT imaging of AMPARs in patients with psychiatric disorders via [^11^C]K-2 enabled the detection of the symptomatology scores of disease due to differences in AMPAR density between patients and healthy volunteers.^486^

[^11^C]HMS011 is a radiolabelled variant of perampanel, which is an antiepileptic medication applied to in vivo animal imaging by targeting AMPARs.^487^ Several in vivo studies conducted on rats, monkeys, and humans have demonstrated the promising properties of [^11^C]HMS011. A previous study revealed substantial uptake of [^11^C]HMS011 in rat and monkey brains.^487^ Takahata et al. performed human PET/CT scans via [^11^C]HMS011, and the results revealed quick brain uptake (SUV 2.0–2.9), rapid clearance of the tracer, and notable individual variability (one subject showed prolonged radioactivity retention in the brain than the other individuals did).^488^ [^18^F]AMPA-2109 is another PET radiotracer derived from the AMPAR positive allosteric modulator (PAM). Chen et al. developed [^18^F]AMPA-2109 through molecular docking studies and CNS PET multiparameter optimization (MPO) analysis, which demonstrated excellent BBB penetration and high brain uptake.^489^

While a plenty of efforts have been devoted to imaging the CNS, the development of AMPAR PET tracers is still in its early stages.. Owing to the widespread distribution of AMPARs, existing PET tracers are hampered by several limitations, including low BBB penetration, high nonspecific binding or low binding potential, which make them unsuitable for preclinical and clinical studies. Moreover, the widespread use of AMPARs leads to severe adverse effects; therefore, the development of subtype-selective agonists or antagonists of PET tracers for AMPARs is promising and important. Another critical issue is improving the affinity of radiotracers for AMPAR, which has a low density in the brain, which could enable more precise in vivo imaging of AMPAR.^490^

## Evolving radiation biological effects of radiopharmaceuticals

**Fig. 9:**
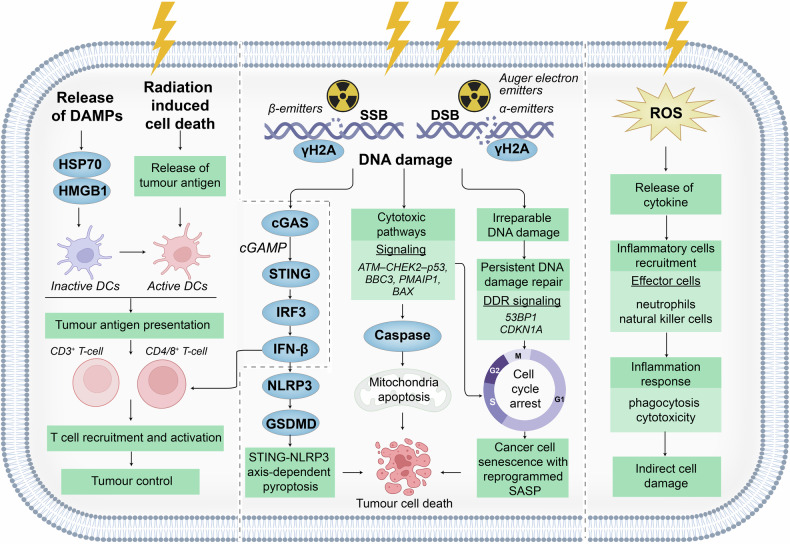
The potential biological mechanism of radiopharmaceutical-induced cell death. Radiopharmaceuticals induce SSBs (β-emitters) or DSBs (α-emitters and Auger electron emitters), resulting in cancer cell senescence via the SASP, mitochondrial apoptosis, and STING-NLRP3 axis-dependent pyroptosis. Radiopharmaceuticals reshape the tumour immune microenvironment through three mechanisms. First, radiopharmaceuticals induce the release of “danger signals” (also known as danger-associated molecular patterns), including HSP70 and HMGB1, which are essential for the activation of DCs. Second, radiation-induced cell death stimulates the release of tumour antigens, which are presented by activated DCs. The cGAS-STING pathway also activates cytotoxic T cells through the interferon response. Therefore, T cells are recruited to the TME and activated to induce immunogenic cancer cell death. Radiopharmaceuticals also induce the production of ROS, leading to cell apoptosis and an inflammatory response. The mechanisms are concluded from studies of radiopharmaceuticals or learned from the research of radiotherapy. Part of this figure was created with Biorender.com

The therapeutic effect of radiopharmaceuticals is achieved through the ionizing radiation produced by radionuclides, resulting in DNA damage and targeted cell killing. Although there is insufficient knowledge about the precise mechanism and unique elements underlying RPT, various studies have revealed multiple critical cellular processes involved in radiation-induced cell death resulting from radiotherapy. This section aims to provide an overview of current progress in understanding the therapeutic mechanism of radiopharmaceuticals. We also hypothesize potential mechanisms underlying radiopharmaceutical-induced cytotoxicity, including direct DNA cell death, ROS-mediated cell death, and immunogenic cell death based on the knowledge of radiotherapy caused biological effects **(**Fig. 9**)**.

### Direct and ROS-mediated tumour cell damage

The ionizing radiation generated by radionuclides causes single-strand breaks (SSBs) and double-strand breaks (DSBs), which induce direct tumour cell death. Various radionuclides cause different types of DNA damage depending on their emission activity. α-Particle emitters, including radium-223, astatine-211, actinium-225, bismuth-213, and lead-212,^491–493^ have a high energy range (4–8 MeV), short emission range (28–100 μm) and high LET (50–230 keV/μm).^494,495^ Widespread DNA damage can occur if α-emitters are used at high doses. Low to moderate doses are sufficient to induce irreversible DSBs.^496^ Radium-223 has been approved as the pioneering targeted radiopharmaceutical therapy to treat prostate cancer; it activates the caspase 3/7 pathway and leads to the programmed death of cancer cells.^497^ Auger electron emitters, including indium-111, iodine-123, iodine-125, platinum-191 and gallium-67, have extremely low-energy ranges (50–2300 keV) and high LET (4–26 keV/μm), causing lethal damage to cells through DSBs rather than SSBs.^498,499^ Similarly, previous studies have shown that Auger electron emmiter radiation is a primary cause of direct DNA damage, with less emphasis on ROS-induced indirect effects, and this DNA damage is attributable to the characteristics of Auger electron emitters.^500^ A pioneering study demonstrated that iodine-125 induced DSBs within a 10-base pair region surrounding the site of decay.^501^ In contrast to the α-emitters mentioned above, β- emitters, including lutetium-177, holmium-166, copper-67, strontium-89, yttrium-90, and iodine-131,^502–504^ which have been which have been utilized in preclinical and clinical cancer treatment, exhibit low LET (0.1–10 keV/μm).^505^ The main mechanism of β-emitter involves SSBs and minor chemical modifications of DNA bases.^505^ β-emitters cause indirect DNA damage because of the generation of reactive oxygen species (ROS) and subsequent oxidative stress.^13^ The biological effects of lutetium-177 have been widely investigated. Researchers have assessed this mechanism on the basis of level of γH2AX/pATM. A slightly increased γH2AX/pATM level reflected that [^177^Lu]Lu-DOTA-TATE induces indirect DNA damage and SSBs.^506^ Another study revealed the occurrence of irreversible DNA DSBs after treatment with [^177^Lu]Lu-DOTA-JR11, and the formation of γH2AXs was increased.^507^

A recent study revealed that DSBs caused by α-emitter exposure activate stimulator of interferon genes (STING)-NLRP3 axis-dependent pyroptosis.^16^ We recently reported that ^211^At-based α-radiopharmaceuticals result in irreparable DNA damage and consequently activate persistent DNA damage repair signalling, including 53BP1, which triggers cell cycle arrest. The decreased expression of target proteins and cell senescence induce a reprogrammed senescence-associated secretory phenotype (SASP), as indicated by the increased expression of SASP factors, including CXC chemokine ligand 8 (CXCL8), interleukin-6 (IL-6), vascular endothelial growth factor A (VEGFA), tumour necrosis factor alpha (TNF-α) and MET.^11^ Furthermore, learning from the biological effects of radiotherapy, DNA repair also stimulates cytotoxic pathways through the ATM-CHEK2-p53 pathway. Activated p53 induces the accumulation of caspase signals and then triggers mitochondria-dependent apoptosis.^508^ Additionally, radiation also leads to the generation of ROS, which induce indirect cell death. Studies have shown that plutonium-28 induces DNA damage both directly and indirectly, by forming hydroxyl radicals and generating ROS respectively.^509^ ROS attract and recruit effector cells, including neutrophils and natural killer cells, which induce phagocytic and cytotoxic responses to cause cell damage.^510^

### DNA repair pathways

DNA repair pathways cannot fully exert their function in cancer cells because of the down-regulation of their signalling, thus causing uncontrolled proliferation. Therefore, tumour cells are more susceptible to radiopharmaceuticals, causing severe DNA damage through the high energy or LET of radionuclides.^511^ Different types of DNA damage activate different DNA repair pathways. Cancer cells mainly repair SSB damage via base nucleotide excision repair (NER) and base excision repair (BER). For DSBs, non-homologous end joining (NHEJ) and homologous recombination (HR) play vital roles in DNA repair.^512^ Owing to multiple DNA repair mechanisms, resistance to β-emitter and α-emitter-based cancer therapies has been observed.^513,514^ There are several potential signals that control the DNA repair pathway, such as poly ADP‒ribose polymerase (PARP).^423^ Considering that PARP inhibitors (PARPis) may assist radiopharmaceuticals in blocking DNA damage repair, researchers are exploring combined therapies. Moreover, PARPis, which bind to the PARP protein on DNA, can be effectively used as targets for radiopharmaceuticals.^515^

### Dose rate

The dose rate (DR) plays a key role in RPTs, through modulats the cell behavior.^13^ Radiation is more harmful at high DRs than at low DRs. Thus, if an equivalent dose is injected into patients, many forms of DNA damage can occur at high DRs rather than at low DRs.^516,517^ Another phenomenon is that by accessing radiation-induced foci (RIF) cells exposed to high DR, a linear increase in γ-H2AX foci can be observed.^518^ These findings reveal that low DR is likely to induce DNA damage that can be repaired while the dose is delivered. However, some studies have demonstrated that DNA repair mechanisms are not directly related to DR; instead, the extent of DNA damage caused by the energy of radiation, including LET and dose, plays a vital role.^519,520^

### Reshaping the tumour immune microenvironment

In addition to direct and ROS-mediated DNA damage to targeted cells, an increasing number of studies have confirmed that immunogenic cell death occurs following radiopharmaceutical administration. These observations involve the attraction and activation of dendritic cells (DCs), cytotoxic T cells and other immunogenic cells in the tumour microenvironment, which stimulate the immune-response against tumour cells.^14,15^ Several studies have attempted to elucidate the biological processes that participate in radiation-induced immune stimulation. Gaschet et al. reported that bismuth-213 irradiation of tumour cells stimulates “danger signals” (also known as danger associated molecular patterns, (DAMPs)), including heat shock protein 70 (Hsp70) and homoeostatic group box protein 1 (HMGB1), which are essential for the activation of DCs, which present tumour- related antigens to immune cells to stimulate immunogenic cell killing.^14^ Other α-particles such as radium-223, also induce a similar immune response, and enhance memory immunity against tumour cells.^16^ Another mechanism underlying the immune-stimulation of radiopharmaceuticals is the consequence of radiation-induced direct cell death. Tumour cells release a plenty of tumour specific antigens after being killed by radiation, which further improves the presentation of antigens to DCs and leads to the activation of the immune-microenvironment; this positive regulatory effect results in effective cell killing. The cyclic GMP-AMP synthase (cGAS)-STING pathway also plays an important role in the propagation of radiation-induced immune responses. Unlike the STING-NLRP3 pathway induced pyroptosis mentioned above, the interferon response activated by the cGAS-STING pathway also triggers the activation of CD8^+^ T cells, leading to tumour control.^521^

The potential of radiopharmaceuticals for tumour-microenvironment stimulation makes them promising immune modulating strategies to improve the anti-tumour efficacy of immunotherapy. Multiple studies have confirmed that the combination of RPT and ICB therapy further improved therapeutic efficacy compared with that of ICB alone, further demonstrating that the RPT increased the response to ICB.^522–524^ Similarly, compared with monotherapy, RPTs combined with CAR-T-cell immunotherapy improve OS and progression-free survival (PFS).^525^

## Perspective and conclusion

Radiopharmaceuticals have been investigated for at least 80 years, opening a new era of clinical application. However, what is the next milestone? Although radiotheranostics have been proven to be a powerful tool for rapid imaging and effective elimination of tumour cells, they remain a second- or third-line modality in clinical practice, where the first choice remains chemotherapy or radiotherapy for treatment and CT or MRI for diagnosis. Therefore, there is still a large gap between basic research and clinical translation. To address this, multidisciplinary knowledge should be applied to radiopharmaceutical discovery, and cooperation among multiple organizations is needed.

First, the disease targets for FDA-approved radiopharmaceuticals are limited. SSTR and PSMA are two targets approved for tumour radiotheranostics. FAP-targeting radiopharmaceuticals for multiple cancers may become the next promising “blockbuster” drugs. Additionally, Aβ- and tau-targeting radiolabelled imaging agents are used for the diagnosis of neurodegenerative disorders in clinical practice. Although various disease targets have been introduced for the development of novel radiopharmaceuticals, more clinical studies need to be conducted to elucidate their performance in patients. Additionally, market assessment is crucial for evaluating the expected profits of these radiopharmaceuticals following approval. Several promising targets, such as HER2, have shown promising outcomes for the treatment of BC.^526^ However, it is still unclear whether these targets are suitable for radiopharmaceuticals due to the sucessful discovery of HER2 targeted antibodies. It is believed that identifying a target associated with a disease that is challenging to treat based on the current modalities like chemotherapy or radiotherapy could lead to the development of more effective radiopharmaceuticals. Additionally, a major concern with current imaging agents used in PET/CT or SPECT/CT is the difficulty in distinguishing between disease lesions and inflammation, which is observed in both cancer and neurological imaging. Hence, it is crucial to identify more precise targets that are exclusively found in targeted lesions.

Radiopharmaceuticals are not only beneficial for diagnosing and treating cancer through radiotheranostics but also play a crucial role in diagnosing neurodegenerative disorders and cardiovascular diseases. Numerous well-designed radiotracers have been developed for investigating neurodegenerative disorders, several of which have been approved and integrated into clinical practice to help select appropriate therapies for patients.^527–529^ However, the complexity of neuropathological changes in brain abnormalities, such as overlapping characteristics between different types of neurodegenerative conditions, can lead to diagnostic ambiguity. Therefore, the use of multiple PET radiopharmaceuticals to assess these neuropathological processes is often recommended.^529^ However, this approach has its limitations, particularly in detecting early-stage abnormal amyloid and tau proteins on PET scans. Additionally, relying on multiple PET scans can increase healthcare costs, impose financial burdens on patients, and create logistical challenges. The growing variety of brain PET radiopharmaceuticals used in clinical settings reflects the complexity of dementia. Advances in PET imaging for characterizing dementia are expected to deepen our understanding of its underlying pathophysiology and aid in evaluating new therapies.^530^ SPECT and PET imaging are also commonly used to assess cardiovascular function and related conditions, such as myocardial perfusion, atherosclerosis, and heart failure.^531–533^ These imaging techniques can also detect prosthetic valve endocarditis,^534^ implanted device infections,^535^ and other heart-related issues.^536^ Despite the success of radiopharmaceutical imaging in cardiovascular disease, challenges remain, particularly in developing targeted radiopharmaceuticals with low myocardial signals. Moreover, there is a pressing need to identify biomarkers for the early detection of cardiovascular pathology and to develop specific radiotracers for these biomarkers. Notably, advancements in hybrid cardiac PET/MRI, image reconstruction algorithms, and artificial intelligence-driven image analysis are expected to enhance cardiovascular imaging and support the development of new radiopharmaceuticals in the near future.

One of the challenges in the field of targeted radiotheranostics is to improve the binding affinity and pharmacokinetic properties of targeting vectors for effectively deliver radionuclides. Many studies have focused on modifying existing radiolabelled imaging agents or developing new targeting ligands to enhance uptake in lesions, extend retention time, and reduce toxicity to normal organs.^537–539^ Various high-throughput screening platforms, such as phage display, mRNA display, and DNA-encoded compound libraries, have been utilized to identify targeting ligands with strong binding affinity. Recently, RayzeBio and PeptiDream have reported the development of a GPC3-targeting radiopharmaceutical, RAYZ-8009, labelled with ^68^Ga, ^177^Lu, and ^225^Ac. This radiopharmaceutical has shown high uptake in hepatic carcinoma and significant anti-tumour effects.^540^ The cyclic peptide RAYZ-8009, with an IC_50_ of approximately 10 nM, was discovered through an mRNA display platform. However, further optimization is needed to improve the binding affinity of the targeting molecule for increased lesion uptake. Chemical modification strategies play a crucial role in the development of next-generation radiopharmaceuticals, as highlighted by current studies. These strategies may include the use of covalent warhead incorporation,^541^ unnatural amino acid replacement,^542^ and cyclic peptides based on various motifs.^543^ For instance, adding covalent warheads to radioligands can improve specific binding and retention time by forming a covalent chemical bond with disease targets, rather than relying on reversible recognition based on configuration.^541^ Improving binding affinity is essential to prevent false-positive diagnoses, which is a common challenge in the field. Radiolabelled imaging agents with high binding affinities can help differentiate between inflammatory lesions and tumour lesions based on their different expression levels.

To enhance the retention and accumulation of radiopharmaceuticals in disease lesions, particularly for RPTs, improving their stability and circulation time in the body is a common approach. One effective strategy is the use of peptide self-assembly triggered by enzymes that are overexpressed in tumours. This helps to prolong the presence of radionuclides in the affected areas and prevents them from being quickly cleared from the lesions.^544^ Various enzyme-induced aggregation systems, such as alkaline phosphatase, tyrosinase, and cathepsin enzymes, have shown promising results in promoting self-assembly in tumour tissues.^545–547^ New methods that can specifically achieve the accumulation of radiopharmaceuticals in tumours are needed, especially for developing long-acting RPTs.

The uptake and accumulation of radiopharmaceuticals in non-target organs have raised concerns about potential toxicity and safety issues in clinical settings. To address this, a pretargeting strategy based on bioorthogonal and click chemistry could be utilized to achieve precise and safe delivery of radionuclides to specific disease targets.^548^ This strategy involves using a targeting ligand modified with a bioorthogonal group as a prodrug to target disease sites, followed by a radiolabelled molecule with a complementary group for covalent binding to the targeting ligands. This approach helps to avoid radiation damage to normal organs.^549^ In addition, a protease-releasable masking technique using peptides to mask the binding site of the targeting antibody has been explored to reduce toxicity on non-target tissues.^550^ The masking peptide can be removed when cleavage by the overexpressed protease, exposing the critical binding site of the targeting antibody and activating the radiopharmaceuticals with precision. While this masking methodology shows promise in enhancing safety and reducing normal organ toxicity, it has not yet been applied in radiopharmaceutical research. It is noteworthy that while the development of high value peptide ligand is underway, it remains critical to set up a productive drug screening platform for the progression of next-generation radiopharmaceutical discovery. The utilization of new preclinical models such as patient-derived organoids could offer opportunities for conducting rapid and effective radiopharmaceutical screenings.^551^

There are different requirements for radiopharmaceuticals used in diagnosis compared to those used in radiotherapeutic procedures. Diagnostic radiotracers need to be rapidly cleared from the body, especially for PET/CT imaging of neurodegenerative diseases. On the other hand, RPTs require radiopharmaceuticals that can stay in diseased lesions for an extended period to effectively eliminate tumour cells. Although short half-life radionuclides are typically used for diagnostic purposes and long half-life radionuclides for radiotherapeutic treatments, it is important to develop targeting ligands that can fulfil both diagnostic and therapeutic requirements. A growing trend in the field is the utilization of radiotheranostic pairs, where the same targeting ligand is used for the development of the next generation of radiopharmaceuticals. One approach to achieving both rapid clearance for imaging and long-lasting tumour therapy in a single molecule is through chemical methods, such as bioorthogonal chemistry, which allow for controlled switching of a rapid excretion imaging agent to a long-acting therapeutic radiopharmaceutical.^552^ This can be accomplished by incorporating a chemistry-activated self-assembly or polymerization motif.

The development of next-generation radiopharmaceuticals should meet clinical needs in several key areas. First, how can we achieve accurate and early-stage diagnosis for diseases that cannot be detected with current methods? Second, how can we improve the prognosis for cancer patients who do not respond to existing treatments such as chemotherapy, radiotherapy, immune checkpoint blockade, or targeted therapy? To usher in a new era of radiopharmaceuticals, collaboration among clinicians and researchers from diverse backgrounds is essential. Chemists play a crucial role in creating more effective targeting ligands for radiopharmaceuticals. Nuclear physicists can facilitate the production of various radioisotopes with high purity and the capability for in-house generation. Alpha particles, with their short emission range and high LET, show significant promise in RPT. However, the large-scale production of alpha particles, including astatine-211 and actinium-225, remains challenging due to limited resources. Therefore, establishing an efficient nuclear reactor that maximizes radionuclide yield using limited resources is critical. Device engineers are needed to develop high-resolution nuclear imaging equipment, which can enable the use of radiolabeled agents for hyper-resolution and hyper-sensitivity imaging, and even subcellular imaging. Finally, clinicians specializing in oncology, neurodegenerative disorders, cardiovascular diseases, and nuclear medicine must collaborate to expand the clinical applications of radiopharmaceuticals across various fields.

This review offers a comprehensive summary of the latest developments in radiopharmaceuticals, covering both preclinical and clinical research, and setting out potential pathways for the development of innovative next-generation radiopharmaceuticals. These breakthroughs are anticipated to result in groundbreaking radiopharmaceuticals, presenting promising alternatives for patients with serious illnesses.
